# *RAD* gene family analysis in cotton provides some key genes for flowering and stress tolerance in upland cotton *G. hirsutum*

**DOI:** 10.1186/s12864-021-08248-z

**Published:** 2022-01-10

**Authors:** Nosheen Kabir, Xin Zhang, Le Liu, Ghulam Qanmber, Lian Zhang, Yu Xuan Wang, Zhuojing Sun, Na Zhao, Gang Wang

**Affiliations:** 1 State Key Laboratory of Cotton Biology, Institute of Cotton Research, Chinese Academy of Agricultural Sciences, Anyang, 455000 Henan China; 2 Xinjiang Production & Construction Group Key Laboratory of Crop Germplasm Enhancement and Gene Resources Utilization, Biotechnology Research Institute of Xinjiang Academy of Agricultural and Reclamation Science, Shehezi, 832000 Xinjiang China; 3 Zhengzhou Research Base, State Key Laboratory of Cotton Biology, Zhengzhou University, Henan , 450001 Zhengzhou China; 4 Development Center for Science and Technology, Ministry of Agriculture and Rural Affairs, Beijing, 100122 China

**Keywords:** *RAD*, *G. hirsutum*, Phylogenetic tree, Phytohormones, Sequence logs, *Cis*-acting elements

## Abstract

**Background:**

RADIALIS (*RAD*), belongs to the MYB gene family and regulates a variety of functions including floral dorsoventral asymmetry in *Antirrhinum majus* and development of fruit proteins in *Solanum lycopersicum*. *RAD* genes contain an SNF2_N superfamily domain. Here, we comprehensively identified 68 *RAD* genes from six different species including *Arabidopsis* and five species of cotton.

**Results:**

Phylogenetic analysis classified *RAD* genes into five groups. Gene structure, protein motifs and conserved amino acid residues indicated that *GhRAD* genes were highly conserved during the evolutionary process. Chromosomal location information showed that *GhRAD* genes were distributed unevenly on different chromosomes. Collinearity and selection pressure analysis indicated *RAD* gene family expansion in *G. hirsutum* and *G. barbadense* with purifying selection pressure. Further, various growth and stress related promotor *cis*-acting elements were observed. Tissue specific expression level indicated that most *GhRAD* genes were highly expressed in roots and flowers (*GhRAD2*, *GhRAD3*, *GhRAD4* and *GhRAD11*). Next, *GhRAD* genes were regulated by phytohormonal stresses (JA, BL and IAA). Moreover, Ghi-miRN1496, Ghi-miR1440, Ghi-miR2111b, Ghi-miR2950a, Ghi-miR390a, Ghi-miR390b and Ghi-miR7495 were the miRNAs targeting most of *GhRAD* genes.

**Conclusions:**

Our study revealed that *RAD* genes are evolutionary conserved and might be involved in different developmental processes and hormonal stress response. Data presented in our study could be used as the basis for future studies of *RAD* genes in cotton.

**Supplementary Information:**

The online version contains supplementary material available at 10.1186/s12864-021-08248-z.

## Background

*MYB* gene family members mainly regulate apoptosis, cell differentiation and proliferation. A- MYB, B- MYB and C-MYB are three groups of MYB gene family [1]. These three groups are largely present in vertebrates, fungi, slime molds and insects [2, 3]. For the very first time, MYB gene C1 was isolated from maize and found to regulate biosynthesis of anthocyanin [4]. MYB proteins play a key role in different developmental processes including trichome differentiation, biosynthesis of anthocyanin and flavonoids, floral symmetry, cell proliferation and control of cell cycles [5, 6]. In *Antirrhinum majus*, two dorsal petals of zygomorphic flowers were larger than ventral and lateral ones with single aborted stamen [7]. RADIALIS (*RAD*) belongs to *MYB* gene family. *RAD* is involved in creating the dorsal identity and regulating the domain activity of DIVIRICATA (*DIV*) (another transcription factors of MYB gene family) by restricting it to ventral regions of the flower [8]. *RAD* and *DIV*, interact with each other and control dorsoventral asymmetry of flowers [9].

In *Arabidopsis, RAD* and *DIV* gene families have 198 paralogs and they largely contribute in determining orthology across taxa. *MYB* genes contained three alpha helices. *RAD* and *DIV* have 64 members in *Arabidopsis* and are further classified into five subgroups. Among five subgroups, *RAD* falls in the I-box-like subgroup while R-R-type subgroups include *DIV*. *RAD* was the result of loss of the second MYB domain of *DIV* [10]. First two helices of *RAD* share more similarity with the N terminal MYB domain of *DIV* as compared to third helix and third helix include DNA binding domain of both *RAD* and *DIV* [11].

*RAD5*, is a member of Snf2 helicase family, encodes two domains including RING-finger domain and HIRAN domain. In yeast (*Saccharomyces cerevisiae*), *RAD5* is the key component in the *RAD5*-dependent error free branch of postreplication repair*. AtRAD5a and AtRAD5b* share significant identity to *RAD5* of yeast. *AtRAD5a* and *AtRAD5b* encodes same domain and these two genes have significant similarities with each other as compared to *RAD5* of yeast. However, both *AtRAD5a* and *AtRAD5b* differ in their function [12]. On the basis of epistatic analysis, yeast DNA repair genes can be classified into three different groups, including *RAD3*, *RAD52* and *RAD6* groups. *RAD3* group control nucleotide excision repair and also eliminate DNA damages caused by ultraviolet radiation [13]. However, the *RAD52* group mainly functions in the repair of double-strand breaks induced by ionizing radiation and *RAD6* group overcomes damages of DNA replication and also arrest replication forks [14]. These DNA repair groups encodes members of the DNA helicase-like-SNF2-gene family [15]. SNF2 family members undergo three main functions including binding of specific proteins to chromatin, destabilization of nucleosome structure and alteration of contact points between DNA and proteins [16, 17].

Cotton is an economic crop of the world and provides raw materials for the textile industry and edible oils [18]. *G. hirsutum* and *G. barbadense* are the two allotetraploid species of cotton and provide fiber, seed oil and protein meal to the industry [19]. *G. hirsutum* is famous for its high yield and moderate fiber qualities, while *G. barbadense* is important for superior quality fibers and accounts for 3% of the world’s cotton production [20]. Although some *RAD* genes have been previously reported in model organisms *Arabidopsis* and yeast, the function of *RAD* gene family in cotton has not been reported. In the present research, we systematically identified *RAD* genes in six different species and analyze their phylogenetic relationship, exon and intron structure, conserved protein motifs, biophysical properties, *cis*-acting elements of promoter regions, sequence logos, chromosomal localization, collinearity and miRNA target sites. Present study will increase our understanding about *RAD* gene family and provide insight into the molecular mechanisms of *RADs* in different *Gossypium* species.

## Results

### Identification of *RAD* genes in *Gossypium* species

In this study we used different bioinformatic analysis to identify *RAD* genes in various plant species. We identified 17 *RAD* genes in *G. hirsutum*, 16 genes in *G. barbadense*, eight genes each in *G. herbaceum* and *G. arboreum* and nine genes in *G. raimondii*. Furthermore, we also identified 10 *RAD* genes in *Arabidopsis.* SMART (http://smart.embl-heidelberg.de/), PROSITE (http://prosite.expasy.org/) and InterProscan 63.0 (http: //www.ebi.ac.uk/interpro/) were used for confirmation of *RAD* genes in different species. We found that among all the selected plant species, *G. hirsutum* had the highest number of *RAD* genes (Table S1) elucidating that during hybridization, *GhRAD* genes underwent polyploidization and experienced significant dupli- cation events. The biophysical properties of the *GhRAD* genes including number of amino acids, chromosomal position, isoelectric point, molecular weight, grand average of hydropathicity and protein localization were observed (Table S2). The number of amino acids of GhRAD proteins range from 885 (*GhRAD1*) to 1374 (*GhRAD13*). The isoelectric point (pI) of *GhRAD* genes range from 5.26 (*GhRAD14*) to (9.04) (*GhRAD8*). Molecular weight ranges from 76561.78 Da (*GhRAD11*) to 151381.67 Da (*GhRAD13*). Moreover, predicted subcellular localization indicated that out of 17 *GhRAD* genes 16 genes were located in the nucleus while only one gene was located in the cytoplasm.

### Phylogenetic analysis, structural features, conserved domain and motifs analysis of *GhRAD* genes

**Fig. 1:**
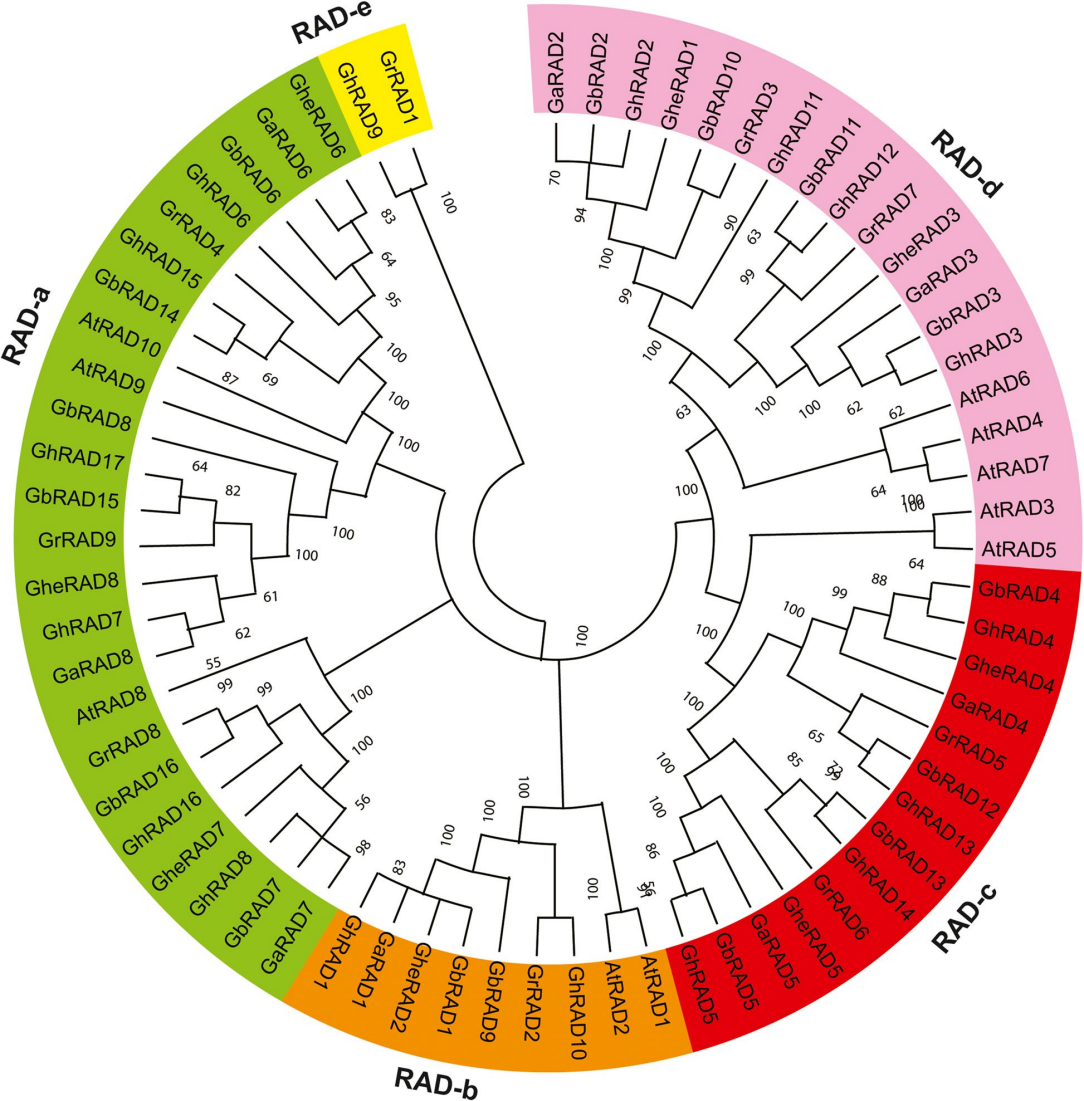
Phylogenetic analysis of RAD proteins in six different species. Prefixes *Gh*, *Gb*, *Ghe*, *Ga*, *Gr* and *At* were used to identify *RAD* genes from *G. hirsutum*, *G. barbadense*, *G. herbaceum*, *G. arboreum*, *G. raimondii* and *Arabidopsis*

To study the phylogenetic relationships of *RAD* gene family, 68 genes from six plant species were used to create a phylogenetic tree (Fig. 1). *RAD* genes were classified into five clades with 24 members in RAD-a clade, nine members in RAD-b, 14 members in RAD-c while RAD-d and RAD-e contained 18 and two members respectively. RAD-a with maximum number of genes contained RAD proteins from all six plant species while RAD-e, the smallest clade contained two genes from *G. hirsutum* and *G. raimondii*. All five clades contained genes from six species except RAD-e and one gene of *Arabidopsis* (*AtRAD6*) did not fall in any clade. Moreover, we created another phylogenetic tree to explore the evolutionary relationship between *G. hirsutum* and *Arabidopsis* (Fig. S1). Phylogenetic tree divided 17 *G. hirsutum* and 10 *Arabidopsis RAD* genes into three clades. Rad-c was the largest clade with 12 members, while Rad-b was the smallest one with four members. *GhRAD9* did not fall in any clade, demonstrating that this genes was dissimilar from each other and it has some special function in species evolution.

Next to study the structural features of *GhRADs*, we analyzed the exon and intron structure, conserved domain and motifs using the GSDS program and MEME tool respectively. Multiple numbers of exons in *GhRAD* genes ranging from 3 to 24 were observed (Fig. S2). Maximum number of exons were present in *GhRAD9* while *GhRAD8* had minimum number of exons. Similarly, *GhRAD9* contained the highest number of introns while *GhRAD16* contained two introns. Number of introns ranged from 2 to 23 for *GhRAD* genes. *GhRAD11* had the longest genomic sequence of more than 8 kb while *GhRAD16* had the shortest genomic sequence of 4 kb. Next, we analyzed the conserved domain of *GhRAD* proteins. Results of conserved domain analysis showed that all *GhRAD* proteins contain SNF2_N superfamily domain (Fig. S3). Furthermore, we analyzed the motif distribution pattern of *GhRADs* and found that ten conserved motifs were identified in *GhRAD* proteins (Fig. S4). Results indicated that *GhRAD* genes had conserved gene structure and protein motif distribution pattern although the process of gene duplication took place a long time ago during evolution.

### Chromosomal position information and synteny analysis

Chromosomal position of *GhRADs* exhibited that 17 *GhRAD*s were distributed unevenly to 11 chromosomes. Out of 17 genes, six genes were mapped on At sub-genome, while nine *GhRAD* genes were mapped on Dt subgenome and two genes were present in the form of scaffolds (Fig. S5). This uneven distribution pattern of *GhRAD* genes on chromosomes illustrated the presence of genetic variability during the evolutionary process. More deeper insights showed that the maximum number of genes were mapped on chromosome D02. Most chromosomes contained a single *GhRAD* gene and no gene was located on chromosome A02 and A04 of At sub-genome as well as chromosome D03 of Dt sub-genome.

**Fig. 2:**
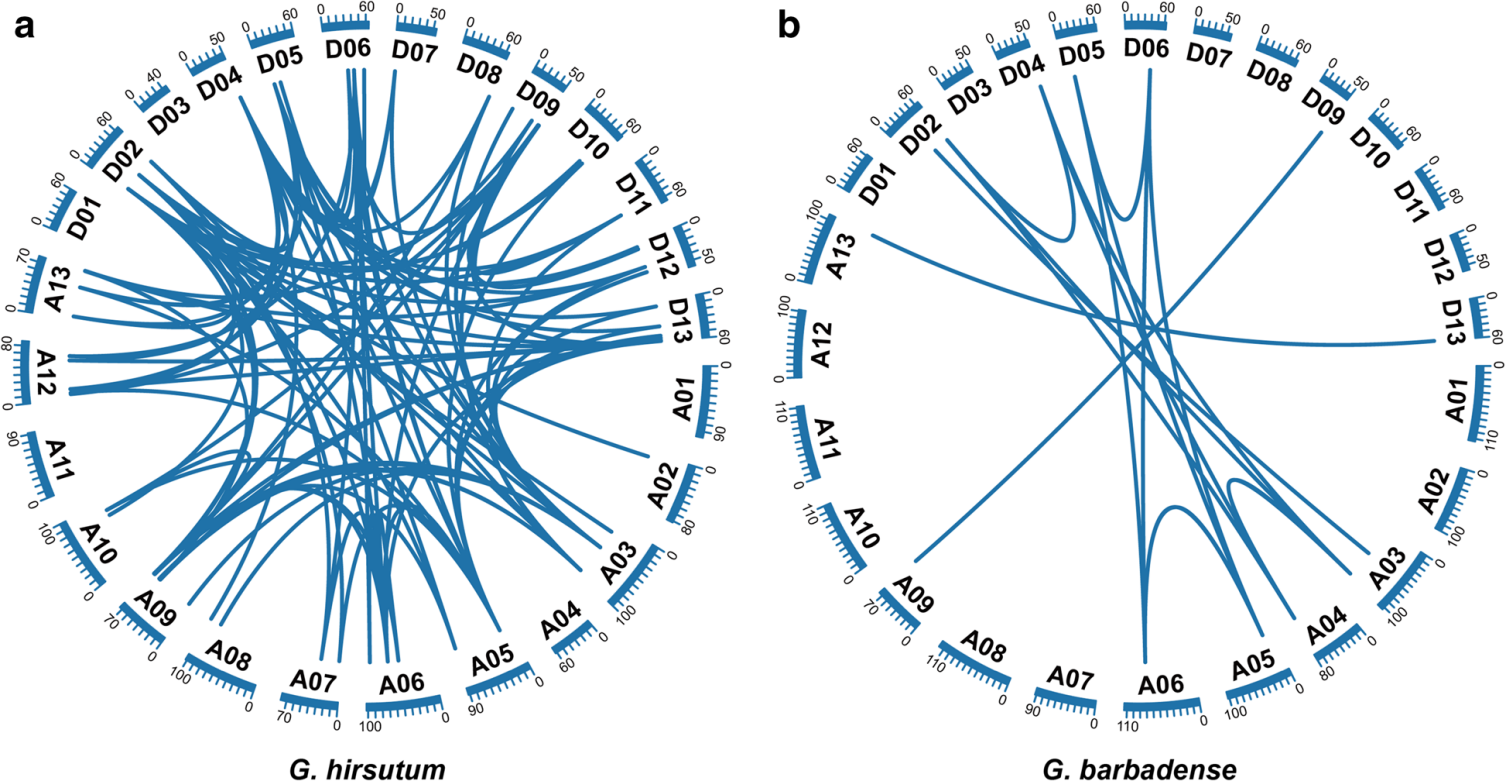
Collinearity analysis of *GhRAD and GbRAD* genes. Orthologous/paralogous gene pairs were indicated by blue color lines

Collinearity analysis between orthologs of At and Dt sub genomes revealed that the different *GhRAD* and *GbRAD* loci showed significantly conserved pattern among At and Dt sub-genomes (Fig. 2 A and B and Table S3, S4). Our results indicated that At and Dt sub-genomes are orthologous in the A genome (*G. arboreum*) or D genome (*G. raimondii*). Upland cotton (*G. hirsutum*) was produced after hybridization between *G. arboreum* and *G. raimondii*. In our study, 112 and 15 orthologous/paralogous gene pairs were identified in *G. hirsutum* and *G. barbadense* respectively*.*

*Ka/Ks* ratios of *GhRAD* and *GbRAD* genes were calculated by *Ka/Ks* calculator 2.0 (Table S3, S4). In the course of evolution, duplicated gene pairs underwent non-functionalization, neofunctionalization and sub-functionalization [21]. In *G. hirsutum* 74 gene pairs exhibited *Ka/Ks* values below 0.5, illustrating that *GhRAD* genes bear purifying selection pressure while 15 genes showed *Ka/Ks* ratio above 0.5 and only three genes have *Ka/Ks* ratio of less than 0.1. While in *G. barbadense* 15 genes exhibited *Ka/Ks* ratio of less than 0.5. Taken together, our results indicated that purification selection pressure has a great contribution in maintaining the functional divergence of *GhRAD* and *GbRAD* genes.

### Sequence logos and *cis*-acting elements

**Fig. 3:**
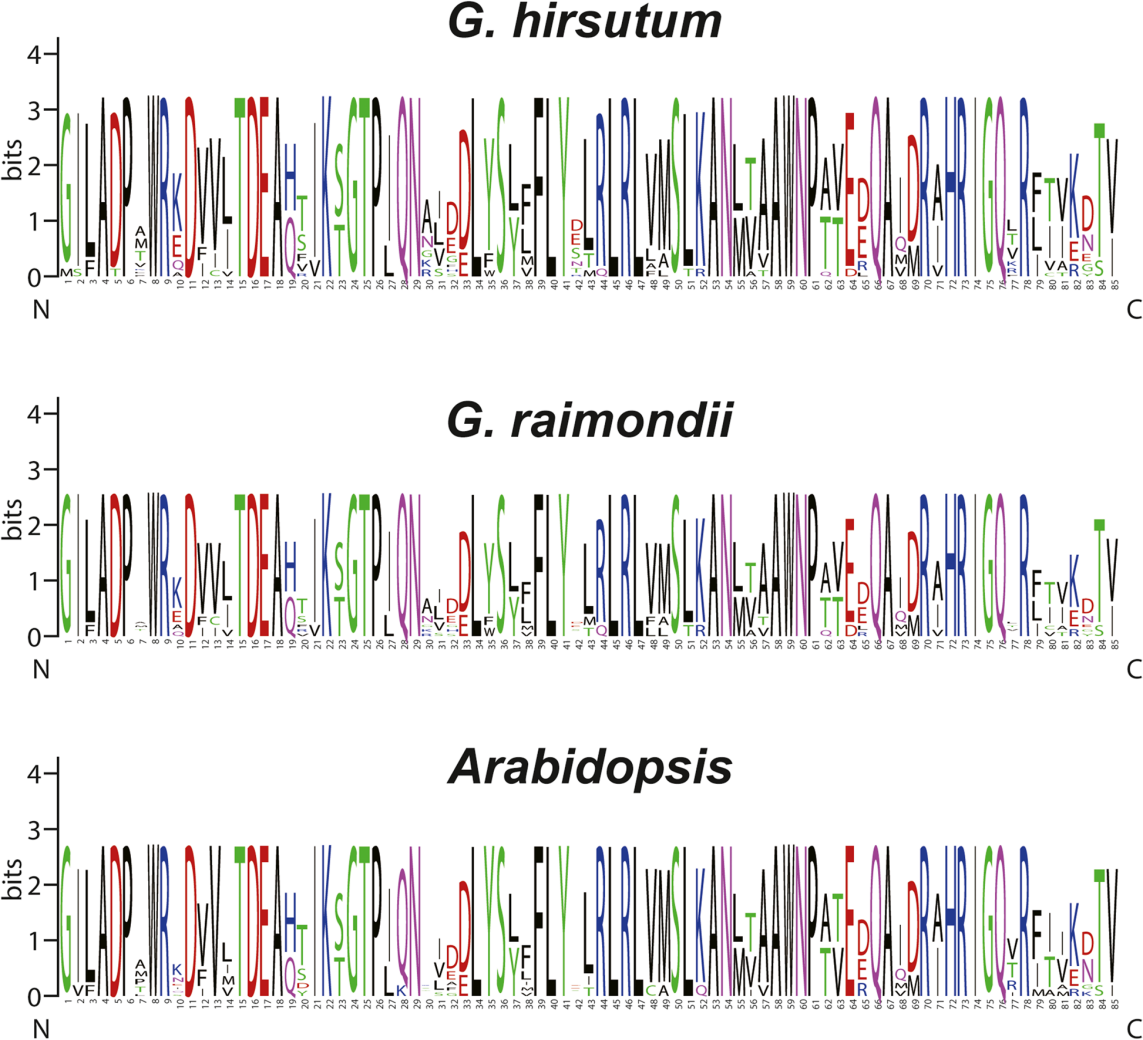
Sequence logos for conserved amino acid residues in *G. hirsutum*, *G. raimondii* and *Arabidopsis.* Protein sequence logos were created using online software WEBLOG

We created the protein sequence logos of *Arabidopsis, G. hirsutum* and *G. raimondii* to reveal the conservation of *RAD* genes during the process of evolution. Sequence logos provide valuable information about sequence similarities of selected plant species. Results indicated that protein sequence logos were conserved among all observed plant species at most sites within and between species across the N and C terminals (Fig. 3).

**Fig. 4:**
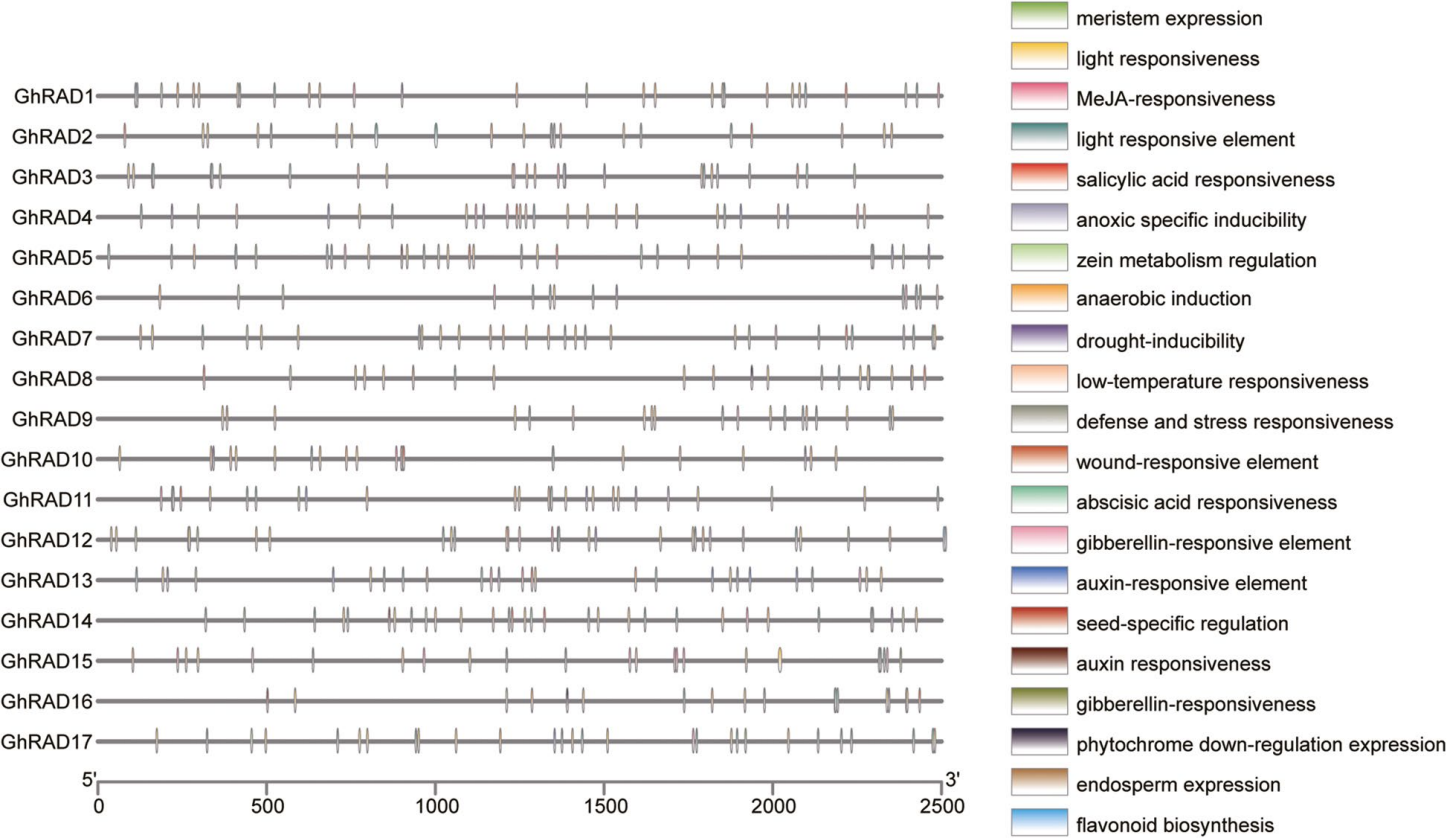
Analysis of promoter *cis*-acting elements of *GhRAD* genes. *Cis*-acting elements were confirmed using PlantCARE database. *Cis*-acting elements were represented by different colour boxes

Next, we identified different *cis*-acting elements of *GhRAD* genes. Process of gene expression and transcription can be well studied by analyzing the promotor *cis*-acting elements present in specific gene. The 17 putative *GhRAD* promoter regions possessed typical core *cis*-acting elements that were related to different phytohormones like MeJA, gibberellin, auxin, salicylic acid and abscisic acid (Fig. 4). Most of the *GhRAD* genes promoter shared numerous elements for plant growth and development such as seed specific regulation, meristem and endosperm expression and cell cycle regulation. Additionally, we found that many drought responsive elements, flavonoid biosynthesis, stress responsive elements, light and low temperature responsive elements, anaerobic induction, anoxic specific inducibility and wound responsive elements were also present in *GhRADs* promotor regions.

### Expression profile of *GhRAD* genes in different tissues and hormonal stresses

**Fig. 5:**
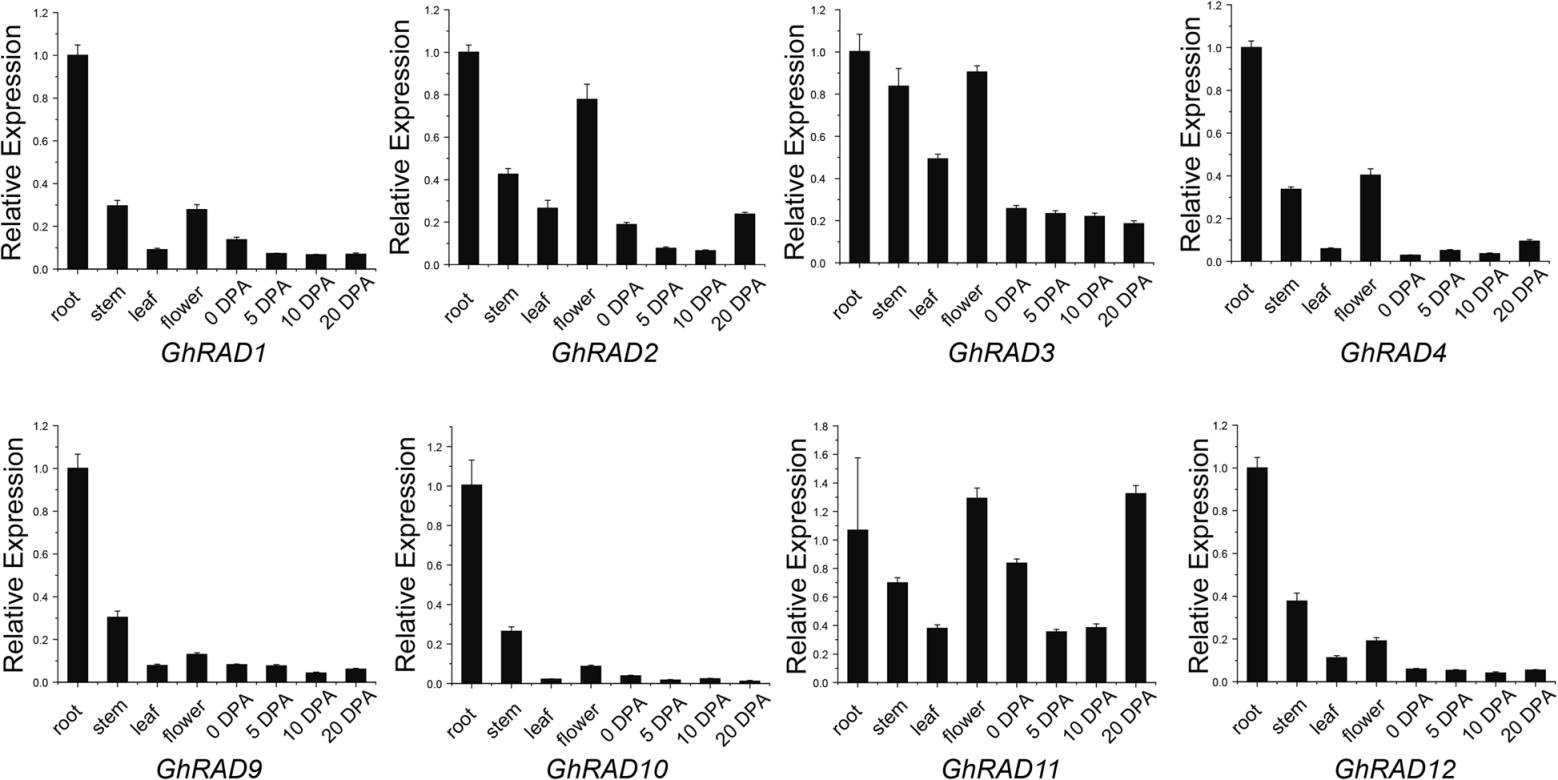
Tissue specific expression patterns analysis of *GhRAD* genes. Tissues were collected at different development stages such as root, stem, leaf, flower and 0, 5, 10 and 20 DPA fiber

Analysis of gene expression patterns is helpful to predict the biological functions of genes. We downloaded the RNA-seq data from NCBI. We investigated the gene expression pattern of *GhRADs* in various tissue samples and a heatmap was generated (Fig. S6). As shown in figure, *GhRAD1* and *GhRAD10* showed high transcript level in almost all selected tissues demonstrating that these two genes have multiple biological functions and they have some special functions related to fiber development while *GhRAD6* and *GhRAD15* were poorly expressed in some tissues showing that these two genes might be pseudo genes and have limited role in cotton growth and development. Next to validate the results of RNA-seq data, we performed qRT-PCR analysis in eight different tissues including root, leaf, stem, flower and 0, 5, 10 and 20 DPA fiber (Fig. 5). Results indicated that most of the *GhRAD* genes showed preferential expression in root except *GhRAD11* whose expression was preferentially high in 20 DPA fiber, indicating its potential role in fiber elongation and biosynthesis of secondary cell wall. *GhRAD2*, *GhRAD3*, *GhRAD4* and *GhRAD11* showed high expression in flower tissues demonstrating that these *RAD* genes might function in reproductive development.

**Fig. 6:**
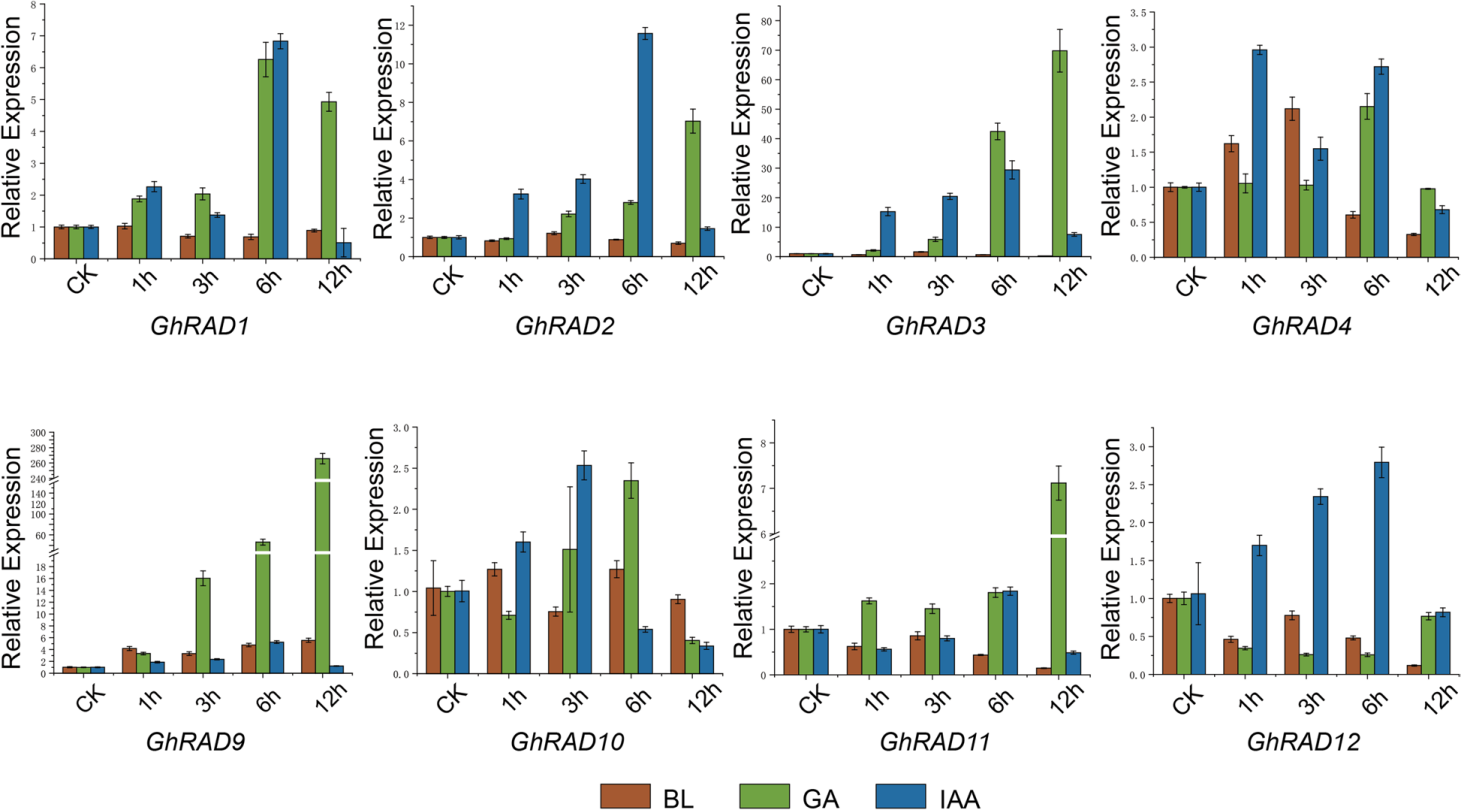
Expression pattern analysis of *GhRAD* genes under different phytohormonal stresses including BL, GA and IAA at 1, 3, 6 and 12 h of treatment by qRT-PCR analysis

Next, we inspected the transcript level of *GhRADs* under three phytohormonal treatments (BL, JA and IAA) at 1, 3, 6 and 12 h of treatment (Fig. 6). Results revealed that most of the *GhRAD* genes showed up and downregulated expression patterns in different hormones with different time points. *GhRAD1*, *GhRAD2* and *GhRAD9* showed upregulated expression in all three selected phytohormonal stresses, illustrating that these genes might respond to various hormone signaling pathways. *GhRAD1* showed significantly upregulated expression at 1, 3, 6 and 12 h of GA treatment, indicating its potential role in GA signaling pathway. Moreover, transcript level of *GhRAD12* was preferentially high at 1, 3 and 6 h of IAA treatment, suggesting that *GhRAD12* might respond positively to IAA signal. Taken together our results illustrated that *GhRAD* genes were important in several phytohormone signaling pathways.

### Prediction of miRNA target sites

**Fig. 7:**
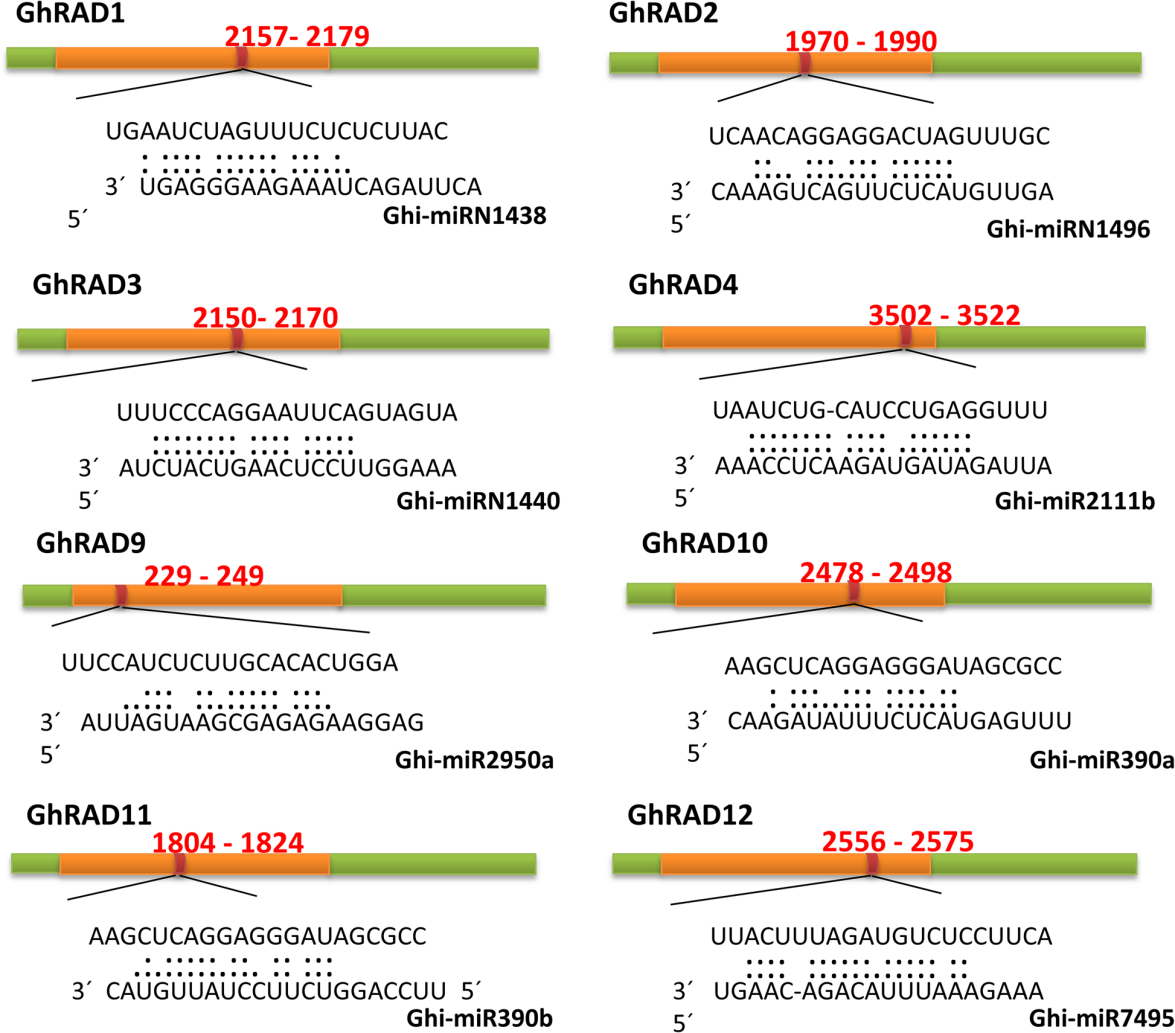
The miRNA-mediated target sites of *GhRAD* genes. Green color boxes indicated ORFs (open reading frames) of *GhRADs*. SNF2_N superfamily domain was represented by orange box while miRNA complementary sites with the nucleotide positions of *RAD* genes were indicated by red color

In plants, microRNAs (miRNAs) help in regulation of gene expression by activating mRNA translational repression [22]. To explore miRNA mediated post-transcriptional regulation of *GhRAD* genes, coding sequences of 17 *GhRAD* genes for putative target sites were searched with the help of psRNATarget server. Results indicated that *GhRAD1* was targeted by Ghi-miRN1438 with a site in SNF2_N superfamily domain (Fig. 7 and Table S5). Similarly, *GhRAD2*, *GhRAD3* and *GhRAD4* were targeted by Ghi-miRN1496, Ghi-miR1440 and Ghi-miR2111b respectively. Further, Ghi-miR2950a, Ghi-miR390a, Ghi-miR390b and Ghi-miR7495 regulated *GhRAD9*, *GhRAD10*, *GhRAD11* and *GhRAD12* respectively, with SNF2_N superfamily domain. These results suggested that *GhRAD* genes can be regulated by different miRNAs.

## Discussion

Cotton is an important economic crop of the world and contributes largely to the textile industry. Different gene families such as *GGPPS* [23], *LOG* [24], *BES1* [25], *GH3* [26], *GSK* [27], *GATL* [28], *GhPHD* [29] and *GhAA1* [30] have been identified in cotton, but there is no published research about *RAD* gene family in cotton. Previous studies reported that *RAD51* participate in DNA repair, homologous recombination and genome stability. Members of the *recA*_*RAD51* family perform functions that have differentiated during evolution [31]. Human *RAD51* is a homologue of the *Escherichia coli* RecA protein and function in recombinational repair of double-stranded DNA breaks. Mutations of *RAD51* reduce repair of double-stranded DNA breaks. Loss of *RAD51* function result in an elevated mutation rate and accumulation of DNA damage therefore increase risk of cancer in human [32]. In *Antirrhinum, RADIALIS* gene is associated with regulation of floral asymmetry [33]. Induction of recessive mutation in *RADIALIS* gene produce symmetrical floral morphology. The *Arabidopsis RAD* gene family contains four members, including *RSM1*, *RSM2, RSM3 and RSM4, RADIALIS-LIKE SANT/MYB 1–4*). *Arabidopsis RAD*-like (*RSM*) genes might function in developmental process that is not necessarily relevant to the floral architecture [34] [35].

*Gossypium* genus contain 45 diploid and six tetraploid species [36, 37]. Two allotetraploid species including, *G. hirsutum* and *G. barbadense* arisen through interspecific hybridization of A and D genome progenitors [19]. Polyploidy occurred around 1–2 million years ago and produced allotetraploid species [38]. *G. hirsutum* and *G. barbadense* are the two oldest allotetraploid species of cotton [39] [40]. Here, we comprehensively identified *RAD* genes in different plant species to identify the diverse functions of *RAD* gene family during the process of plant growth and development. Our study will provide basic information about biological and functional mechanisms and could be helpful for further research of *RAD* gene family in different cotton species.

### *GhRAD* genes show evolutionary conservation

In this study, we identified *RAD* genes from *Arabidopsis* and five *Gossypium* species*.* Interestingly, the number of *GhRAD* genes was higher as compared to *GaRAD* and *GrRAD* genes, possibly because *G. hirsutum* is an allotetraploid crop resulting from hybridization of A and D genome progenitors. In upland cotton, both At and Dt sub-genome donors are orthologous relatives, leading to the *GhRAD* gene duplication [41]. Because of duplication and doubling of *GhRAD* genes, the numbers of *GhRAD* genes were equal to the total number of *GaRAD* and *GrRAD* genes. Phylogenetic analysis naturally classified *RAD* genes into five clades from RAD-a to RAD-e with 24 genes in RAD-a clade and two genes in RAD-e clade. Most of the *RAD* genes from diploids and allotetraploids were distributed closely in the phylogenetic tree, indicating that tetraploid cotton was produced after the hybridization of A and D genomes progenitors. From the phylogenetic tree we observed that RAD-a, RAD-b and RAD-d clades were ancient groups containing *RAD* genes from all selected plant species. Whereas, RAD-e clade might be more advanced containing *RAD* genes from only two selected plant species including *G. hirsutum* and *G. raimondii*, however, RAD-c clade lacks the genes from *Arabidopsis.*

Analysis of sequence logos of *G. hirsutum, G. raimondii* and *Arabidopsis* also supported these findings as the results of sequence logos showed significantly conserved pattern of amino acid across N and C terminals. Each conserved amino acid showed similar positions in three different species.

Interaction between transcription factors and promoter binding sites is important for gene regulation at the transcriptional level and transcriptional regulation is mainly responsible for regulation of gene expression. Different external signals activated inducible promoters and *cis*-acting elements in promoter regions are specific and consistent, for example, *cis*-acting elements of AuxRE were generally present in auxin-induced promoters regions [42], similarly AT-rich *cis*-acting elements, I-box, G-box, and GT1-motif were generally present in the light-induced promoters [43–45]. Likewise, CACG and CATGTG *cis*-acting elements were found in drought-induced promoters [46]. In our study, we extracted the upstream promoter fragments of candidate genes. Phytohormone (IAA, SA, ABA, GA and MeJA) responsive *cis*-acting elements were present in *GhRAD* genes promotors. Previous studies have shown that phytohormones maintain the integrity of plant cell walls. MeJA mediates gene expression in responses to plant injury [47]. Auxin regulates cell walls by induction of cell wall looseness [48]. In strawberry, ethylene promotes fruit softening and ripening by regulating the synthesis of pectin [49]. Stress responsive *cis*-acting elements help plants to respond quickly to stress and improve plant resistance to environmental stresses by activating different stress related genes. *GhRAD* promoter regions contain different *cis*-acting elements related to phyto- hormones, plant growth and development, seed specific regulation, meristem and endosperm expression and cell cycle regulation. In the present study, we found the uneven distribution of *GhRADs* on the chromosomes of At and Dt sub-genome. Uneven distribution of *GhRAD* genes indicated that during evolution and hybridization, these genes might have experienced gene duplication.

Gene structure is important to predict gene evolution [50]. Here, we found that the number of exons ranged from 3 to 24 while the number of introns ranged from 2 to 23. We observed that length of intron is significantly different, suggesting that during functional diversification of *GhRADs* intron length might play major roles. It has been reported that gene structure is associated with the evolution of different plant species [51]. The evolutionary analysis within the *RAD* genes showed that most of the *RAD* genes were greatly conserved during evolution. As *RAD* genes introns were not lost during evolution and at the early expansion stages of evolution, these genes diverged, whereas, over evolutionary time, other genes lost their introns [52], indicating that group specific genes may have similar functions. Insertion/deletion events might be the reason for structural differences of exon/intron and may help to identify different evolutionary mechanisms [53]. Gene families with fewer number of introns are considered to be advanced gene families [54] while gene families showing more introns have acquired some novel functions during the evolutionary process. Furthermore, motif distribution pattern of *GhRAD* genes was conserved indicating that the proteins with similar kind of motif distribution pattern might have some specific functions in cotton growth and development.

### Diverse expression patterns of *GhRADs*, prediction of their miRNA target sites and selection pressure

In the recent years, different researches have been done to identify the function of *RAD* genes in different plant species but no research has been conducted to find the functional mechanisms of *RAD* genes in cotton. As our results indicated that promotor of *GhRADs* contain elements related to phytohormones, plant growth and development, seed specific regulation, meristem and endosperm expression and cell cycle regulation, so to check the potential regulatory functions of *GhRADs* during cotton growth and development, we evaluated the transcript patterns of 17 *GhRAD* genes using qRT-PCR. Results indicated that many *GhRAD* genes showed high expression in roots. However, *GhRAD2*, *GhRAD3*, *GhRAD4* and *GhRAD11* showed significantly high expression in flower tissues. Results indicated that *GhRAD* genes might be playing a positive role in root and flower development. All *GhRAD* genes showed poor expression in fiber development stages except *GhRAD11* had relatively high expression at 20 DPA fiber.

Different research studies indicated that phytohormones respond positively to abiotic stress response [55]. Auxin response factors (ARFs) activate auxin responsive genes and ARFs are the potential mediators that help the plants to respond to adverse environmental conditions [56]. Ethylene response factors of AP2/ARF transcription factor family play a positive function in plant growth, disease resistance and phytohormone response [57]. In this study, analysis of *GhRADs* for three different phytohormones indicated that *GhRAD* genes can be regulated by the exposure of phytohormones such as BL, IAA and JA. *GhRAD1* and *GhRAD2* showed upregulated expression for observed phytohormones, suggesting that these genes may have a positive role in phytohormones signaling.

Previous studies reported that miRNAs have significant functions in plant growth and development, regulation of cell growth and different metabolism associated with cotton fiber development and also respond to different environmental stresses including cold, heat and drought [58]. In the past few years, different miRNAs have been identified in cotton and they are preferentially expressed in different vegetative tissues as well as during abiotic stresses [59, 60]. For instance, 32 families of miRNA were found to be differentially expressed in cotton ovules and leaves. With the help of RNA-seq data of cotton leaf and ovule, a total of 65 families of cotton miRNA have been identified [61]. In our study we identified different *GhRAD* genes targeted by various miRNA. Precisely, Ghi-miRN1496, Ghi-miR1440, Ghi-miR2111b, Ghi-miR2950a, Ghi-miR390a, Ghi-miR390b and Ghi-miR7495 were the miRNAs targeting most of *GhRAD* genes. Our results will lay the foundation for future research about biological functions of *GhRAD* miRNAs and also their target sites in *G. hirsutum*.

Next we calculated the *Ka/Ks* ratios of *GhRADs* and *GbRADs.* Genes with *Ka/Ks* = 1.0 are considered as pseudogenes as a result of neutral selection, *Ka/Ks* less than1.0 demonstrates the tendency of duplicated genes for purifying selection, while *Ka/Ks* ratios greater than 1.0 exhibits positive selection of accelerated evolution. *Ka/Ks* values of most *GhRAD* and *GbRAD* were less than 1 indicating that during the long-term evolutionary process, *RAD* gene family experienced purifying selection pressure.

## Conclusions

In the present study, we conducted a comprehensive genome wide analysis to identify phylogenetic relationship, sequence conservation and biological functions of *RAD* genes. Phylogenetic analysis divided *RAD* genes into five clades on the basis of sequence homology. Sequence logos, exon-intron structure, protein motifs and conserved domain analysis indicated that *GhRAD* genes were highly conserved during evolution with the uneven distribution on the chromosomes. Collinearity and selection pressure analysis indicated the expansion of *RAD* gene family in *G. hirsutum* and *G. barbadense* with purifying selection pressure. Promoter *cis*-acting elements analysis indicated the presence of several plant growth and stress related regulatory elements. Tissue specific expression pattern analysis of *GhRAD* genes indicated that some *GhRAD* genes showed significantly high expression in roots and flowers, except *GhRAD11* had maximum expression at 20 DPA fiber. Further, *GhRADs* showed regulatory response under three phytohormonal stresses indicating candidate genetic material for cotton breeding against different stresses.

## Methods

### Identification of *RAD* gene family

The database of cotton species including (*G. hirsutum, G. raimondii, G. arboreum, G. barbadense* and *G. herbaceum*) were acquired from the Cotton FGD (https://cottonfgd.org/) [62]. The *Arabidopsis* protein sequences of *RAD* genes were acquired from *Arabidopsis* Information Resource, version 10 (TAIR 10) (http://www.arabidopsis.org). The *AtRAD* genes were used as queries to identify the *RAD* genes in other plant species. SMART (http://smart.embl-heidelberg.de/) and Interproscan 63.0 (http://www.ebi.ac.uk/InterProScan/) were used to confirm *RAD* protein sequences [63, 64]. Furthermore, Pfam: (http://pfam.janelia.org/) and hidden Markov model (HMM) was used to identify the conserved domain of *RADs* [65]. Moreover, to predict the biophysical properties of *RAD* genes, ExPASy-ProtParam tool (http://us.expasy.org/tools/protparam.html) was used. Subcellular localization was predicted by CELLO v2.5 server [66].

### Phylogenetic analysis and sequence logos

For phylogenetic analysis of *RAD* gene family, Clustal W was used for multiple sequence alignment [67] with the default settings and the phylogenetic tree was generated by iTOL [68] with 1000 bootstrap replicates. For sequence logos, RAD proteins of *Arabidopsis, G. hirsutum* and *G. raimondii* were aligned with Clustal W [69] and protein sequence logos were created using online software WEBLOG with default parameters [70].

### Chromosomal location, gene structure and analysis of protein motif

Chromosomal position of *GhRADs* was determined by cotton genome annotation data (ftp://ftp.bioinfo.wsu.edu/species/Gossypium hirsutum/NAU-NBI_G) and extracted gff3-files was used in MapInspect software (https://mapinspect.software.informer.com/) to map genes to their corresponding chromosomes. For gene structure analysis of *GhRADs*, Gene Structure Display Server 2.0 (GSDS) was used (http://gsds.cbi.pku.edu.cn/index.php) [71]. Online program MEME (http://meme-suite.org) [72] was used to determined conserved motifs of *GhRAD* gene family and finally motifs were displayed by TBtools [73].

### Promoter *cis*-acting elements, synteny, *Ka/Ks* ratio and transcriptome data analysis

For analysis of promotor *cis-*acting element, 2-kb upstream promotor regions were downloaded from Cotton FGD website [62] and subjected to Plant CARE Database [74]. During collinearity, analysis orthologous/paralogous data were obtained by the previously described methods [75, 76] and circos was used to generate the figure [77]. Non-synonymous (Ka) and synonymous (Ks) divergence level ratios were calculated by aligning duplicated gene pair protein sequences in Clustal X 2.0, after which they were translated into complementary DNA (cDNA) sequences using the PAL2NAL program (http://www.bork.embl.de/pal2nal/). Finally, *Ka* and *Ks* values were calculated with the help of the CODEML program using the PAML package. For expression pattern analysis of *GhRADs*, RNA seq data (https://www.ncbi.nlm.nih.gov/pmc/articles/PMC4482290/) was used and heatmap was generated by using TBtools [73].

### Prediction of miRNA target sites of *GhRAD* genes

Plant MicroRNA database (http://bioinformatics.cau.edu.cn/PMRD/) [78], miRBase (http:// www.mirbase.org/) [79] and the Cotton EST database (http://www.ncbi.nlm.nih.gov/nucest) were used to obtain MicroRNA sequences of *G. hirsutum.* Predictions of miRNA target sites were done with the help of psRNATarget server (http://plantgrn.noble.org/psRNATarget/home) as described previously [67].

### RNA extraction and RT-qPCR analysis

In this research, the CRI24 variety was used for gene expression analysis. Total RNA was extracted by collecting cotton samples from the field. For tissue specific expression pattern analysis, plant samples such as root, stem, leaf, flower and 0, 5, 1 and 20 DPA fiber were used. Similarly, for hormonal stresses, cotton seedlings were subjected to JA, BL and IAA treatment for 1, 3, 6 and 12 h. RNA extraction was done with the help of RNAprep Pure Plant Kit (Tiangen, Beijing, China), after that cDNA was synthesized by using the Prime-Script®RT reagent kit (Takara, Dalian, China). Green qPCR SuperMix was used to conduct qRT-PCR assay and *GhHis3* (GenBank accession no. AF024716) was used as internal control. PCR reaction was done in three replications. Results of qRT-PCR were calculated as described previously [80]. Primers used for RT-qPCR analysis were presented in (Table S6).

## Supplementary Information

**Additional file 1 **: **Figure S1.** Phylogenetic relationship between *G. hirsutum* and *Arabidopsis.* Phylogenetic tree was constructed using RAD protein sequences by iTOL software.

**Additional file 2 **: **Figure S2**. Gene structure of *GhRAD* genes. Yellow boxes represent exons while black lines indicate introns.

**Additional file 3 **: **Figure S3.** Conserved domain analysis of GhRAD proteins. Each domain was represented in different colors and their names were mentioned with color boxes.

**Additional file 4 : Figure S4.** Protein motif analysis of GhRAD proteins. Each motif was indicated with different color and phylogenetic analysis grouped GhRAD proteins according to the protein motif distribution pattern.

**Additional file 5 **: **Figure S5.** Chromosomal distribution of *GhRAD* genes*.* MapInspect software (https://mapinspect.software.Informer.com/) was used to map genes to their corresponding chromosomes.

**Additional file 6 **: **Figure S6**. RNA-seq data analysis of 17 *GhRAD* genes in root, stem, leaf, torus, petal, stamen, pistil, calycle, − 3, − 1, 0, 1, 3, 5, 10, 20, 25 and 35 DPA ovules and 5, 10, 20 and 25 DPA fiber.

**Additional file 7 **: **Table S1.** *RAD5/RAD16*-like Gene Family members.

**Additional file 8 **: **Table S2.** Biophysical properties of the *GhRAD* genes.

**Additional file 9 **: **Table S3**. Orthologous.paralogous gene pairs and *Ks/Ks* values of *GhRAD* gene family members.

**Additional file 10 **: **Table S4.** Orthologous.paralogous gene pairs and *Ks/Ks* values of *GbRAD* gene family members.

**Additional file 11 **: **Table S5**. MicroRNA (miRNA) target sites of *GhRAD* genes.

**Additional file 12 **: **Table S6.** *GhRAD* genes primers for qRT-PCR.

**Additional file 13 **: **Table S7.** Proposed name of *RAD* gene family members.

## Data Availability

All data used in this study are included in this article and additional files. Transcriptome data used for gene expression analysis could be downloaded from (https://www.ncbi.nlm.nih.gov/pmc/articles/PMC4482290/). The Genome sequence and annotation datasets that supported our findings are available in: *A. thaliana*: (http://www.arabidopsis.org), COTTON: (https://www.cottongen.org/) and Other species: (https://jgi.doe.gov/data-and-tools/phytozome/)**.** All the genes used in this study for phylogeny and subsequent analysis are mentioned in additional file 13, table S7 and can be downloaded from Cotton FGD (https://cottonfgd.org/).

## Abbreviations

RAD: RADIALIS
BL: Brassinolide
GA: Gibberellic acid
IAA: Indole − 3-acetic acid
3D: 3 Dimension
DPA: Days post anthesis
