# Correction to: RAD gene family analysis in cotton provides some key genes for fowering and stress tolerance in upland cotton *G. hirsutum*

**DOI:** 10.1186/s12864-022-08500-0

**Published:** 2022-04-04

**Authors:** Nosheen Kabir, Xin Zhang, Le Liu, Ghulam Qanmber, Lian Zhang, Yu Xuan Wang, Zhuojing Sun, Na Zhao, Gang Wang

**Affiliations:** 1 State Key Laboratory of Cotton Biology, Institute of Cotton Research, Chinese Academy of Agricultural Sciences, Anyang, 455000 Henan China; 2 Xinjiang Production & Construction Group Key Laboratory of Crop Germplasm Enhancement and Gene Resources Utilization, Biotechnology Research Institute of Xinjiang Academy of Agricultural and Reclamation Science, Shehezi, Xinjiang, 832000 China; 3 Zhengzhou Research Base, State Key Laboratory of Cotton Biology, Zhengzhou University, Henan, Zhengzhou, 450001 China; 4 Development Center for Science and Technology, Ministry of Agriculture and Rural Affairs, Beijing, 100122 China

**Correction to: BMC Genomics 23, 40 (2022)**

**https://doi.org/10.1186/s12864-021-08248-z**

Following publication of the original article [1], it was reported that affiliations listed for affiliations 2 and 3 were reversed. The correct affiliations are given in this Correction article and the original article [1] has been updated.
