# Supplementary material for: RAD gene family analysis in cotton provides some key genes for flowering and stress tolerance in upland cotton G. hirsutum
Source: BMC Genomics. 2022 Jan 10;23:40. doi: 10.1186/s12864-021-08248-z (PMC8744286; doi:10.1186/s12864-021-08248-z)
Supplement: Supplementary file 2 — Additional file 2 : Figure S2. Gene structure of GhRAD genes. Yellow boxes represent exons while black lines indicate introns. [file 12864_2021_8248_MOESM2_ESM.pdf]

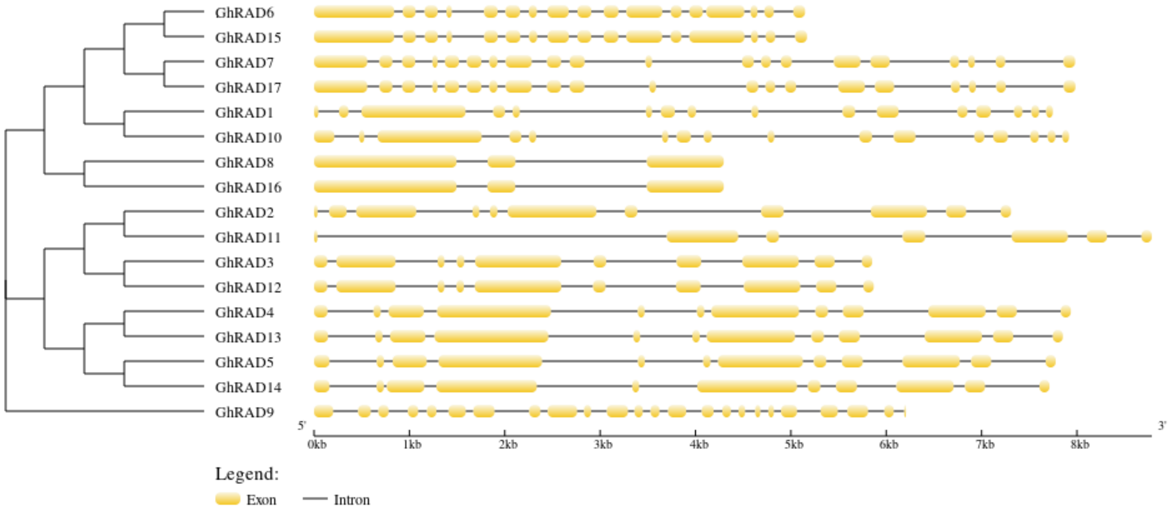

Additional file 2: Figure S2. Gene structure of *GhRAD* genes. Yellow boxes represent exons while black lines indicate introns.
