# Supplementary material for: RAD gene family analysis in cotton provides some key genes for flowering and stress tolerance in upland cotton G. hirsutum
Source: BMC Genomics. 2022 Jan 10;23:40. doi: 10.1186/s12864-021-08248-z (PMC8744286; doi:10.1186/s12864-021-08248-z)

Additional file 3: Figure S3. Conserved domain analysis of GhRAD proteins. Each domain was represented in different colors and their names were mentioned with color boxes.

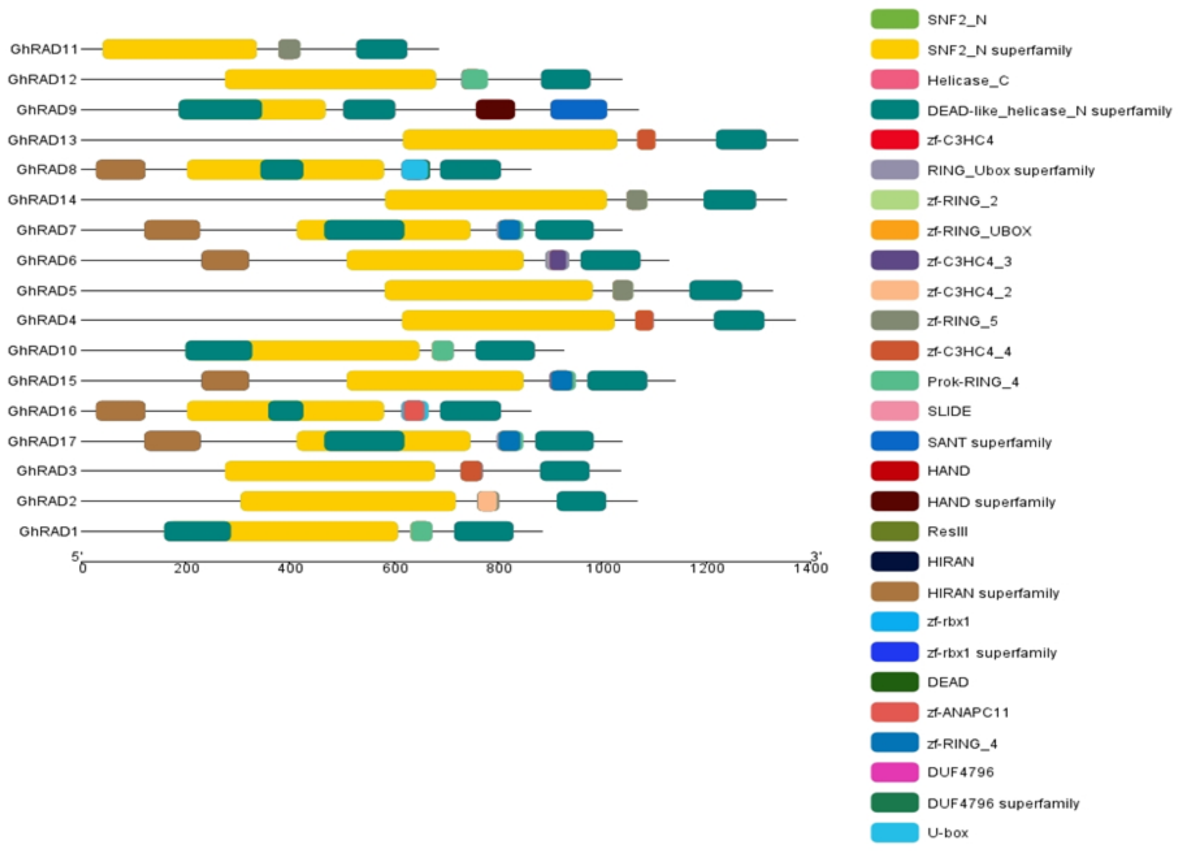

Supplement: Supplementary file 3 — Additional file 3 : Figure S3. Conserved domain analysis of GhRAD proteins. Each domain was represented in different colors and their names were mentioned with color boxes. [file 12864_2021_8248_MOESM3_ESM.pdf]
