# Supplementary material for: RAD gene family analysis in cotton provides some key genes for flowering and stress tolerance in upland cotton G. hirsutum
Source: BMC Genomics. 2022 Jan 10;23:40. doi: 10.1186/s12864-021-08248-z (PMC8744286; doi:10.1186/s12864-021-08248-z)
Supplement: Supplementary file 4 — Additional file 4 : Figure S4. Protein motif analysis of GhRAD proteins. Each motif was indicated with different color and phylogenetic analysis grouped GhRAD proteins according to the protein motif distribution pattern. [file 12864_2021_8248_MOESM4_ESM.pdf]

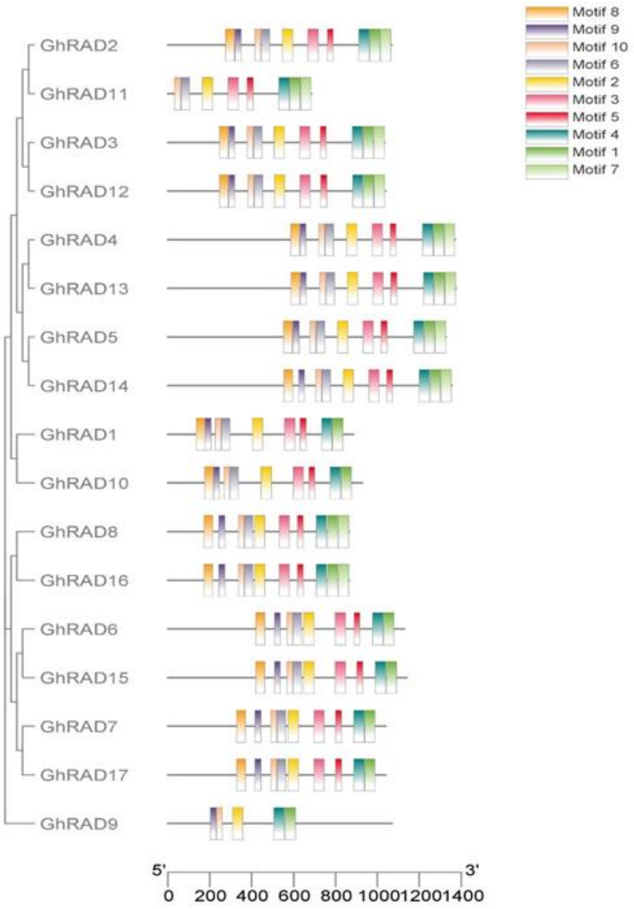

Additional file 4: Figure S4. Protein motif analysis of GhRAD proteins. Each motif was indicated with different color and phylogenetic analysis grouped GhRAD proteins according to the protein motif distribution pattern.
