# Supplementary material for: RAD gene family analysis in cotton provides some key genes for flowering and stress tolerance in upland cotton G. hirsutum
Source: BMC Genomics. 2022 Jan 10;23:40. doi: 10.1186/s12864-021-08248-z (PMC8744286; doi:10.1186/s12864-021-08248-z)
Supplement: Supplementary file 5 — Additional file 5 : Figure S5. Chromosomal distribution of GhRAD genes. MapInspect software (https://mapinspect.software.Informer.com/) was used to map genes to their corresponding chromosomes. [file 12864_2021_8248_MOESM5_ESM.pdf]

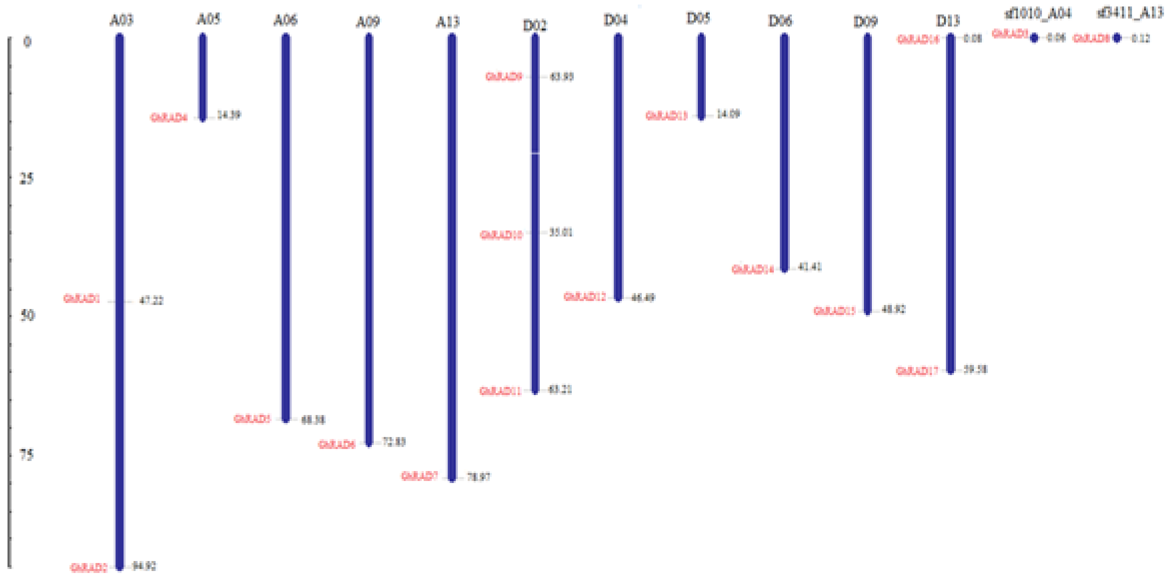

Additional file 5: Figure S5. Chromosomal distribution of *GhRAD* genes. MapInspect software (https://mapinspect. software. Informe r.com/) was used to map genes to their corresponding chromosomes.
