# Supplementary material for: RAD gene family analysis in cotton provides some key genes for flowering and stress tolerance in upland cotton G. hirsutum
Source: BMC Genomics. 2022 Jan 10;23:40. doi: 10.1186/s12864-021-08248-z (PMC8744286; doi:10.1186/s12864-021-08248-z)
Supplement: Supplementary file 6 — Additional file 6 : Figure S6. RNA-seq data analysis of 17 GhRAD genes in root, stem, leaf, torus, petal, stamen, pistil, calycle, − 3, − 1, 0, 1, 3, 5, 10, 20, 25 and 35 DPA ovules and 5, 10, 20 and 25 DPA fiber. [file 12864_2021_8248_MOESM6_ESM.pdf]

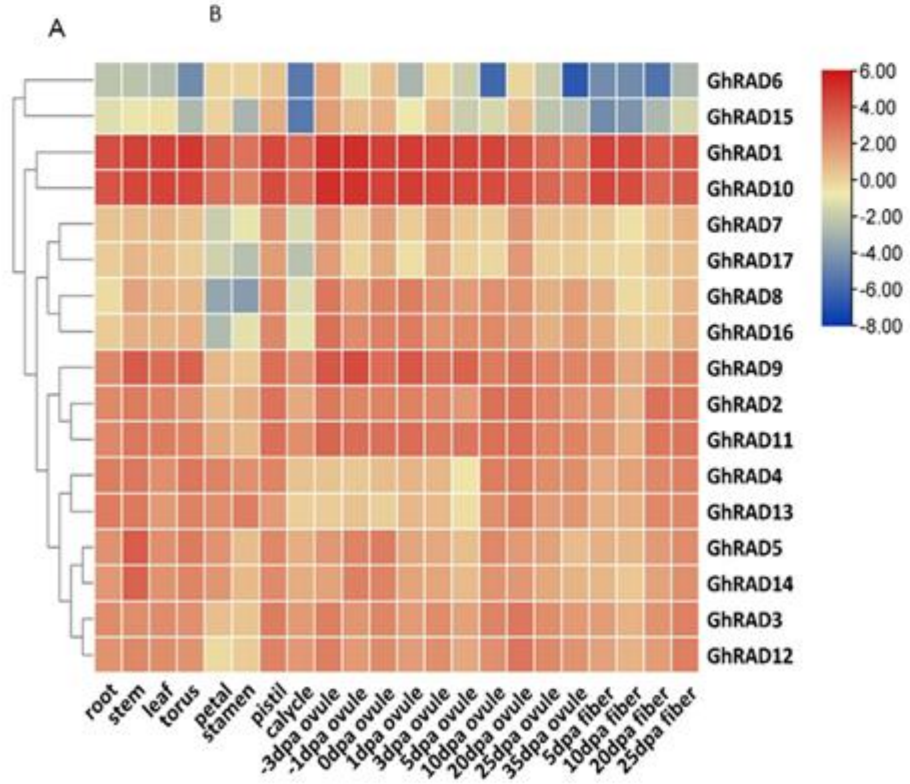

Additional file 6: Figure S6. RNA-seq data analysis of 17 *GhRAD* genes in root, stem, leaf, torus, petal, stamen, pistil, calycle, -3, -1, 0, 1, 3, 5, 10, 20, 25 and 35 DPA ovules and 5, 10, 20 and 25 DPA fiber.

Additional file 7: Table S1. *RAD5/RAD16*-like Gene Family members.

**A. thaliana**	**G. hirsutum (AD1)**	**G. barbadense (AD2)**	**G. herbaceum (A1)**	**G. arboreum (A2)**	**G. raimondii (D5)**
AT1G02670.1	Gh_A03G0848.1	Gbar_A03G011030.2	Ghe03G05870	evm.model.Ga03G1347	Gorai.005G058400.2.v2.1
AT1G05120.1	Gh_A03G1475.1	Gbar_A03G018540.1	Ghe03G15300	evm.model.Ga03G2221	Gorai.005G137100.1.v2.1
AT1G11100.1	Gh_A04G1416.1	Gbar_A04G011680.1	Ghe04G19770	evm.model.Ga04G0395	Gorai.005G213400.1.v2.1
AT1G50410.1	Gh_A05G1384.1	Gbar_A05G016300.2	Ghe05G17650	evm.model.Ga05G1742	Gorai.006G248800.1.v2.1
AT1G61140.1	Gh_A06G1100.1	Gbar_A06G013640.2	Ghe06G17300	evm.model.Ga06G1514	Gorai.009G171000.1.v2.1
AT3G16600.1	Gh_A09G1973.1	Gbar_A09G024160.1	Ghe09G28520	evm.model.Ga09G2568	Gorai.010G151100.1.v2.1
AT3G20010.1	Gh_A13G1937.1	Gbar_A13G000140.1	Ghe13G00150	evm.model.Ga13G0013	Gorai.012G136000.1.v2.1
AT5G05130.1	Gh_A13G2075.1	Gbar_A13G023790.1	Ghe13G28890	evm.model.Ga13G2713	Gorai.013G001200.1.v2.1
AT5G22750.1	Gh_D02G0515.1	Gbar_D02G012520.3			Gorai.013G258600.1.v2.1
AT5G43530.1	Gh_D02G1162.1	Gbar_D02G020440.4			
	Gh_D02G1943.1	Gbar_D04G016350.3			
	Gh_D04G1466.1	Gbar_D05G016700.2			
	Gh_D05G1552.1	Gbar_D06G013890.1			
	Gh_D06G1350.1	Gbar_D09G023820.1			
	Gh_D09G2176.1	Gbar_D13G024370.1			
	Gh_D13G0012.1	Gbar_D13G025800.1			
	Gh_D13G2334.1				

Additional file 8: Table S2. Biophysical properties of the *GhRAD* genes.

locus ID	Gene name	Chromosome	Start	End	Strand	MW(Da)	No of amino acids	pI	Gravy	Localization
Gh_A03G0848.1	GhRAD1	A03	47215607	47223353	-	100239.88	885	8.42	-0.493	Nuclear
Gh_A03G1475.1	GhRAD2	A03	94914829	94922137	-	118893.98	1067	6.25	-0.39	Nuclear
Gh_A04G1416.1	GhRAD3	scaffold1010_A04	64140	69992	-	114157.47	1034	6.57	-0.477	Nuclear
Gh_A05G1384.1	GhRAD4	A05	14387242	14395177	-	150733.96	1370	5.48	-0.432	Nuclear
Gh_A06G1100.1	GhRAD5	A06	68377219	68384995	+	146628.3	1327	5.46	-0.493	Nuclear
Gh_A09G1973.1	GhRAD6	A09	72826177	72831325	-	128203.27	1127	8.48	-0.413	Nuclear
Gh_A13G1937.1	GhRAD7	A13	78968902	78976882	-	115517.13	1037	7.18	-0.336	Nuclear
Gh_A13G2075.1	GhRAD8	scaffold3411_A13	121305	125600	-	96139.9	863	9.04	-0.253	Nuclear
Gh_D02G0515.1	GhRAD9	D02	6928584	6934790	+	123954.82	1068	6.11	-0.782	Nuclear
Gh_D02G1162.1	GhRAD10	D02	35010154	35018070	-	104556.32	926	8.77	-0.436	Nuclear
Gh_D02G1943.1	GhRAD11	D02	63210526	63219310	-	76561.78	685	8.59	-0.296	Nuclear
Gh_D04G1466.1	GhRAD12	D04	46490383	46496251	-	114589.92	1037	6.6	-0.493	Nuclear
Gh_D05G1552.1	GhRAD13	D05	14091721	14099572	-	151381.67	1374	5.47	-0.442	Nuclear
Gh_D06G1350.1	GhRAD14	D06	41408474	41416185	+	149842.04	1354	5.26	5.26	Nuclear
Gh_D09G2176.1	GhRAD15	D09	48916527	48921695	-	129935.21	1140	8.17	-0.42	Nuclear
Gh_D13G0012.1	GhRAD16	D13	82813	87108	-	96243.95	863	8.99	-0.246	Chloroplast
Gh_D13G2334.1	GhRAD17	D13	59578051	59586034	-	115444.97	1037	7.45	-0.342	Nuclear

Additional file 9: Table S3. Orthologous.paralogous gene pairs and *Ks/Ks* values of *GhRAD* gene family members.

Sr#	Chromosome	Start	End	Gene 2	Chromosome	Start	End	Ka	Ks	Ka_Ks	Chromosome
1	Gh_A02G0460	A02	6519637	6525769	Gh_D02G0515	D02	6928584	6934790	0.011031	0.054882	0.20099
2	Gh_A03G0848	A03	47215607	47223353	Gh_A08G1237	A08	83290617	83294771	0.828213	NaN	NaN
3	Gh_A03G0848	A03	47215607	47223353	Gh_A09G2124	A09	74217875	74223766	1.111688	2.1401	0.519456
4	Gh_A03G1475	A03	94914829	94922137	Gh_A09G1973	A09	72826177	72831325	0.775976	2.946018	0.263398
5	Gh_A03G0848	A03	47215607	47223353	Gh_A09G1133	A09	62724672	62733855	1.020194	NaN	NaN
6	Gh_A03G0848	A03	47215607	47223353	Gh_A09G1039	A09	60542896	60562837	0.98262	3.971861	0.247395
7	Gh_A03G0848	A03	47215607	47223353	Gh_A09G0039	A09	901666	914786	0.984335	1.99106	0.494378
8	Gh_A03G1475	A03	94914829	94922137	Gh_A09G1039	A09	60542896	60562837	1.001718	2.577196	0.388685
9	Gh_A03G1475	A03	94914829	94922137	Gh_D02G1943	D02	63210526	63219310	0.014689	0.047339	0.31029
10	Gh_A03G0848	A03	47215607	47223353	Gh_D02G1162	D02	35010154	35018070	0.024338	0.066593	0.365477
11	Gh_A03G0611	A03	17069685	17084874	Gh_D02G0515	D02	6928584	6934790	1.001178	2.186083	0.457978
12	Gh_A03G1475	A03	94914829	94922137	Gh_D04G1466	D04	46490383	46496251	0.133217	0.294153	0.452884
13	Gh_A03G0848	A03	47215607	47223353	Gh_D05G1552	D05	14091721	14099572	0.736957	NaN	NaN
14	Gh_A03G0848	A03	47215607	47223353	Gh_D06G1350	D06	41408474	41416185	0.744366	NaN	NaN
15	Gh_A03G0848	A03	47215607	47223353	Gh_D07G0311	D07	3251558	3264612	0.983625	NaN	NaN
16	Gh_A03G0848	A03	47215607	47223353	Gh_D08G2404	D08	63941593	63957460	1.011013	NaN	NaN
17	Gh_A03G0848	A03	47215607	47223353	Gh_D09G0037	D09	961839	974675	0.988109	2.358611	0.418937
18	Gh_A03G1475	A03	94914829	94922137	Gh_D09G2176	D09	48916527	48921695	0.749879	3.488427	0.214962
19	Gh_A03G0848	A03	47215607	47223353	Gh_D11G2526	D11	52098029	52112559	1.021036	NaN	NaN
20	Gh_A03G0848	A03	47215607	47223353	Gh_D12G1090	D12	37371772	37382291	1.081062	2.42564	0.445681
21	Gh_A03G0848	A03	47215607	47223353	Gh_D12G1528	D12	45937187	45943846	0.88044	NaN	NaN
22	Gh_A03G1475	A03	94914829	94922137	Gh_D13G1949	D13	54855995	54867891	1.155524	3.090684	0.373873
23	Gh_A03G0848	A03	47215607	47223353	Gh_D13G0388	D13	4282282	4289884	0.98623	2.967673	0.332324
24	Gh_A05G1384	A05	14387242	14395177	Gh_A06G1100	A06	68377219	68384995	0.138257	0.330799	0.417948
25	Gh_A05G1384	A05	14387242	14395177	Gh_A07G0254	A07	3112051	3125078	1.230647	2.101244	0.585676
26	Gh_A05G3477	A05	90320820	90327025	Gh_A09G1973	A09	72826177	72831325	1.074218	2.417	0.444443
27	Gh_A05G1384	A05	14387242	14395177	Gh_A10G1586	A10	85944027	85966360	1.128283	2.190587	0.51506
28	Gh_A05G1384	A05	14387242	14395177	Gh_A12G0440	A12	9394150	9406268	1.072474	2.336626	0.458984
29	Gh_A05G1384	A05	14387242	14395177	Gh_A13G0957	A13	51514979	51516994	0.884617	3.364526	0.262925
30	Gh_A05G3477	A05	90320820	90327025	Gh_D02G0515	D02	6928584	6934790	1.171775	3.047248	0.384535
31	Gh_A05G3477	A05	90320820	90327025	Gh_D04G1466	D04	46490383	46496251	1.009423	2.623896	0.384704
32	Gh_A05G1384	A05	14387242	14395177	Gh_D05G1552	D05	14091721	14099572	0.012399	0.034	0.364683
33	Gh_A05G1384	A05	14387242	14395177	Gh_D06G1227	D06	32075466	32089970	0.857042	NaN	NaN
34	Gh_A05G1384	A05	14387242	14395177	Gh_D06G1350	D06	41408474	41416185	0.135187	0.327411	0.412899
35	Gh_A05G1384	A05	14387242	14395177	Gh_D10G1842	D10	51578554	51597452	1.114131	1.994234	0.558676
36	Gh_A05G1384	A05	14387242	14395177	Gh_D12G1090	D12	37371772	37382291	1.12857	2.01024	0.56141
37	Gh_A05G1384	A05	14387242	14395177	Gh_D13G2271	D13	58748818	58760134	1.098135	2.423658	0.45309
38	Gh_A05G1384	A05	14387242	14395177	Gh_D13G1210	D13	36286335	36289966	0.936526	3.716904	0.251964
39	Gh_A06G1021	A06	51475680	51489467	Gh_A06G1100	A06	68377219	68384995	0.902196	NaN	NaN
40	Gh_A06G1100	A06	68377219	68384995	Gh_A06G1417	A06	96670598	96675647	1.160268	NaN	NaN
41	Gh_A06G1100	A06	68377219	68384995	Gh_A07G1308	A07	31843971	31854761	1.177899	2.309816	0.509954
42	Gh_A06G1100	A06	68377219	68384995	Gh_A09G1973	A09	72826177	72831325	0.839112	NaN	NaN

43	Gh_A06G1100	A06	68377219	68384995	Gh_A09G2124	A09	74217875	74223766	1.151607	2.186216	0.526758
44	Gh_A06G1021	A06	51475680	51489467	Gh_A09G1973	A09	72826177	72831325	0.936685	2.344099	0.399593
45	Gh_A06G1100	A06	68377219	68384995	Gh_D02G1162	D02	35010154	35018070	0.755691	NaN	NaN
46	Gh_A06G1100	A06	68377219	68384995	Gh_D05G1552	D05	14091721	14099572	0.135352	0.326952	0.41398
47	Gh_A06G1100	A06	68377219	68384995	Gh_D06G1350	D06	41408474	41416185	0.016211	0.033445	0.484713
48	Gh_A06G1417	A06	96670598	96675647	Gh_D06G1350	D06	41408474	41416185	1.181695	2.580661	0.457904
49	Gh_A06G1100	A06	68377219	68384995	Gh_D09G2329	D09	50311504	50317462	1.17693	2.102792	0.559699
50	Gh_A06G1100	A06	68377219	68384995	Gh_D13G1704	D13	51219624	51238381	1.02437	2.225586	0.46027
51	Gh_A07G1308	A07	31843971	31854761	Gh_D02G1943	D02	63210526	63219310	0.909024	2.940852	0.309102
52	Gh_A07G0254	A07	3112051	3125078	Gh_D02G1162	D02	35010154	35018070	1.037071	2.597014	0.399332
53	Gh_A07G1308	A07	31843971	31854761	Gh_D06G1350	D06	41408474	41416185	1.11775	2.619854	0.426646
54	Gh_A08G2011	A08	1.01E+08	1.01E+08	Gh_D04G1466	D04	46490383	46496251	1.006668	2.375036	0.423854
55	Gh_A09G1973	A09	72826177	72831325	Gh_A10G1586	A10	85944027	85966360	0.828945	3.690504	0.224616
56	Gh_A09G1039	A09	60542896	60562837	Gh_A13G1937	A13	78968902	78976882	1.007635	2.536438	0.397264
57	Gh_A09G2124	A09	74217875	74223766	Gh_D02G1162	D02	35010154	35018070	1.154342	2.008225	0.574807
58	Gh_A09G1039	A09	60542896	60562837	Gh_D02G0515	D02	6928584	6934790	0.685609	NaN	NaN
59	Gh_A09G2124	A09	74217875	74223766	Gh_D02G0515	D02	6928584	6934790	0.035628	0.37311	0.09549
60	Gh_A09G1973	A09	72826177	72831325	Gh_D02G1162	D02	35010154	35018070	0.755695	1.822572	0.414631
61	Gh_A09G1973	A09	72826177	72831325	Gh_D08G2404	D08	63941593	63957460	0.992601	4.577292	0.216853
62	Gh_A09G1973	A09	72826177	72831325	Gh_D09G2176	D09	48916527	48921695	0.010778	0.039242	0.274663
63	Gh_A09G2044	A09	73516660	73534062	Gh_D13G2334	D13	59578051	59586034	0.951393	NaN	NaN
64	Gh_A09G1039	A09	60542896	60562837	Gh_D13G2334	D13	59578051	59586034	1.059383	2.455365	0.431456
65	Gh_A10G1427	A10	76985307	76991322	Gh_D02G0515	D02	6928584	6934790	0.033926	0.336761	0.100741
66	Gh_A10G1586	A10	85944027	85966360	Gh_D09G2176	D09	48916527	48921695	0.841103	3.093621	0.271883
67	Gh_A12G1277	A12	67781096	67786286	Gh_D02G1943	D02	63210526	63219310	1.005292	2.835226	0.354572
68	Gh_A12G0603	A12	15543735	15548398	Gh_D02G0515	D02	6928584	6934790	0.622097	1.961645	0.31713
69	Gh_A12G0391	A12	7494757	7501718	Gh_D04G1466	D04	46490383	46496251	1.060721	NaN	NaN
70	Gh_A12G0440	A12	9394150	9406268	Gh_D04G1466	D04	46490383	46496251	0.952722	2.043412	0.466241
71	Gh_A12G0974	A12	60861679	60871973	Gh_D05G1552	D05	14091721	14099572	1.101283	1.860307	0.59199
72	Gh_A12G0603	A12	15543735	15548398	Gh_D05G1552	D05	14091721	14099572	0.83563	2.670345	0.31293
73	Gh_A12G1277	A12	67781096	67786286	Gh_D06G1350	D06	41408474	41416185	0.869525	NaN	NaN
74	Gh_A12G0391	A12	7494757	7501718	Gh_D09G2176	D09	48916527	48921695	1.035592	3.76506	0.275053
75	Gh_A12G0603	A12	15543735	15548398	Gh_D13G2334	D13	59578051	59586034	0.838298	NaN	NaN
76	Gh_A12G0974	A12	60861679	60871973	Gh_D13G2334	D13	59578051	59586034	0.94209	2.75085	0.342472
77	Gh_A13G0346	A13	4554369	4559010	Gh_D02G0515	D02	6928584	6934790	0.883843	2.588428	0.341459
78	Gh_A13G0346	A13	4554369	4559010	Gh_D04G1466	D04	46490383	46496251	1.068222	NaN	NaN
79	Gh_A13G0957	A13	51514979	51516994	Gh_D04G1466	D04	46490383	46496251	0.968338	3.552729	0.272562
80	Gh_A13G0957	A13	51514979	51516994	Gh_D05G1552	D05	14091721	14099572	0.877753	2.771685	0.316686
81	Gh_A13G1937	A13	78968902	78976882	Gh_D09G1060	D09	36675619	36691099	1.032619	2.350489	0.439321
82	Gh_A13G1937	A13	78968902	78976882	Gh_D12G1090	D12	37371772	37382291	1.003207	3.260708	0.307665
83	Gh_A13G1937	A13	78968902	78976882	Gh_D13G2334	D13	59578051	59586034	0.004618	0.027033	0.170821
84	Gh_A13G1884	A13	78505861	78517333	Gh_D13G2334	D13	59578051	59586034	1.051772	2.470561	0.425722
85	Gh_A13G1937	A13	78968902	78976882	Gh_D13G2271	D13	58748818	58760134	1.052835	3.427858	0.307141
86	Gh_D02G1943	D02	63210526	63219310	Gh_D04G1466	D04	46490383	46496251	0.101878	0.297186	0.342807
87	Gh_D02G1162	D02	35010154	35018070	Gh_D05G1552	D05	14091721	14099572	0.744064	3.44483	0.215994

88	Gh_D02G0515	D02	6928584	6934790	Gh_D09G2329	D09	50311504	50317462	0.036962	0.395863	0.093371
89	Gh_D02G0515	D02	6928584	6934790	Gh_D10G1668	D10	46113163	46119283	0.036401	0.34925	0.104227
90	Gh_D02G1162	D02	35010154	35018070	Gh_D10G1842	D10	51578554	51597452	0.912721	4.11592	0.221754
91	Gh_D02G1162	D02	35010154	35018070	Gh_D11G2526	D11	52098029	52112559	1.050115	3.637808	0.288667
92	Gh_D02G0515	D02	6928584	6934790	Gh_D12G1090	D12	37371772	37382291	0.745863	4.089962	0.182364
93	Gh_D02G0515	D02	6928584	6934790	Gh_D13G2271	D13	58748818	58760134	0.703987	NaN	NaN
94	Gh_D04G1466	D04	46490383	46496251	Gh_D06G1227	D06	32075466	32089970	1.050689	1.791951	0.586338
95	Gh_D04G1466	D04	46490383	46496251	Gh_D07G0311	D07	3251558	3264612	1.017862	2.721286	0.374037
96	Gh_D04G1466	D04	46490383	46496251	Gh_D08G2404	D08	63941593	63957460	1.032877	1.912864	0.539964
97	Gh_D04G1466	D04	46490383	46496251	Gh_D11G2526	D11	52098029	52112559	1.080885	2.293653	0.471251
98	Gh_D04G1466	D04	46490383	46496251	Gh_D12G0441	D12	7251640	7263795	0.981331	1.86753	0.52547
99	Gh_D04G1466	D04	46490383	46496251	Gh_D12G0274	D12	3709247	3715907	0.990554	NaN	NaN
100	Gh_D04G1466	D04	46490383	46496251	Gh_D13G0388	D13	4282282	4289884	0.994359	2.400767	0.414184
101	Gh_D04G1466	D04	46490383	46496251	Gh_D13G0388	D13	4282282	4289884	0.994359	2.400767	0.414184
102	Gh_D05G1552	D05	14091721	14099572	Gh_D06G1350	D06	41408474	41416185	0.130687	0.323551	0.403914
103	Gh_D05G2412	D05	24140620	24144133	Gh_D06G1350	D06	41408474	41416185	1.005256	2.569889	0.391167
104	Gh_D05G1552	D05	14091721	14099572	Gh_D07G0311	D07	3251558	3264612	1.315021	1.870258	0.703123
105	Gh_D05G1552	D05	14091721	14099572	Gh_D12G1090	D12	37371772	37382291	1.133414	1.920909	0.59004
106	Gh_D05G1552	D05	14091721	14099572	Gh_D13G1210	D13	36286335	36289966	0.965434	2.970358	0.325023
107	Gh_D06G1350	D06	41408474	41416185	Gh_D06G1763	D06	57383738	57388782	1.150679	3.384775	0.339957
108	Gh_D06G1227	D06	32075466	32089970	Gh_D09G2176	D09	48916527	48921695	0.934844	2.093993	0.446441
109	Gh_D09G2176	D09	48916527	48921695	Gh_D11G2526	D11	52098029	52112559	0.993117	2.167122	0.458265
110	Gh_D09G2176	D09	48916527	48921695	Gh_D12G0441	D12	7251640	7263795	0.985463	4.038207	0.244035
111	Gh_D09G2250	D09	49746574	49760959	Gh_D13G2334	D13	59578051	59586034	0.99343	3.237236	0.306876
112	Gh_D09G1060	D09	36675619	36691099	Gh_D13G2334	D13	59578051	59586034	1.031233	2.282641	0.451772
