# Supplementary material for: RAD gene family analysis in cotton provides some key genes for flowering and stress tolerance in upland cotton G. hirsutum
Source: BMC Genomics. 2022 Jan 10;23:40. doi: 10.1186/s12864-021-08248-z (PMC8744286; doi:10.1186/s12864-021-08248-z)
Supplement: Supplementary file 7 — Additional file 7 : Table S1. RAD5/RAD16-like Gene Family members. [file 12864_2021_8248_MOESM7_ESM.pdf]

Additional file 7: Table S1. *RAD5/RAD16*-like Gene Family members.

**A. thaliana**	**G. hirsutum (AD1)**	**G. barbadense (AD2)**	**G. herbaceum (A1)**	**G. arboreum (A2)**	**G. raimondii (D5)**
AT1G02670.1	Gh_A03G0848.1	Gbar_A03G011030.2	Ghe03G05870	evm.model.Ga03G1347	Gorai.005G058400.2.v2.1
AT1G05120.1	Gh_A03G1475.1	Gbar_A03G018540.1	Ghe03G15300	evm.model.Ga03G2221	Gorai.005G137100.1.v2.1
AT1G11100.1	Gh_A04G1416.1	Gbar_A04G011680.1	Ghe04G19770	evm.model.Ga04G0395	Gorai.005G213400.1.v2.1
AT1G50410.1	Gh_A05G1384.1	Gbar_A05G016300.2	Ghe05G17650	evm.model.Ga05G1742	Gorai.006G248800.1.v2.1
AT1G61140.1	Gh_A06G1100.1	Gbar_A06G013640.2	Ghe06G17300	evm.model.Ga06G1514	Gorai.009G171000.1.v2.1
AT3G16600.1	Gh_A09G1973.1	Gbar_A09G024160.1	Ghe09G28520	evm.model.Ga09G2568	Gorai.010G151100.1.v2.1
AT3G20010.1	Gh_A13G1937.1	Gbar_A13G000140.1	Ghe13G00150	evm.model.Ga13G0013	Gorai.012G136000.1.v2.1
AT5G05130.1	Gh_A13G2075.1	Gbar_A13G023790.1	Ghe13G28890	evm.model.Ga13G2713	Gorai.013G001200.1.v2.1
AT5G22750.1	Gh_D02G0515.1	Gbar_D02G012520.3			Gorai.013G258600.1.v2.1
AT5G43530.1	Gh_D02G1162.1	Gbar_D02G020440.4			
	Gh_D02G1943.1	Gbar_D04G016350.3			
	Gh_D04G1466.1	Gbar_D05G016700.2			
	Gh_D05G1552.1	Gbar_D06G013890.1			
	Gh_D06G1350.1	Gbar_D09G023820.1			
	Gh_D09G2176.1	Gbar_D13G024370.1			
	Gh_D13G0012.1	Gbar_D13G025800.1			
	Gh_D13G2334.1				
