# Supplementary material for: RAD gene family analysis in cotton provides some key genes for flowering and stress tolerance in upland cotton G. hirsutum
Source: BMC Genomics. 2022 Jan 10;23:40. doi: 10.1186/s12864-021-08248-z (PMC8744286; doi:10.1186/s12864-021-08248-z)
Supplement: Supplementary file 8 — Additional file 8 : Table S2. Biophysical properties of the GhRAD genes. [file 12864_2021_8248_MOESM8_ESM.pdf]

Additional file 8: Table S2. Biophysical properties of the *GhRAD* genes.

locus ID	Gene name	Chromosome	Start	End	Strand	MW(Da)	No of amino acids	pI	Gravy	Localization
Gh_A03G0848.1	GhRAD1	A03	47215607	47223353	-	100239.88	885	8.42	-0.493	Nuclear
Gh_A03G1475.1	GhRAD2	A03	94914829	94922137	-	118893.98	1067	6.25	-0.39	Nuclear
Gh_A04G1416.1	GhRAD3	scaffold1010_A04	64140	69992	-	114157.47	1034	6.57	-0.477	Nuclear
Gh_A05G1384.1	GhRAD4	A05	14387242	14395177	-	150733.96	1370	5.48	-0.432	Nuclear
Gh_A06G1100.1	GhRAD5	A06	68377219	68384995	+	146628.3	1327	5.46	-0.493	Nuclear
Gh_A09G1973.1	GhRAD6	A09	72826177	72831325	-	128203.27	1127	8.48	-0.413	Nuclear
Gh_A13G1937.1	GhRAD7	A13	78968902	78976882	-	115517.13	1037	7.18	-0.336	Nuclear
Gh_A13G2075.1	GhRAD8	scaffold3411_A13	121305	125600	-	96139.9	863	9.04	-0.253	Nuclear
Gh_D02G0515.1	GhRAD9	D02	6928584	6934790	+	123954.82	1068	6.11	-0.782	Nuclear
Gh_D02G1162.1	GhRAD10	D02	35010154	35018070	-	104556.32	926	8.77	-0.436	Nuclear
Gh_D02G1943.1	GhRAD11	D02	63210526	63219310	-	76561.78	685	8.59	-0.296	Nuclear
Gh_D04G1466.1	GhRAD12	D04	46490383	46496251	-	114589.92	1037	6.6	-0.493	Nuclear
Gh_D05G1552.1	GhRAD13	D05	14091721	14099572	-	151381.67	1374	5.47	-0.442	Nuclear
Gh_D06G1350.1	GhRAD14	D06	41408474	41416185	+	149842.04	1354	5.26	5.26	Nuclear
Gh_D09G2176.1	GhRAD15	D09	48916527	48921695	-	129935.21	1140	8.17	-0.42	Nuclear
Gh_D13G0012.1	GhRAD16	D13	82813	87108	-	96243.95	863	8.99	-0.246	Chloroplast
Gh_D13G2334.1	GhRAD17	D13	59578051	59586034	-	115444.97	1037	7.45	-0.342	Nuclear
