# Supplementary material for: RAD gene family analysis in cotton provides some key genes for flowering and stress tolerance in upland cotton G. hirsutum
Source: BMC Genomics. 2022 Jan 10;23:40. doi: 10.1186/s12864-021-08248-z (PMC8744286; doi:10.1186/s12864-021-08248-z)
Supplement: Supplementary file 10 — Additional file 10 : Table S4. Orthologous.paralogous gene pairs and Ks/Ks values of GbRAD gene family members. [file 12864_2021_8248_MOESM10_ESM.pdf]

Additional file 10: Table S4. Orthologous.paralogous gene pairs and *Ks/Ks* values of *GbRAD* gene family members.

Sr#	Gene 1	Chromosome	Start	End	Gene 2	Chromosome	Start	End	Ka	Ks	Ka_Ks
1	Gbar_A03G018540.1	A03	99721182	99728789	Gbar_A04G011680.1	A04	76657286	76664656	0.130818	0.308159	0.424514
2	Gbar_A03G018540.1	A03	99721182	99728789	Gbar_D02G020440.4	D02	62928457	62937954	0.014452	0.044001	0.328448
3	Gbar_A03G011030.2	A03	51900347	51908613	Gbar_D02G012520.3	D02	35590199	35598441	0.004871	0.034728	0.140258
4	Gbar_A03G018540.1	A03	99721182	99728789	Gbar_D04G016350.3	D04	47474956	47482391	0.13687	0.300588	0.45534
5	Gbar_A04G011680.1	A04	76657286	76664656	Gbar_D02G020440.4	D02	62928457	62937954	0.125279	0.313908	0.399096
6	Gbar_A04G011680.1	A04	76657286	76664656	Gbar_D04G016350.3	D04	47474956	47482391	0.009321	0.033904	0.274918
7	Gbar_A05G016300.2	A05	15291081	15300531	Gbar_A06G013640.2	A06	80811032	80821135	0.137753	0.332131	0.414755
8	Gbar_A05G016300.2	A05	15291081	15300531	Gbar_D05G016700.2	D05	14540149	14549582	0.011745	0.039657	0.296167
9	Gbar_A05G016300.2	A05	15291081	15300531	Gbar_D06G013890.1	D06	40507897	40517804	0.132138	0.325564	0.405876
10	Gbar_A06G013640.2	A06	80811032	80821135	Gbar_D05G016700.2	D05	14540149	14549582	0.135012	0.326411	0.413626
11	Gbar_A06G013640.2	A06	80811032	80821135	Gbar_D06G013890.1	D06	40507897	40517804	0.016506	0.036678	0.450031
12	Gbar_A09G024160.1	A09	75812595	75817898	Gbar_D09G023820.1	D09	49568359	49573678	0.012136	0.047031	0.258037
13	Gbar_A13G023790.1	A13	1.08E+08	1.08E+08	Gbar_D13G024370.1	D13	59316920	59325169	0.006815	0.025769	0.264469
14	Gbar_D02G020440.4	D02	62928457	62937954	Gbar_D04G016350.3	D04	47474956	47482391	0.130711	0.304405	0.429399
15	Gbar_D05G016700.2	D05	14540149	14549582	Gbar_D06G013890.1	D06	40507897	40517804	0.127785	0.319801	0.399577
