# Supplementary material for: RAD gene family analysis in cotton provides some key genes for flowering and stress tolerance in upland cotton G. hirsutum
Source: BMC Genomics. 2022 Jan 10;23:40. doi: 10.1186/s12864-021-08248-z (PMC8744286; doi:10.1186/s12864-021-08248-z)
Supplement: Supplementary file 11 — Additional file 11 : Table S5. MicroRNA (miRNA) target sites of GhRAD genes. [file 12864_2021_8248_MOESM11_ESM.pdf]

**Additional file 11: Table S5**. MicroRNA (miRNA) target sites of *GhRAD* genes.

| miRNA_ Acc. | Target_ Acc. | Expectation | UPE$ | miRNA_ start | miRNA_ end | Target_ start | Target_ end | miRNA_ aligned_ fragment | alignment | Target_ aligned_ fragment | Inhibition | Target_ Desc. | Multiplicity |
|---|---|---|---|---|---|---|---|---|---|---|---|---|---|
| Ghi-miRN1440 | GhRAD3 | 3 | -1 | 1 | 21 | 2150 | 2170 | UUUCCCAGGAAUUCAGUAGUA | :::::::: :::: ::::: | AUCUACUGAACUCCUUGGAAA | Translation | | 1 |
| Ghi-miRN1440 | GhRAD12 | 3 | -1 | 1 | 21 | 2156 | 2176 | UUUCCCAGGAAUUCAGUAGUA | :::::::: :::: ::::: | AUCUACUGAACUCCUUGGAAA | Translation | | 1 |
| Ghi-miR8645a | GhRAD8 | 3.5 | -1 | 1 | 22 | 2306 | 2327 | UUGGUACCUGAACUUGACGUCU | ::.: :::.:: :::::::.::: | AGGCUUCAGGUGCAGGUAUCAA | Translation | | 1 |
| Ghi-miR8645a | GhRAD16 | 3.5 | -1 | 1 | 22 | 2306 | 2327 | UUGGUACCUGAACUUGACGUCU | ::.: :::.:: :::::::.::: | AGGCUUCAGGUGCAGGUAUCAA | Translation | | 1 |
| Ghi-miR8645b | GhRAD8 | 3.5 | -1 | 1 | 22 | 2306 | 2327 | UUGGUACCUGAACUUGACGUCU | ::.: :::.:: :::::::.::: | AGGCUUCAGGUGCAGGUAUCAA | Translation | | 1 |
| Ghi-miR8645b | GhRAD16 | 3.5 | -1 | 1 | 22 | 2306 | 2327 | UUGGUACCUGAACUUGACGUCU | ::.: :::.:: :::::::.::: | AGGCUUCAGGUGCAGGUAUCAA | Translation | | 1 |
| Ghi-miRN1393a | GhRAD14 | 3.5 | -1 | 1 | 22 | 866 | 887 | GCAUCAGAGGAGUCAAGCAGGU | : ::::.:.:::::: :. | GUGAGAUUGAUUUCUCUGAUGU | Cleavage | | 1 |
| Ghi-miRN1393b | GhRAD14 | 3.5 | -1 | 1 | 22 | 866 | 887 | GCAUCAGAGGAGUCAAGCAGGU | : ::::.:.:::::: :. | GUGAGAUUGAUUUCUCUGAUGU | Cleavage | | 1 |
| Ghi-miRN1393c | GhRAD14 | 3.5 | -1 | 1 | 22 | 866 | 887 | GCAUCAGAGGAGUCAAGCAGGU | : ::::.:.:::::: :. | GUGAGAUUGAUUUCUCUGAUGU | Cleavage | | 1 |
| Ghi-miRN1393d | GhRAD14 | 3.5 | -1 | 1 | 22 | 866 | 887 | GCAUCAGAGGAGUCAAGCAGGU | : ::::.:.:::::: :. | GUGAGAUUGAUUUCUCUGAUGU | Cleavage | | 1 |
| Ghi-miRN1394 | GhRAD8 | 3.5 | -1 | 1 | 22 | 2512 | 2533 | ACUCUCUUCCAAAGGCUUCAAG | :::::.::::::: . :: | ACAGAAGCUUUUGGAAGGAAGG | Cleavage | | 1 |
| Ghi-miRN1394 | GhRAD16 | 3.5 | -1 | 1 | 22 | 2512 | 2533 | ACUCUCUUCCAAAGGCUUCAAG | :::::.::::::: . :: | ACAGAAGCUUUUGGAAGGAAGG | Cleavage | | 1 |
| Ghi-miRN1396a | GhRAD13 | 3.5 | -1 | 1 | 21 | 3102 | 3122 | GGAAAUCUUUGGGGAGAGUGA | :::::.:.::.::: :: .: | AAACUCUUCUCAGAGAUUGUC | Cleavage | | 1 |

| | | | | | | | | | | | | | |
|---|---|---|---|---|---|---|---|---|---|---|---|---|---|
| Ghi-miRN1396a | GhRAD4 | 3.5 | -1 | 1 | 21 | 3090 | 3110 | GGAAAUCUUUGGGGAGAGUGA | :::::.:.::.:::<br>:: .: | AAACUCUUCUCAGAGAUUGUC | Cleavage | | 1 |
| Ghi-miRN1396b | GhRAD4 | 3.5 | -1 | 1 | 21 | 3090 | 3110 | GGAAAUCUUUGGGGAGAGUGA | :::::.:.::.:::<br>:: .: | AAACUCUUCUCAGAGAUUGUC | Cleavage | | 1 |
| Ghi-miRN1396b | GhRAD13 | 3.5 | -1 | 1 | 21 | 3102 | 3122 | GGAAAUCUUUGGGGAGAGUGA | :::::.:.::.:::<br>:: .: | AAACUCUUCUCAGAGAUUGUC | Cleavage | | 1 |
| Ghi-miRN1419a | GhRAD15 | 3.5 | -1 | 1 | 21 | 3032 | 3052 | AGAAUCUAGUUUUUCUCUUGC | :.::<br>:..::.:.:::::: | AGAGGAAAGGAAUUGGAUUCU | Cleavage | | 1 |
| Ghi-miRN1419a | GhRAD6 | 3.5 | -1 | 1 | 21 | 2993 | 3013 | AGAAUCUAGUUUUUCUCUUGC | :.::<br>:..::.:.:::::: | AGAGGAAAGGAAUUGGAUUCU | Cleavage | | 1 |
| Ghi-miR8677 | GhRAD15 | 4 | -1 | 1 | 21 | 3117 | 3137 | AAUGAAUCUAGUUUCUCUCUU | .::::::::::.:<br>:.::: | GAGAGAGAAACUGGUAUUAUU | Cleavage | | 2 |
| Ghi-miRN1374 | GhRAD10 | 4 | -1 | 1 | 21 | 83 | 103 | GAUGGGUGAGGGGGUAAGACA | : ::::<br>:.:.::::. ::: | UUUCUUUCUCUCUCACUGAUC | Cleavage | | 1 |
| Ghi-miRN1393a | GhRAD5 | 4 | -1 | 1 | 22 | 863 | 884 | GCAUCAGAGGAGUCAAGCAGGU | ::<br>::..:.::::::::<br>. | CAAUGAAUGGUUUCUCUGAUGU | Cleavage | | 1 |
| Ghi-miRN1393b | GhRAD5 | 4 | -1 | 1 | 22 | 863 | 884 | GCAUCAGAGGAGUCAAGCAGGU | ::<br>::..:.::::::::<br>. | CAAUGAAUGGUUUCUCUGAUGU | Cleavage | | 1 |
| Ghi-miRN1393c | GhRAD5 | 4 | -1 | 1 | 22 | 863 | 884 | GCAUCAGAGGAGUCAAGCAGGU | ::<br>::..:.::::::::<br>. | CAAUGAAUGGUUUCUCUGAUGU | Cleavage | | 1 |
| Ghi-miRN1393d | GhRAD5 | 4 | -1 | 1 | 22 | 863 | 884 | GCAUCAGAGGAGUCAAGCAGGU | ::<br>::..:.::::::::<br>. | CAAUGAAUGGUUUCUCUGAUGU | Cleavage | | 1 |
| Ghi-miRN1396a | GhRAD12 | 4 | -1 | 1 | 21 | 2129 | 2149 | GGAAAUCUUUGGGGAGAGUGA | ::<br>::.::.:.:::<br>:::: | AGACACUUCCUAGAGAAUUCC | Cleavage | | 1 |
| Ghi-miRN1396b | GhRAD12 | 4 | -1 | 1 | 21 | 2129 | 2149 | GGAAAUCUUUGGGGAGAGUGA | ::<br>::.::.:.:::<br>:::: | AGACACUUCCUAGAGAAUUCC | Cleavage | | 1 |
| Ghi-miRN1438 | GhRAD15 | 4 | -1 | 1 | 21 | 3032 | 3052 | UGAAUCUAGUUUCUCUCUUAC | :.::<br>::.::.:.::::: | AGAGGAAAGGAAUUGGAUUCU | Cleavage | | 3 |
| Ghi-miRN1438 | GhRAD6 | 4 | -1 | 1 | 21 | 2993 | 3013 | UGAAUCUAGUUUCUCUCUUAC | :.::<br>::.::.:.::::: | AGAGGAAAGGAAUUGGAUUCU | Cleavage | | 1 |
| Ghi-miRN1438 | GhRAD10 | 4 | -1 | 1 | 21 | 2282 | 2302 | UGAAUCUAGUUUCUCUCUUAC | :.:. :::::.<br>::::::: | UGAGGGAAGAAAUCAGAUUCA | Cleavage | | 1 |

| | | | | | | | | | | | | | |
|---|---|---|---|---|---|---|---|---|---|---|---|---|---|
| Ghi-miRN1438 | GhRAD1 | 4 | -1 | 1 | 21 | 2159 | 2179 | UGAAUCUAGUUUCUCUCUUAC | :.:. :::::. ::::::: | UGAGGGAAGAAAUCAGAUUCA | Cleavage | | 1 |
| Ghi-miRN1443a | GhRAD8 | 4 | -1 | 1 | 21 | 2033 | 2053 | UCUUUGAUGAUGUUACUGACC | : : ::.::::::::.:: | ACACUGAAAUAUCAUCAAGGA | Cleavage | | 2 |
| Ghi-miRN1443a | GhRAD16 | 4 | -1 | 1 | 21 | 2033 | 2053 | UCUUUGAUGAUGUUACUGACC | : : ::.::::::::.:: | ACACUGAAAUAUCAUCAAGGA | Cleavage | | 1 |
| Ghi-miRN1443b | GhRAD8 | 4 | -1 | 1 | 21 | 2033 | 2053 | UCUUUGAUGAUGUUACUGACC | : : ::.::::::::.:: | ACACUGAAAUAUCAUCAAGGA | Cleavage | | 2 |
| Ghi-miRN1443b | GhRAD16 | 4 | -1 | 1 | 21 | 2033 | 2053 | UCUUUGAUGAUGUUACUGACC | : : ::.::::::::.:: | ACACUGAAAUAUCAUCAAGGA | Cleavage | | 1 |
| Ghi-miRN1461 | GhRAD9 | 4 | -1 | 1 | 21 | 2155 | 2175 | CAGGAAGAGGAAGAUGAAAUA | :::..:.:::.::: :..: | GUUUUUGUUUUCUUCUUUUUA | Cleavage | | 1 |
| Ghi-miRN1480a | GhRAD15 | 4 | -1 | 1 | 21 | 1089 | 1109 | UUGUGAUGUGGUUUGAAUUUC | ::: : ::::::.:::: :: | UAAAAUAAAACCAUAUCAGAA | Cleavage | | 2 |
| Ghi-miRN1480a | GhRAD6 | 4 | -1 | 1 | 21 | 1089 | 1109 | UUGUGAUGUGGUUUGAAUUUC | ::: : ::::::.:::: :: | UAAAAUAAAACCAUAUCAGAA | Cleavage | | 2 |
| Ghi-miRN1480b | GhRAD15 | 4 | -1 | 1 | 21 | 1089 | 1109 | UUGUGAUGUGGUUUGAAUUUC | ::: : ::::::.:::: :: | UAAAAUAAAACCAUAUCAGAA | Cleavage | | 2 |
| Ghi-miRN1480b | GhRAD6 | 4 | -1 | 1 | 21 | 1089 | 1109 | UUGUGAUGUGGUUUGAAUUUC | ::: : ::::::.:::: :: | UAAAAUAAAACCAUAUCAGAA | Cleavage | | 2 |
| Ghi-miRN1493 | GhRAD9 | 4 | -1 | 1 | 21 | 1669 | 1689 | UAACAAGAGGACCAGUUUGCU | :::.: ::..::::::: | GAGAAAUUUGUUUUCUUGUUA | Cleavage | | 1 |
| Ghi-miRN1496 | GhRAD13 | 4 | -1 | 1 | 21 | 1806 | 1826 | UCAACAGGAGGACUAGUUUGC | ::.:::::::::: : ::. | UGAAGCUAGUCCUCCAGAUGG | Cleavage | | 1 |
| Ghi-miRN1496 | GhRAD4 | 4 | -1 | 1 | 21 | 1800 | 1820 | UCAACAGGAGGACUAGUUUGC | ::.:::::::::: : ::. | UGAAGCUAGUCCUCCAGAUGG | Cleavage | | 1 |
| Ghi-miRN1496 | GhRAD14 | 4 | -1 | 1 | 21 | 1704 | 1724 | UCAACAGGAGGACUAGUUUGC | ::.:::::::::: : ::. | UGAAGCUAGUCCUCCGGAUGG | Cleavage | | 1 |
| Ghi-miRN1496 | GhRAD5 | 4 | -1 | 1 | 21 | 1701 | 1721 | UCAACAGGAGGACUAGUUUGC | ::.:::::::::: : ::. | UGAAGCUAGUCCUCCGGAUGG | Cleavage | | 1 |

| | | | | | | | | | | | | | |
|---|---|---|---|---|---|---|---|---|---|---|---|---|---|
| Ghi-miRN1496 | GhRAD2 | 4 | -1 | 1 | 21 | 1970 | 1990 | UCAACAGGAGGACUAGUUUGC | ::.. :::.::: :::::: | CAAAGUCAGUUCUCAUGUUGA | Cleavage | | 1 |
| Ghi-miR397c | GhRAD13 | 4.5 | -1 | 1 | 21 | 3477 | 3497 | UCAUUGAGUGCAGCGUUGAUG | ::::: ::::: ::..::: | AAUCAAGGCUGCCCUUGAUGU | Cleavage | | 1 |
| Ghi-miR397c | GhRAD4 | 4.5 | -1 | 1 | 21 | 3465 | 3485 | UCAUUGAGUGCAGCGUUGAUG | ::::: ::::: ::..::: | AAUCAAGGCUGCCCUUGAUGU | Cleavage | | 1 |
| Ghi-miR397d | GhRAD13 | 4.5 | -1 | 1 | 21 | 3477 | 3497 | UCAUUGAGUGCAGCGUUGAUG | ::::: ::::: ::..::: | AAUCAAGGCUGCCCUUGAUGU | Cleavage | | 1 |
| Ghi-miR397d | GhRAD4 | 4.5 | -1 | 1 | 21 | 3465 | 3485 | UCAUUGAGUGCAGCGUUGAUG | ::::: ::::: ::..::: | AAUCAAGGCUGCCCUUGAUGU | Cleavage | | 1 |
| Ghi-miR397e | GhRAD13 | 4.5 | -1 | 1 | 21 | 3477 | 3497 | UCAUUGAGUGCAGCGUUGAUG | ::::: ::::: ::..::: | AAUCAAGGCUGCCCUUGAUGU | Cleavage | | 1 |
| Ghi-miR397e | GhRAD4 | 4.5 | -1 | 1 | 21 | 3465 | 3485 | UCAUUGAGUGCAGCGUUGAUG | ::::: ::::: ::..::: | AAUCAAGGCUGCCCUUGAUGU | Cleavage | | 1 |
| Ghi-miR398c | GhRAD15 | 4.5 | -1 | 1 | 21 | 1844 | 1864 | UGUGUUCUCAGGUCACCCCUU | ::::::.:: :::.::.. | CCAGGGUGAUCUCAGAGCAUG | Cleavage | | 1 |
| Ghi-miR398c | GhRAD6 | 4.5 | -1 | 1 | 21 | 1844 | 1864 | UGUGUUCUCAGGUCACCCCUU | ::::::.:: :::.::.. | CCAGGGUGAUCUCAGAGCAUG | Cleavage | | 1 |
| Ghi-miR398d | GhRAD15 | 4.5 | -1 | 1 | 21 | 1844 | 1864 | UGUGUUCUCAGGUCACCCCUU | ::::::.:: :::.::.. | CCAGGGUGAUCUCAGAGCAUG | Cleavage | | 1 |
| Ghi-miR398d | GhRAD6 | 4.5 | -1 | 1 | 21 | 1844 | 1864 | UGUGUUCUCAGGUCACCCCUU | ::::::.:: :::.::.. | CCAGGGUGAUCUCAGAGCAUG | Cleavage | | 1 |
| Ghi-miR7495 | GhRAD11 | 4.5 | -1 | 1 | 21 | 1499 | 1519 | UUACUUUAGAUGUCUCCUUCA | .:: :::::: ::::: :: | CUGAGCAGACAUAUAAAGAAA | Cleavage | | 1 |
| Ghi-miR8677 | GhRAD6 | 4.5 | -1 | 1 | 21 | 3078 | 3098 | AAUGAAUCUAGUUUCUCUCUU | ::::::::::::..: :...:: | AAGAGAGAAACUGGUAUUGUU | Cleavage | | 2 |
| Ghi-miR8677 | GhRAD1 | 4.5 | -1 | 1 | 21 | 2161 | 2181 | AAUGAAUCUAGUUUCUCUCUU | :.:. :::::. :::::::: | AGGGAAGAGAAAUCAGAUUCAUG | Translation | | 1 |
| Ghi-miR8677 | GhRAD10 | 4.5 | -1 | 1 | 21 | 2284 | 2304 | AAUGAAUCUAGUUUCUCUCUU | :.:. :::::. :::::::: | AGGGAAGAGAAAUCAGAUUCAUG | Translation | | 1 |
| Ghi-miR8745a | GhRAD8 | 4.5 | -1 | 1 | 21 | 826 | 845 | UCAACGGAGUUGGGAGACAAA | ::::: :::::::.::.: ::: | UUUGU-UCCCAAUUCUGGUGA | Cleavage | | 1 |
| Ghi-miR8745a | GhRAD16 | 4.5 | -1 | 1 | 21 | 826 | 845 | UCAACGGAGUUGGGAGACAAA | ::::: :::::::.::.: ::: | UUUGU-UCCCAAUUCUGGUGA | Cleavage | | 1 |
| Ghi-miR8745b | GhRAD8 | 4.5 | -1 | 1 | 21 | 826 | 845 | UCAACGGAGUUGGGAGACAAA | ::::: :::::::.::.: ::: | UUUGU-UCCCAAUUCUGGUGA | Cleavage | | 1 |
| Ghi-miR8745b | GhRAD16 | 4.5 | -1 | 1 | 21 | 826 | 845 | UCAACGGAGUUGGGAGACAAA | ::::: :::::::.::.: ::: | UUUGU-UCCCAAUUCUGGUGA | Cleavage | | 1 |
| Ghi-miR8745c | GhRAD8 | 4.5 | -1 | 1 | 21 | 826 | 845 | UCAACGGAGUUGGGAGACAAA | ::::: :::::::.::.: ::: | UUUGU-UCCCAAUUCUGGUGA | Cleavage | | 1 |
| Ghi-miR8745c | GhRAD16 | 4.5 | -1 | 1 | 21 | 826 | 845 | UCAACGGAGUUGGGAGACAAA | ::::: :::::::.::.: ::: | UUUGU-UCCCAAUUCUGGUGA | Cleavage | | 1 |
| Ghi-miR8746 | GhRAD14 | 4.5 | -1 | 1 | 21 | 2260 | 2280 | UCCAUAUUUCACUAUCUCUUA | : :::: :.:::::.:::: | UUAGAGUUGGUGAAGUAUGAU | Cleavage | | 1 |

| | | | | | | | | | | | | | |
|---|---|---|---|---|---|---|---|---|---|---|---|---|---|
| Ghi-miR8746 | GhRAD5 | 4.5 | -1 | 1 | 21 | 2179 | 2199 | UCCAUAUUUCACUAUCUCUUA | : :::: :.:::::.:::: | UUAGAGUUGGUGAAGUAUGAU | Cleavage | | 1 |
| Ghi-miR8752 | GhRAD1 | 4.5 | -1 | 1 | 21 | 108 | 128 | UGAUGGAGAUAGGUAUCUGCA | : :::: :::::: ::::.:. | UUCAGAAACCUAUGUCCGUUC | Cleavage | | 1 |
| Ghi-miRN1385a | GhRAD16 | 4.5 | -1 | 1 | 21 | 1369 | 1389 | CAAAGAGAACAAUACUGGUAU | :.: : :::::.:::::: | UCAUCUGAUUUGUUUUCUUUG | Cleavage | | 1 |
| Ghi-miRN1385b | GhRAD16 | 4.5 | -1 | 1 | 21 | 1369 | 1389 | CAAAGAGAACAAUACUGGUAU | :.: : :::::.:::::: | UCAUCUGAUUUGUUUUCUUUG | Cleavage | | 1 |
| Ghi-miRN1404l | GhRAD8 | 4.5 | -1 | 1 | 20 | 1995 | 2014 | AUGGAGGAGUUGGAAAGAUU | :::::.::.: :::::: | UCUCUUUUCAGCCCCUCCAA | Cleavage | | 1 |
| Ghi-miRN1404l | GhRAD16 | 4.5 | -1 | 1 | 20 | 1995 | 2014 | AUGGAGGAGUUGGAAAGAUU | :::::.::.: :::::: | UCUCUUUUCAGCCCCUCCAA | Cleavage | | 1 |
| Ghi-miRN1404l | GhRAD14 | 4.5 | -1 | 1 | 20 | 730 | 749 | AUGGAGGAGUUGGAAAGAUU | ::.:: :..:.::::: :: | UAUUUUGCUGAUUCCUCAAU | Cleavage | | 1 |
| Ghi-miRN1404l | GhRAD5 | 4.5 | -1 | 1 | 20 | 694 | 713 | AUGGAGGAGUUGGAAAGAUU | ::.:: :..:.::::: :: | UAUUUUGCUGAUUCCUCAAU | Cleavage | | 1 |
| Ghi-miRN1429a | GhRAD7 | 4.5 | -1 | 1 | 21 | 2416 | 2436 | AGUUCCUUCAAAUUCUUCAAC | :::::: :::::::: .:: | CUUGAAGCAUUUGAAGACGCU | Cleavage | | 1 |
| Ghi-miRN1429a | GhRAD17 | 4.5 | -1 | 1 | 21 | 2416 | 2436 | AGUUCCUUCAAAUUCUUCAAC | :::::: :::::::: .:: | CUUGAAGCAUUUGAAGACGCU | Cleavage | | 1 |
| Ghi-miRN1429b | GhRAD7 | 4.5 | -1 | 1 | 21 | 2416 | 2436 | AGUUCCUUCAAAUUCUUCAAC | :::::: :::::::: .:: | CUUGAAGCAUUUGAAGACGCU | Cleavage | | 1 |
| Ghi-miRN1429b | GhRAD17 | 4.5 | -1 | 1 | 21 | 2416 | 2436 | AGUUCCUUCAAAUUCUUCAAC | :::::: :::::::: .:: | CUUGAAGCAUUUGAAGACGCU | Cleavage | | 1 |
| Ghi-miRN1435 | GhRAD6 | 4.5 | -1 | 1 | 21 | 361 | 381 | UUUAGAAAUCAUUCCUUCCUU | ::::::: ::::. ::.::: | AAGGAAGAAAUGGGUUUUAAU | Cleavage | | 1 |
| Ghi-miRN1438 | GhRAD15 | 4.5 | -1 | 1 | 21 | 3115 | 3135 | UGAAUCUAGUUUCUCUCUUAC | .:::::::::::.: :.: | ACGAGAGAGAAACUGGUAUUA | Cleavage | | 3 |
| Ghi-miRN1449a | GhRAD14 | 4.5 | -1 | 1 | 21 | 2379 | 2399 | GGAAGGUUUGGAGGAGAGUGA | .:::.:::...:..: ::: | UGCUUCUUCUCUGGAUUUUCC | Cleavage | | 1 |
| Ghi-miRN1449a | GhRAD5 | 4.5 | -1 | 1 | 21 | 2298 | 2318 | GGAAGGUUUGGAGGAGAGUGA | .:::.:::...:..: ::: | UGCUUCUUCUCUGGAUUUUCC | Cleavage | | 1 |

| | | | | | | | | | | | | | |
|---|---|---|---|---|---|---|---|---|---|---|---|---|---|
| Ghi-miRN1449a | GhRAD8 | 4.5 | -1 | 1 | 21 | 46 | 66 | GGAAGGUUUGGAGGAGAGUGA | :. :::.::: :..::::: | GAAUCCUCUUCCCAGUCUUCC | Cleavage | | 1 |
| Ghi-miRN1463 | GhRAD17 | 4.5 | -1 | 1 | 22 | 261 | 282 | GCUAAAAGUACGGUUCUUUAGC | :::.:..::: ::::::. | GAACAAGGAUUGUAGUUUUAGU | Cleavage | | 1 |
| Ghi-miRN1469a | GhRAD1 | 4.5 | -1 | 1 | 21 | 268 | 288 | CUAGGAGUCAGACUGCAUUUU | .::: ::::.:::::..:: | GAAAGCCAGUUUGACUUUUAU | Cleavage | | 1 |
| Ghi-miRN1469b | GhRAD1 | 4.5 | -1 | 1 | 21 | 268 | 288 | CUAGGAGUCAGACUGCAUUUU | .::: ::::.:::::..:: | GAAAGCCAGUUUGACUUUUAU | Cleavage | | 1 |
| Ghi-miR156q | GhRAD2 | 5 | -1 | 1 | 21 | 502 | 522 | UGACAGAAGAGAGGGAGCACA | : ::::.:::::: :.: | CUUCCUCCUUCUCUUCAAUUA | Cleavage | | 1 |
| Ghi-miR156r | GhRAD2 | 5 | -1 | 1 | 21 | 502 | 522 | UGACAGAAGAGAGGGAGCACA | : ::::.:::::: :.: | CUUCCUCCUUCUCUUCAAUUA | Cleavage | | 1 |
| Ghi-miR159a | GhRAD2 | 5 | -1 | 1 | 21 | 1708 | 1728 | UUUGGAUUGAAGGGAGCUCUA | ::::.::::.: ::::. | UGCUGCUCUCUUCGAGCCAAG | Cleavage | | 1 |
| Ghi-miR159a | GhRAD11 | 5 | -1 | 1 | 21 | 562 | 582 | UUUGGAUUGAAGGGAGCUCUA | ::::.::::.: ::::. | UGCUGCUCUCUUCGAGCCAAG | Cleavage | | 1 |
| Ghi-miR159b | GhRAD2 | 5 | -1 | 1 | 21 | 1708 | 1728 | UUUGGAUUGAAGGGAGCUCUA | ::::.::::.: ::::. | UGCUGCUCUCUUCGAGCCAAG | Cleavage | | 1 |
| Ghi-miR159b | GhRAD11 | 5 | -1 | 1 | 21 | 562 | 582 | UUUGGAUUGAAGGGAGCUCUA | ::::.::::.: ::::. | UGCUGCUCUCUUCGAGCCAAG | Cleavage | | 1 |
| Ghi-miR159c | GhRAD2 | 5 | -1 | 1 | 21 | 1708 | 1728 | UUUGGAUUGAAGGGAGCUCUA | ::::.::::.: ::::. | UGCUGCUCUCUUCGAGCCAAG | Cleavage | | 1 |
| Ghi-miR159c | GhRAD11 | 5 | -1 | 1 | 21 | 562 | 582 | UUUGGAUUGAAGGGAGCUCUA | ::::.::::.: ::::. | UGCUGCUCUCUUCGAGCCAAG | Cleavage | | 1 |
| Ghi-miR159d | GhRAD2 | 5 | -1 | 1 | 21 | 1708 | 1728 | UUUGGAUUGAAGGGAGCUCUA | ::::.::::.: ::::. | UGCUGCUCUCUUCGAGCCAAG | Cleavage | | 1 |
| Ghi-miR159d | GhRAD11 | 5 | -1 | 1 | 21 | 562 | 582 | UUUGGAUUGAAGGGAGCUCUA | ::::.::::.: ::::. | UGCUGCUCUCUUCGAGCCAAG | Cleavage | | 1 |
| Ghi-miR169r | GhRAD10 | 5 | -1 | 1 | 21 | 2414 | 2434 | CAGCCAAGGAUGACUUGCCGA | : :: :. ..::::::::: | UAGGGAGCAUGUCCUUGGCUG | Cleavage | | 1 |
| Ghi-miR169r | GhRAD1 | 5 | -1 | 1 | 21 | 2291 | 2311 | CAGCCAAGGAUGACUUGCCGA | : :: :. ..::::::::: | UAGGGAGCAUGUCCUUGGCUG | Cleavage | | 1 |
| Ghi-miR169s | GhRAD10 | 5 | -1 | 1 | 21 | 2414 | 2434 | CAGCCAAGGAUGACUUGCCGA | : :: :. ..::::::::: | UAGGGAGCAUGUCCUUGGCUG | Cleavage | | 1 |
| Ghi-miR169s | GhRAD1 | 5 | -1 | 1 | 21 | 2291 | 2311 | CAGCCAAGGAUGACUUGCCGA | : :: :. ..::::::::: | UAGGGAGCAUGUCCUUGGCUG | Cleavage | | 1 |
| Ghi-miR169t | GhRAD10 | 5 | -1 | 1 | 21 | 2414 | 2434 | CAGCCAAGGAUGAUUUGCCGG | :: :. ..::::::::: | UAGGGAGCAUGUCCUUGGCUG | Cleavage | | 1 |
| Ghi-miR169t | GhRAD1 | 5 | -1 | 1 | 21 | 2291 | 2311 | CAGCCAAGGAUGAUUUGCCGG | :: :. ..::::::::: | UAGGGAGCAUGUCCUUGGCUG | Cleavage | | 1 |

| | | | | | | | | | | | | | |
|---|---|---|---|---|---|---|---|---|---|---|---|---|---|
| Ghi-miR169u | GhRAD 10 | 5 | -1 | 1 | 21 | 2414 | 2434 | CAGCCAAGGAUGAUUUGCCGG | :: :. ..:::::::::: | UAGGGAGCAUGUCCUUGGCUG | Cleavage | | 1 |
| Ghi-miR169u | GhRAD 1 | 5 | -1 | 1 | 21 | 2291 | 2311 | CAGCCAAGGAUGAUUUGCCGG | :: :. ..:::::::::: | UAGGGAGCAUGUCCUUGGCUG | Cleavage | | 1 |
| Ghi-miR169v | GhRAD 10 | 5 | -1 | 1 | 21 | 2414 | 2434 | CAGCCAAGGAUGACUUGCCGG | :: :. ..:::::::::: | UAGGGAGCAUGUCCUUGGCUG | Cleavage | | 1 |
| Ghi-miR169v | GhRAD 1 | 5 | -1 | 1 | 21 | 2291 | 2311 | CAGCCAAGGAUGACUUGCCGG | :: :. ..:::::::::: | UAGGGAGCAUGUCCUUGGCUG | Cleavage | | 1 |
| Ghi-miR169w | GhRAD 10 | 5 | -1 | 1 | 21 | 2414 | 2434 | CAGCCAAGGAUGACUUGCCGG | :: :. ..:::::::::: | UAGGGAGCAUGUCCUUGGCUG | Cleavage | | 1 |
| Ghi-miR169w | GhRAD 1 | 5 | -1 | 1 | 21 | 2291 | 2311 | CAGCCAAGGAUGACUUGCCGG | :: :. ..:::::::::: | UAGGGAGCAUGUCCUUGGCUG | Cleavage | | 1 |
| Ghi-miR169x | GhRAD 10 | 5 | -1 | 1 | 21 | 2414 | 2434 | CAGCCAAGGAUGACUUGCCGG | :: :. ..:::::::::: | UAGGGAGCAUGUCCUUGGCUG | Cleavage | | 1 |
| Ghi-miR169x | GhRAD 1 | 5 | -1 | 1 | 21 | 2291 | 2311 | CAGCCAAGGAUGACUUGCCGG | :: :. ..:::::::::: | UAGGGAGCAUGUCCUUGGCUG | Cleavage | | 1 |
| Ghi-miR169y | GhRAD 10 | 5 | -1 | 1 | 21 | 2414 | 2434 | CAGCCAAGGAUGACUUGCCGG | :: :. ..:::::::::: | UAGGGAGCAUGUCCUUGGCUG | Cleavage | | 1 |
| Ghi-miR169y | GhRAD 1 | 5 | -1 | 1 | 21 | 2291 | 2311 | CAGCCAAGGAUGACUUGCCGG | :: :. ..:::::::::: | UAGGGAGCAUGUCCUUGGCUG | Cleavage | | 1 |
| Ghi-miR170a | GhRAD 10 | 5 | -1 | 1 | 21 | 863 | 882 | GAGCCGAAUCAAUAUCACUCA | : :::: :::::: :::.:. | UAAGUGAGAUUGAU-CGGUUU | Cleavage | | 1 |
| Ghi-miR170a | GhRAD 1 | 5 | -1 | 1 | 21 | 740 | 759 | GAGCCGAAUCAAUAUCACUCA | : :::: :::::: :::.:. | UAAGUGAGAUUGAU-CGGUUU | Cleavage | | 1 |
| Ghi-miR2111b | GhRAD 13 | 5 | -1 | 1 | 20 | 3514 | 3534 | UAAUCUG-CAUCCUGAGGUUU | ::::::: :::: .:::::: | AAACCUCAAGAUGAUAGAUUA | Cleavage | | 1 |
| Ghi-miR2111b | GhRAD 4 | 5 | -1 | 1 | 20 | 3502 | 3522 | UAAUCUG-CAUCCUGAGGUUU | ::::::: :::: .:::::: | AAACCUCAAGAUGAUAGAUUA | Cleavage | | 1 |
| Ghi-miR2111c | GhRAD 4 | 5 | -1 | 1 | 21 | 3501 | 3522 | UAAUCUG-CAUCCUGAGGUUUA | :::::::: :::: .:::::: | UAAACCUCAAGAUGAUAGAUUA | Cleavage | | 1 |
| Ghi-miR2111c | GhRAD 13 | 5 | -1 | 1 | 21 | 3513 | 3534 | UAAUCUG-CAUCCUGAGGUUUA | :::::::: :::: .:::::: | UAAACCUCAAGAUGAUAGAUUA | Cleavage | | 1 |
| Ghi-miR2111d | GhRAD 13 | 5 | -1 | 1 | 21 | 3513 | 3534 | UAAUCUG-CAUCCUGAGGUUUA | :::::::: :::: .:::::: | UAAACCUCAAGAUGAUAGAUUA | Cleavage | | 1 |
| Ghi-miR2111d | GhRAD 4 | 5 | -1 | 1 | 21 | 3501 | 3522 | UAAUCUG-CAUCCUGAGGUUUA | :::::::: :::: .:::::: | UAAACCUCAAGAUGAUAGAUUA | Cleavage | | 1 |
| Ghi-miR2949a | GhRAD 14 | 5 | -1 | 1 | 22 | 3344 | 3365 | ACUUUUGAACUGGAUUUGCCGA | :.: . . ::..:::::::::: | UUGAUUGUUCUGGUUCAAAAGU | Cleavage | | 1 |
| Ghi-miR2949a | GhRAD 5 | 5 | -1 | 1 | 22 | 3263 | 3284 | ACUUUUGAACUGGAUUUGCCGA | :.: . . ::..:::::::::: | UUGAUUGUUCUGGUUCAAAAGU | Cleavage | | 1 |
| Ghi-miR2949b | GhRAD 14 | 5 | -1 | 1 | 22 | 3344 | 3365 | ACUUUUGAACUGGAUUUGCCGA | :.: . . ::..:::::::::: | UUGAUUGUUCUGGUUCAAAAGU | Cleavage | | 1 |
| Ghi-miR2949b | GhRAD 5 | 5 | -1 | 1 | 22 | 3263 | 3284 | ACUUUUGAACUGGAUUUGCCGA | :.: . . ::..:::::::::: | UUGAUUGUUCUGGUUCAAAAGU | Cleavage | | 1 |
| Ghi-miR2950a | GhRAD 9 | 5 | -1 | 1 | 21 | 229 | 249 | UUCCAUCUCUUGCACACUGGA | ..::: ::.:::: :::. | AUUAGUAAGCGAGAGAAGGAG | Cleavage | | 1 |

| | | | | | | | | | | | | | |
|---|---|---|---|---|---|---|---|---|---|---|---|---|---|
| Ghi-miR2950b | GhRAD9 | 5 | -1 | 1 | 21 | 229 | 249 | UUCCAUCUCUUGCACACUGGA | ..::: ::.::::: :::. | AUUAGUAAGCGAGAGAAGGAG | Cleavage | | 1 |
| Ghi-miR2950c | GhRAD9 | 5 | -1 | 1 | 21 | 229 | 249 | UUCCAUCUCUUGCACACUGGA | ..::: ::.::::: :::. | AUUAGUAAGCGAGAGAAGGAG | Cleavage | | 1 |
| Ghi-miR2950d | GhRAD9 | 5 | -1 | 1 | 21 | 229 | 249 | UUCCAUCUCUUGCACACUGGA | ..::: ::.::::: :::. | AUUAGUAAGCGAGAGAAGGAG | Cleavage | | 1 |
| Ghi-miR3476d | GhRAD14 | 5 | -1 | 1 | 21 | 574 | 594 | UGAACUGGGUUUGUUGGCUGC | :..: ..:.::: :.::::: | GUGGAUGAUAAAACUAGUUCA | Cleavage | | 1 |
| Ghi-miR3476e | GhRAD14 | 5 | -1 | 1 | 21 | 574 | 594 | UGAACUGGGUUUGUUGGCUGC | :..: ..:.::: :.::::: | GUGGAUGAUAAAACUAGUUCA | Cleavage | | 1 |
| Ghi-miR3627a | GhRAD13 | 5 | -1 | 1 | 22 | 1833 | 1854 | UUGUCGCAGGAGAGAUGGCACU | .: ::::.:::::::: | AACAGUACCUCUUCUGCGACAU | Cleavage | | 1 |
| Ghi-miR3627a | GhRAD4 | 5 | -1 | 1 | 22 | 1827 | 1848 | UUGUCGCAGGAGAGAUGGCACU | .: ::::.:::::::: | AACAGUACCUCUUCUGCGACAU | Cleavage | | 1 |
| Ghi-miR3627b | GhRAD13 | 5 | -1 | 1 | 22 | 1833 | 1854 | UUGUCGCAGGAGAGAUGGCACU | .: ::::.:::::::: | AACAGUACCUCUUCUGCGACAU | Cleavage | | 1 |
| Ghi-miR3627b | GhRAD4 | 5 | -1 | 1 | 22 | 1827 | 1848 | UUGUCGCAGGAGAGAUGGCACU | .: ::::.:::::::: | AACAGUACCUCUUCUGCGACAU | Cleavage | | 1 |
| Ghi-miR390a | GhRAD11 | 5 | -1 | 1 | 21 | 1804 | 1824 | AAGCUCAGGAGGGAUAGCGCC | .:.:::::.::. :: ::: | CAUGUUAUCCUUCUGGACCUU | Cleavage | | 1 |
| Ghi-miR390a | GhRAD10 | 5 | -1 | 1 | 21 | 2478 | 2498 | AAGCUCAGGAGGGAUAGCGCC | : :::..::: ::::.:: | CAAGAUAUUUCUCAUGAGUUU | Cleavage | | 1 |
| Ghi-miR390a | GhRAD1 | 5 | -1 | 1 | 21 | 2355 | 2375 | AAGCUCAGGAGGGAUAGCGCC | : :::..::: ::::.:: | CAAGAUAUUUCUCAUGAGUUU | Cleavage | | 1 |
| Ghi-miR390b | GhRAD11 | 5 | -1 | 1 | 21 | 1804 | 1824 | AAGCUCAGGAGGGAUAGCGCC | .:.:::::.::. :: ::: | CAUGUUAUCCUUCUGGACCUU | Cleavage | | 1 |
| Ghi-miR390b | GhRAD10 | 5 | -1 | 1 | 21 | 2478 | 2498 | AAGCUCAGGAGGGAUAGCGCC | : :::..::: ::::.:: | CAAGAUAUUUCUCAUGAGUUU | Cleavage | | 1 |
| Ghi-miR390b | GhRAD1 | 5 | -1 | 1 | 21 | 2355 | 2375 | AAGCUCAGGAGGGAUAGCGCC | : :::..::: ::::.:: | CAAGAUAUUUCUCAUGAGUUU | Cleavage | | 1 |
| Ghi-miR390c | GhRAD11 | 5 | -1 | 1 | 21 | 1804 | 1824 | AAGCUCAGGAGGGAUAGCGCC | .:.:::::.::. :: ::: | CAUGUUAUCCUUCUGGACCUU | Cleavage | | 1 |
| Ghi-miR390c | GhRAD10 | 5 | -1 | 1 | 21 | 2478 | 2498 | AAGCUCAGGAGGGAUAGCGCC | : :::..::: ::::.:: | CAAGAUAUUUCUCAUGAGUUU | Cleavage | | 1 |
| Ghi-miR390c | GhRAD1 | 5 | -1 | 1 | 21 | 2355 | 2375 | AAGCUCAGGAGGGAUAGCGCC | : :::..::: ::::.:: | CAAGAUAUUUCUCAUGAGUUU | Cleavage | | 1 |
| Ghi-miR390d | GhRAD11 | 5 | -1 | 1 | 21 | 1804 | 1824 | AAGCUCAGGAGGGAUAGCGCC | .:.:::::.::. :: ::: | CAUGUUAUCCUUCUGGACCUU | Cleavage | | 1 |
| Ghi-miR390d | GhRAD10 | 5 | -1 | 1 | 21 | 2478 | 2498 | AAGCUCAGGAGGGAUAGCGCC | : :::..::: ::::.:: | CAAGAUAUUUCUCAUGAGUUU | Cleavage | | 1 |
| Ghi-miR390d | GhRAD1 | 5 | -1 | 1 | 21 | 2355 | 2375 | AAGCUCAGGAGGGAUAGCGCC | : :::..::: ::::.:: | CAAGAUAUUUCUCAUGAGUUU | Cleavage | | 1 |
| Ghi-miR390e | GhRAD11 | 5 | -1 | 1 | 21 | 1804 | 1824 | AAGCUCAGGAGGGAUAGCGCC | .:.:::::.::. :: ::: | CAUGUUAUCCUUCUGGACCUU | Cleavage | | 1 |
| Ghi-miR390e | GhRAD10 | 5 | -1 | 1 | 21 | 2478 | 2498 | AAGCUCAGGAGGGAUAGCGCC | : :::..::: ::::.:: | CAAGAUAUUUCUCAUGAGUUU | Cleavage | | 1 |

| | | | | | | | | | | | | | |
|---|---|---|---|---|---|---|---|---|---|---|---|---|---|
| Ghi-miR390e | GhRAD 1 | 5 | -1 | 1 | 21 | 2355 | 2375 | AAGCUCAGGAGGGAUAGCGCC | : :::..::: ::::.:: | CAAGAUAUUUCUCAUGAGUUU | Cleavage | | 1 |
| Ghi-miR390f | GhRAD 11 | 5 | -1 | 1 | 21 | 1804 | 1824 | AAGCUCAGGAGGGAUAGCGCC | .:.:::::.::. :: ::: | CAUGUUAUCCUUCUGGACCUU | Cleavage | | 1 |
| Ghi-miR390f | GhRAD 10 | 5 | -1 | 1 | 21 | 2478 | 2498 | AAGCUCAGGAGGGAUAGCGCC | : :::..::: ::::.:: | CAAGAUAUUUCUCAUGAGUUU | Cleavage | | 1 |
| Ghi-miR390f | GhRAD 1 | 5 | -1 | 1 | 21 | 2355 | 2375 | AAGCUCAGGAGGGAUAGCGCC | : :::..::: ::::.:: | CAAGAUAUUUCUCAUGAGUUU | Cleavage | | 1 |
| Ghi-miR390g | GhRAD 11 | 5 | -1 | 1 | 21 | 1804 | 1824 | AAGCUCAGGAGGGAUAGCGCC | .:.:::::.::. :: ::: | CAUGUUAUCCUUCUGGACCUU | Cleavage | | 1 |
| Ghi-miR390g | GhRAD 10 | 5 | -1 | 1 | 21 | 2478 | 2498 | AAGCUCAGGAGGGAUAGCGCC | : :::..::: ::::.:: | CAAGAUAUUUCUCAUGAGUUU | Cleavage | | 1 |
| Ghi-miR390g | GhRAD 1 | 5 | -1 | 1 | 21 | 2355 | 2375 | AAGCUCAGGAGGGAUAGCGCC | : :::..::: ::::.:: | CAAGAUAUUUCUCAUGAGUUU | Cleavage | | 1 |
| Ghi-miR390h | GhRAD 11 | 5 | -1 | 1 | 21 | 1804 | 1824 | AAGCUCAGGAGGGAUAGCGCC | .:.:::::.::. :: ::: | CAUGUUAUCCUUCUGGACCUU | Cleavage | | 1 |
| Ghi-miR390h | GhRAD 10 | 5 | -1 | 1 | 21 | 2478 | 2498 | AAGCUCAGGAGGGAUAGCGCC | : :::..::: ::::.:: | CAAGAUAUUUCUCAUGAGUUU | Cleavage | | 1 |
| Ghi-miR390h | GhRAD 1 | 5 | -1 | 1 | 21 | 2355 | 2375 | AAGCUCAGGAGGGAUAGCGCC | : :::..::: ::::.:: | CAAGAUAUUUCUCAUGAGUUU | Cleavage | | 1 |
| Ghi-miR390i | GhRAD 11 | 5 | -1 | 1 | 21 | 1804 | 1824 | AAGCUCAGGAGGGAUAGCGCC | .:.:::::.::. :: ::: | CAUGUUAUCCUUCUGGACCUU | Cleavage | | 1 |
| Ghi-miR390i | GhRAD 10 | 5 | -1 | 1 | 21 | 2478 | 2498 | AAGCUCAGGAGGGAUAGCGCC | : :::..::: ::::.:: | CAAGAUAUUUCUCAUGAGUUU | Cleavage | | 1 |
| Ghi-miR390i | GhRAD 1 | 5 | -1 | 1 | 21 | 2355 | 2375 | AAGCUCAGGAGGGAUAGCGCC | : :::..::: ::::.:: | CAAGAUAUUUCUCAUGAGUUU | Cleavage | | 1 |
| Ghi-miR397c | GhRAD 14 | 5 | -1 | 1 | 21 | 3417 | 3437 | UCAUUGAGUGCAGCGUUGAUG | ::.:: ::::: ::..::: | AAUUAAAGCUGCCCUUGAUGU | Cleavage | | 1 |
| Ghi-miR397d | GhRAD 14 | 5 | -1 | 1 | 21 | 3417 | 3437 | UCAUUGAGUGCAGCGUUGAUG | ::.:: ::::: ::..::: | AAUUAAAGCUGCCCUUGAUGU | Cleavage | | 1 |
| Ghi-miR397e | GhRAD 14 | 5 | -1 | 1 | 21 | 3417 | 3437 | UCAUUGAGUGCAGCGUUGAUG | ::.:: ::::: ::..::: | AAUUAAAGCUGCCCUUGAUGU | Cleavage | | 1 |
| Ghi-miR398a | GhRAD 15 | 5 | -1 | 1 | 21 | 1844 | 1864 | UGUGUUCUCAGGUCGCCCCUG | :::.::.:: :::.::.. | CCAGGGUGAUCUCAGAGCAUG | Cleavage | | 1 |
| Ghi-miR398a | GhRAD 6 | 5 | -1 | 1 | 21 | 1844 | 1864 | UGUGUUCUCAGGUCGCCCCUG | :::.::.:: :::.::.. | CCAGGGUGAUCUCAGAGCAUG | Cleavage | | 1 |
| Ghi-miR398b | GhRAD 15 | 5 | -1 | 1 | 21 | 1844 | 1864 | UGUGUUCUCAGGUCGCCCCUG | :::.::.:: :::.::.. | CCAGGGUGAUCUCAGAGCAUG | Cleavage | | 1 |
| Ghi-miR398b | GhRAD 6 | 5 | -1 | 1 | 21 | 1844 | 1864 | UGUGUUCUCAGGUCGCCCCUG | :::.::.:: :::.::.. | CCAGGGUGAUCUCAGAGCAUG | Cleavage | | 1 |
| Ghi-miR398c | GhRAD 9 | 5 | -1 | 1 | 21 | 505 | 525 | UGUGUUCUCAGGUCACCCCUU | .: :: : ..:::.::::::. | GAUGGUUUGUCUGGGAACACG | Cleavage | | 1 |
| Ghi-miR398d | GhRAD 9 | 5 | -1 | 1 | 21 | 505 | 525 | UGUGUUCUCAGGUCACCCCUU | .: :: : ..:::.::::::. | GAUGGUUUGUCUGGGAACACG | Cleavage | | 1 |
| Ghi-miR482e | GhRAD 14 | 5 | -1 | 1 | 22 | 1903 | 1924 | UCUUUCCUACUCCUCCCAUUCC | :: .::: :::::::: | UUCUUGCAGGGAUUAGGAAAGA | Translation | | 1 |

| | | | | | | | | | | | | | |
|---|---|---|---|---|---|---|---|---|---|---|---|---|---|
| Ghi-miR535a | GhRAD8 | 5 | -1 | 1 | 21 | 437 | 456 | UGACAACGAGAGAGAGCACGU | :.::: :::.:: ::::::: | AUGUG-UCUUUCACGUUGUCU | Cleavage | | 1 |
| Ghi-miR535a | GhRAD16 | 5 | -1 | 1 | 21 | 437 | 456 | UGACAACGAGAGAGAGCACGU | :.::: :::.:: ::::::: | AUGUG-UCUUUCACGUUGUCU | Cleavage | | 1 |
| Ghi-miR535d | GhRAD8 | 5 | -1 | 1 | 21 | 437 | 456 | UGACAACGAGAGAGAGCACGU | :.::: :::.:: ::::::: | AUGUG-UCUUUCACGUUGUCU | Cleavage | | 1 |
| Ghi-miR535d | GhRAD16 | 5 | -1 | 1 | 21 | 437 | 456 | UGACAACGAGAGAGAGCACGU | :.::: :::.:: ::::::: | AUGUG-UCUUUCACGUUGUCU | Cleavage | | 1 |
| Ghi-miR7484o | GhRAD13 | 5 | -1 | 1 | 21 | 3723 | 3743 | UUUGUAUGUUAGAUCGAAGAG | .::::: ::: ::.::: ::. | UUCUUCCAUCCAAUAUAGAAG | Translation | | 1 |
| Ghi-miR7484o | GhRAD4 | 5 | -1 | 1 | 21 | 3711 | 3731 | UUUGUAUGUUAGAUCGAAGAG | .::::: ::: ::.::: ::. | UUCUUCCAUCCAAUAUAGAAG | Translation | | 1 |
| Ghi-miR7484w | GhRAD4 | 5 | -1 | 1 | 21 | 2745 | 2765 | UUAAUGGUAGAAAUGGAUGAA | ::::.: ::::.:::: :: | GUCAUUCUGUUCUGCCAUAAA | Cleavage | | 1 |
| Ghi-miR7495 | GhRAD12 | 5 | -1 | 1 | 21 | 2556 | 2575 | UUACUUUAGAUGUCUCCUUCA | :::: :::::::.::::: :: | UGAAC-AGACAUUUAAAGAAA | Cleavage | | 1 |
| Ghi-miR7495 | GhRAD3 | 5 | -1 | 1 | 21 | 2550 | 2569 | UUACUUUAGAUGUCUCCUUCA | :::: :::::::.::::: :: | UGAAC-AGACAUUUAAAGAAA | Cleavage | | 1 |
| Ghi-miR7508a | GhRAD16 | 5 | -1 | 1 | 21 | 1368 | 1388 | CAAGAAAAGAAGUCGGGAGAG | ::: .:.::.:: ::::::: | CUCAUCUGAUUUGUUUUCUUU | Cleavage | | 1 |
| Ghi-miR7508b | GhRAD16 | 5 | -1 | 1 | 21 | 1368 | 1388 | CAAGAAAAGAAGUCGGGAGAG | ::: .:.::.:: ::::::: | CUCAUCUGAUUUGUUUUCUUU | Cleavage | | 1 |
| Ghi-miR7513 | GhRAD13 | 5 | -1 | 1 | 21 | 1295 | 1315 | AAUCAGCCAGGAAUCGUUUGA | .::.: .:::.:: ::::: | CUAAGCAGUUCUUGUCUGAUG | Cleavage | | 1 |
| Ghi-miR828a | GhRAD7 | 5 | -1 | 1 | 22 | 1925 | 1946 | UCUUGCUCAAAUGAGUAUUCCA | :: : :::::::: :::. | UCCAAAAACCAUUUGAGGAAGG | Cleavage | | 1 |
| Ghi-miR828a | GhRAD17 | 5 | -1 | 1 | 22 | 1925 | 1946 | UCUUGCUCAAAUGAGUAUUCCA | :: : :::::::: :::. | UUCAAAAACCAUUUGAGGAAGG | Cleavage | | 1 |
| Ghi-miR828b | GhRAD7 | 5 | -1 | 1 | 22 | 1925 | 1946 | UCUUGCUCAAAUGAGUAUUCCA | :: : :::::::: :::. | UCCAAAAACCAUUUGAGGAAGG | Cleavage | | 1 |
| Ghi-miR828b | GhRAD17 | 5 | -1 | 1 | 22 | 1925 | 1946 | UCUUGCUCAAAUGAGUAUUCCA | :: : :::::::: :::. | UUCAAAAACCAUUUGAGGAAGG | Cleavage | | 1 |
| Ghi-miR828c | GhRAD7 | 5 | -1 | 1 | 22 | 1925 | 1946 | UCUUGCUCAAAUGAGUAUUCCA | :: : :::::::: :::. | UCCAAAAACCAUUUGAGGAAGG | Cleavage | | 1 |
| Ghi-miR828c | GhRAD17 | 5 | -1 | 1 | 22 | 1925 | 1946 | UCUUGCUCAAAUGAGUAUUCCA | :: : :::::::: :::. | UUCAAAAACCAUUUGAGGAAGG | Cleavage | | 1 |
| Ghi-miR828d | GhRAD7 | 5 | -1 | 1 | 22 | 1925 | 1946 | UCUUGCUCAAAUGAGUAUUCCA | :: : :::::::: :::. | UCCAAAAACCAUUUGAGGAAGG | Cleavage | | 1 |
| Ghi-miR828d | GhRAD17 | 5 | -1 | 1 | 22 | 1925 | 1946 | UCUUGCUCAAAUGAGUAUUCCA | :: : :::::::: :::. | UUCAAAAACCAUUUGAGGAAGG | Cleavage | | 1 |
| Ghi-miR8632a | GhRAD7 | 5 | -1 | 1 | 21 | 2492 | 2512 | AUGAGCUAGAAGUUGGAACUC | ::::: ::::.:.:.:: | GGAAUCCAAAUUCUGGUUUAU | Cleavage | | 1 |

| | | | | | | | | | | | | | | |
|---|---|---|---|---|---|---|---|---|---|---|---|---|---|---|
| Ghi-miR8632a | GhRAD17 | 5 | -1 | 1 | 21 | 2492 | 2512 | AUGAGCUAGAAGUUGGAACUC | ::::: ::::.:.:.:: | GGAAUCCAAAUUCUGGUUUAU | Cleavage | | | 1 |
| Ghi-miR8632b | GhRAD7 | 5 | -1 | 1 | 21 | 2492 | 2512 | AUGAGCUAGAAGUUGGAACUC | ::::: ::::.:.:.:: | GGAAUCCAAAUUCUGGUUUAU | Cleavage | | | 1 |
| Ghi-miR8632b | GhRAD17 | 5 | -1 | 1 | 21 | 2492 | 2512 | AUGAGCUAGAAGUUGGAACUC | ::::: ::::.:.:.:: | GGAAUCCAAAUUCUGGUUUAU | Cleavage | | | 1 |
| Ghi-miR8632c | GhRAD7 | 5 | -1 | 1 | 21 | 2492 | 2512 | AUGAGCUAGAAGUUGGAACUC | ::::: ::::.:.:.:: | GGAAUCCAAAUUCUGGUUUAU | Cleavage | | | 1 |
| Ghi-miR8632c | GhRAD17 | 5 | -1 | 1 | 21 | 2492 | 2512 | AUGAGCUAGAAGUUGGAACUC | ::::: ::::.:.:.:: | GGAAUCCAAAUUCUGGUUUAU | Cleavage | | | 1 |
| Ghi-miR8638 | GhRAD7 | 5 | -1 | 1 | 21 | 1388 | 1408 | UGAAUACAGGAAUGGCUCUCU | ::::::: :::::::: | GUGAAGCCAUUGAUGUAUUCA | Translation | | | 1 |
| Ghi-miR8646 | GhRAD17 | 5 | -1 | 1 | 21 | 1439 | 1459 | UAGUGAGGAUGGGAAAUUUGU | ::.:::::: :.:.. :: | CGAAGUUUCCCAGCUUUGAUA | Cleavage | | | 1 |
| Ghi-miR8677 | GhRAD6 | 5 | -1 | 1 | 21 | 2995 | 3015 | AAUGAAUCUAGUUUCUCUCUU | :.:: ::.::.:.:::: : | AGGAAAGGAAUUGGAUUCUUA | Cleavage | | | 2 |
| Ghi-miR8677 | GhRAD15 | 5 | -1 | 1 | 21 | 3034 | 3054 | AAUGAAUCUAGUUUCUCUCUU | :.:: ::.::.:.:::: : | AGGAAAGGAAUUGGAUUCUUA | Cleavage | | | 2 |
| Ghi-miR8677 | GhRAD5 | 5 | -1 | 1 | 21 | 1390 | 1410 | AAUGAAUCUAGUUUCUCUCUU | .: :::::.: :: .:::::: | GAAAGAGAGAAUAAGUUCAUU | Translation | | | 1 |
| Ghi-miR8764b | GhRAD13 | 5 | -1 | 1 | 22 | 3997 | 4018 | AAGGGGGUAAAAUGCAAUCUAA | :::: :.:::::: :.:.: | GAAGAUCGUAUUUUAGCUCUUC | Cleavage | | | 1 |
| Ghi-miR8764b | GhRAD4 | 5 | -1 | 1 | 22 | 3985 | 4006 | AAGGGGGUAAAAUGCAAUCUAA | :::: :.:::::: :.:.: | GAAGAUCGUAUUUUAGCUCUUC | Cleavage | | | 1 |
| Ghi-miRN1385a | GhRAD8 | 5 | -1 | 1 | 21 | 1369 | 1389 | CAAAGAGAACAAUACUGGUAU | :.. : :::::.:::::: | UCAUUUGAUUUGUUUUCUUUG | Cleavage | | | 1 |
| Ghi-miRN1385b | GhRAD8 | 5 | -1 | 1 | 21 | 1369 | 1389 | CAAAGAGAACAAUACUGGUAU | :.. : :::::.:::::: | UCAUUUGAUUUGUUUUCUUUG | Cleavage | | | 1 |
| Ghi-miRN1393a | GhRAD4 | 5 | -1 | 1 | 22 | 1016 | 1037 | GCAUCAGAGGAGUCAAGCAGGU | .:: :::.:.::: ::::. | CAUUGACUGAUUUCUCAGAUGU | Cleavage | | | 1 |
| Ghi-miRN1393a | GhRAD13 | 5 | -1 | 1 | 22 | 1022 | 1043 | GCAUCAGAGGAGUCAAGCAGGU | .:: :::.:.::: ::::. | CAUUGACUGAUUUCUCAGAUGU | Cleavage | | | 1 |
| Ghi-miRN1393b | GhRAD4 | 5 | -1 | 1 | 22 | 1016 | 1037 | GCAUCAGAGGAGUCAAGCAGGU | .:: :::.:.::: ::::. | CAUUGACUGAUUUCUCAGAUGU | Cleavage | | | 1 |
| Ghi-miRN1393b | GhRAD13 | 5 | -1 | 1 | 22 | 1022 | 1043 | GCAUCAGAGGAGUCAAGCAGGU | .:: :::.:.::: ::::. | CAUUGACUGAUUUCUCAGAUGU | Cleavage | | | 1 |
| Ghi-miRN1393c | GhRAD4 | 5 | -1 | 1 | 22 | 1016 | 1037 | GCAUCAGAGGAGUCAAGCAGGU | .:: :::.:.::: ::::. | CAUUGACUGAUUUCUCAGAUGU | Cleavage | | | 1 |

| | | | | | | | | | | | | |
|---|---|---|---|---|---|---|---|---|---|---|---|---|
| Ghi-miRN1393c | GhRAD13 | 5 | -1 | 1 | 22 | 1022 | 1043 | GCAUCAGAGGAGUCAAGCAGGU | .:: :::.:.::: ::::. | CAUUGACUGAUUUCUCAGAUGU | Cleavage | | 1 |
| Ghi-miRN1393d | GhRAD4 | 5 | -1 | 1 | 22 | 1016 | 1037 | GCAUCAGAGGAGUCAAGCAGGU | .:: :::.:.::: ::::. | CAUUGACUGAUUUCUCAGAUGU | Cleavage | | 1 |
| Ghi-miRN1393d | GhRAD13 | 5 | -1 | 1 | 22 | 1022 | 1043 | GCAUCAGAGGAGUCAAGCAGGU | .:: :::.:.::: ::::. | CAUUGACUGAUUUCUCAGAUGU | Cleavage | | 1 |
| Ghi-miRN1403a | GhRAD13 | 5 | -1 | 1 | 21 | 3722 | 3742 | UUAUAUAUUAGAUCAAAGAGC | :.:::: ::: :::::: :: | GUUCUUCCAUCCAAUAUAGAA | Translation | | 1 |
| Ghi-miRN1403a | GhRAD4 | 5 | -1 | 1 | 21 | 3710 | 3730 | UUAUAUAUUAGAUCAAAGAGC | :.:::: ::: :::::: :: | GUUCUUCCAUCCAAUAUAGAA | Translation | | 1 |
| Ghi-miRN1403b | GhRAD13 | 5 | -1 | 1 | 21 | 3722 | 3742 | UUAUAUAUUAGAUCAAAGAGC | :.:::: ::: :::::: :: | GUUCUUCCAUCCAAUAUAGAA | Translation | | 1 |
| Ghi-miRN1403b | GhRAD4 | 5 | -1 | 1 | 21 | 3710 | 3730 | UUAUAUAUUAGAUCAAAGAGC | :.:::: ::: :::::: :: | GUUCUUCCAUCCAAUAUAGAA | Translation | | 1 |
| Ghi-miRN1403c | GhRAD13 | 5 | -1 | 1 | 21 | 3722 | 3742 | UUAUAUAUUAGAUCAAAGAGC | :.:::: ::: :::::: :: | GUUCUUCCAUCCAAUAUAGAA | Translation | | 1 |
| Ghi-miRN1403c | GhRAD4 | 5 | -1 | 1 | 21 | 3710 | 3730 | UUAUAUAUUAGAUCAAAGAGC | :.:::: ::: :::::: :: | GUUCUUCCAUCCAAUAUAGAA | Translation | | 1 |
| Ghi-miRN1403d | GhRAD13 | 5 | -1 | 1 | 21 | 3722 | 3742 | UUAUAUAUUAGAUCAAAGAGC | :.:::: ::: :::::: :: | GUUCUUCCAUCCAAUAUAGAA | Translation | | 1 |
| Ghi-miRN1403d | GhRAD4 | 5 | -1 | 1 | 21 | 3710 | 3730 | UUAUAUAUUAGAUCAAAGAGC | :.:::: ::: :::::: :: | GUUCUUCCAUCCAAUAUAGAA | Translation | | 1 |
| Ghi-miRN1403e | GhRAD13 | 5 | -1 | 1 | 21 | 3722 | 3742 | UUAUAUAUUAGAUCAAAGAGC | :.:::: ::: :::::: :: | GUUCUUCCAUCCAAUAUAGAA | Translation | | 1 |
| Ghi-miRN1403e | GhRAD4 | 5 | -1 | 1 | 21 | 3710 | 3730 | UUAUAUAUUAGAUCAAAGAGC | :.:::: ::: :::::: :: | GUUCUUCCAUCCAAUAUAGAA | Translation | | 1 |
| Ghi-miRN1403f | GhRAD13 | 5 | -1 | 1 | 21 | 3722 | 3742 | UUAUAUAUUAGAUCAAAGAGC | :.:::: ::: :::::: :: | GUUCUUCCAUCCAAUAUAGAA | Translation | | 1 |
| Ghi-miRN1403f | GhRAD4 | 5 | -1 | 1 | 21 | 3710 | 3730 | UUAUAUAUUAGAUCAAAGAGC | :.:::: ::: :::::: :: | GUUCUUCCAUCCAAUAUAGAA | Translation | | 1 |

| | | | | | | | | | | | | | | | |
|---|---|---|---|---|---|---|---|---|---|---|---|---|---|---|---|
| Ghi-miRN1403g | GhRAD13 | 5 | -1 | 1 | 21 | 3722 | 3742 | UUAUAUAUUAGAUCAAAGAGC | :.:::: ::: :::::: :: | GUUCUUCCAUCCAAUAUAGAA | Translation | | 1 | | |
| Ghi-miRN1403g | GhRAD4 | 5 | -1 | 1 | 21 | 3710 | 3730 | UUAUAUAUUAGAUCAAAGAGC | :.:::: ::: :::::: :: | GUUCUUCCAUCCAAUAUAGAA | Translation | | 1 | | |
| Ghi-miRN1403h | GhRAD13 | 5 | -1 | 1 | 21 | 3722 | 3742 | UUAUAUAUUAGAUCAAAGAGC | :.:::: ::: :::::: :: | GUUCUUCCAUCCAAUAUAGAA | Translation | | 1 | | |
| Ghi-miRN1403h | GhRAD4 | 5 | -1 | 1 | 21 | 3710 | 3730 | UUAUAUAUUAGAUCAAAGAGC | :.:::: ::: :::::: :: | GUUCUUCCAUCCAAUAUAGAA | Translation | | 1 | | |
| Ghi-miRN1404a | GhRAD8 | 5 | -1 | 1 | 21 | 1995 | 2015 | AAUGGAGGAGUUGGAAAGAUU | :::::.::.: :::::: | UCUCUUUUCAGCCCCUCCAAA | Cleavage | | 1 | | |
| Ghi-miRN1404a | GhRAD16 | 5 | -1 | 1 | 21 | 1995 | 2015 | AAUGGAGGAGUUGGAAAGAUU | :::::.::.: :::::: | UCUCUUUUCAGCCCCUCCAAA | Cleavage | | 1 | | |
| Ghi-miRN1404b | GhRAD8 | 5 | -1 | 1 | 21 | 1995 | 2015 | AAUGGAGGAGUUGGAAAGAUU | :::::.::.: :::::: | UCUCUUUUCAGCCCCUCCAAA | Cleavage | | 1 | | |
| Ghi-miRN1404b | GhRAD16 | 5 | -1 | 1 | 21 | 1995 | 2015 | AAUGGAGGAGUUGGAAAGAUU | :::::.::.: :::::: | UCUCUUUUCAGCCCCUCCAAA | Cleavage | | 1 | | |
| Ghi-miRN1404c | GhRAD8 | 5 | -1 | 1 | 21 | 1995 | 2015 | AAUGGAGGAGUUGGAAAGAUU | :::::.::.: :::::: | UCUCUUUUCAGCCCCUCCAAA | Cleavage | | 1 | | |
| Ghi-miRN1404c | GhRAD16 | 5 | -1 | 1 | 21 | 1995 | 2015 | AAUGGAGGAGUUGGAAAGAUU | :::::.::.: :::::: | UCUCUUUUCAGCCCCUCCAAA | Cleavage | | 1 | | |
| Ghi-miRN1404d | GhRAD8 | 5 | -1 | 1 | 21 | 1995 | 2015 | AAUGGAGGAGUUGGAAAGAUU | :::::.::.: :::::: | UCUCUUUUCAGCCCCUCCAAA | Cleavage | | 1 | | |
| Ghi-miRN1404d | GhRAD16 | 5 | -1 | 1 | 21 | 1995 | 2015 | AAUGGAGGAGUUGGAAAGAUU | :::::.::.: :::::: | UCUCUUUUCAGCCCCUCCAAA | Cleavage | | 1 | | |
| Ghi-miRN1404e | GhRAD8 | 5 | -1 | 1 | 21 | 1995 | 2015 | AAUGGAGGAGUUGGAAAGAUU | :::::.::.: :::::: | UCUCUUUUCAGCCCCUCCAAA | Cleavage | | 1 | | |
| Ghi-miRN1404e | GhRAD16 | 5 | -1 | 1 | 21 | 1995 | 2015 | AAUGGAGGAGUUGGAAAGAUU | :::::.::.: :::::: | UCUCUUUUCAGCCCCUCCAAA | Cleavage | | 1 | | |
| Ghi-miRN1404f | GhRAD8 | 5 | -1 | 1 | 21 | 1995 | 2015 | AAUGGAGGAGUUGGAAAGAUU | :::::.::.: :::::: | UCUCUUUUCAGCCCCUCCAAA | Cleavage | | 1 | | |

| | | | | | | | | | | | | | |
|---|---|---|---|---|---|---|---|---|---|---|---|---|---|
| Ghi-miRN1404f | GhRAD16 | 5 | -1 | 1 | 21 | 1995 | 2015 | AAUGGAGGAGUUGGAAAGAUU | :::::.::.: :::::: | UCUCUUUUCAGCCCCUCCAA | Cleavage | | 1 |
| Ghi-miRN1404g | GhRAD8 | 5 | -1 | 1 | 21 | 1995 | 2015 | AAUGGAGGAGUUGGAAAGAUU | :::::.::.: :::::: | UCUCUUUUCAGCCCCUCCAA | Cleavage | | 1 |
| Ghi-miRN1404g | GhRAD16 | 5 | -1 | 1 | 21 | 1995 | 2015 | AAUGGAGGAGUUGGAAAGAUU | :::::.::.: :::::: | UCUCUUUUCAGCCCCUCCAA | Cleavage | | 1 |
| Ghi-miRN1404h | GhRAD8 | 5 | -1 | 1 | 21 | 1995 | 2015 | AAUGGAGGAGUUGGAAAGAUU | :::::.::.: :::::: | UCUCUUUUCAGCCCCUCCAA | Cleavage | | 1 |
| Ghi-miRN1404h | GhRAD16 | 5 | -1 | 1 | 21 | 1995 | 2015 | AAUGGAGGAGUUGGAAAGAUU | :::::.::.: :::::: | UCUCUUUUCAGCCCCUCCAA | Cleavage | | 1 |
| Ghi-miRN1404i | GhRAD8 | 5 | -1 | 1 | 21 | 1995 | 2015 | AAUGGAGGAGUUGGAAAGAUU | :::::.::.: :::::: | UCUCUUUUCAGCCCCUCCAA | Cleavage | | 1 |
| Ghi-miRN1404i | GhRAD16 | 5 | -1 | 1 | 21 | 1995 | 2015 | AAUGGAGGAGUUGGAAAGAUU | :::::.::.: :::::: | UCUCUUUUCAGCCCCUCCAA | Cleavage | | 1 |
| Ghi-miRN1404j | GhRAD8 | 5 | -1 | 1 | 21 | 1995 | 2015 | AAUGGAGGAGUUGGAAAGAUU | :::::.::.: :::::: | UCUCUUUUCAGCCCCUCCAA | Cleavage | | 1 |
| Ghi-miRN1404j | GhRAD16 | 5 | -1 | 1 | 21 | 1995 | 2015 | AAUGGAGGAGUUGGAAAGAUU | :::::.::.: :::::: | UCUCUUUUCAGCCCCUCCAA | Cleavage | | 1 |
| Ghi-miRN1404k | GhRAD8 | 5 | -1 | 1 | 21 | 1995 | 2015 | AAUGGAGGAGUUGGAAAGAUU | :::::.::.: :::::: | UCUCUUUUCAGCCCCUCCAA | Cleavage | | 1 |
| Ghi-miRN1404k | GhRAD16 | 5 | -1 | 1 | 21 | 1995 | 2015 | AAUGGAGGAGUUGGAAAGAUU | :::::.::.: :::::: | UCUCUUUUCAGCCCCUCCAA | Cleavage | | 1 |
| Ghi-miRN1404m | GhRAD8 | 5 | -1 | 1 | 21 | 1995 | 2015 | AAUGGAGGAGUUGGAAAGAUU | :::::.::.: :::::: | UCUCUUUUCAGCCCCUCCAA | Cleavage | | 1 |
| Ghi-miRN1404m | GhRAD16 | 5 | -1 | 1 | 21 | 1995 | 2015 | AAUGGAGGAGUUGGAAAGAUU | :::::.::.: :::::: | UCUCUUUUCAGCCCCUCCAA | Cleavage | | 1 |
| Ghi-miRN1404n | GhRAD8 | 5 | -1 | 1 | 21 | 1995 | 2015 | AAUGGAGGAGUUGGAAAGAUU | :::::.::.: :::::: | UCUCUUUUCAGCCCCUCCAA | Cleavage | | 1 |
| Ghi-miRN1404n | GhRAD16 | 5 | -1 | 1 | 21 | 1995 | 2015 | AAUGGAGGAGUUGGAAAGAUU | :::::.::.: :::::: | UCUCUUUUCAGCCCCUCCAA | Cleavage | | 1 |

| | | | | | | | | | | | | | |
|---|---|---|---|---|---|---|---|---|---|---|---|---|---|
| Ghi-miRN1406a | GhRAD9 | 5 | -1 | 1 | 22 | 908 | 929 | GAACUAGGACGGUCUGAGGCUU | ::::.. ..:::..::::: | CUGCCUUGCGUCGUUUUAGUUG | Cleavage | | 1 |
| Ghi-miRN1406a | GhRAD13 | 5 | -1 | 1 | 22 | 3399 | 3419 | GAACUAGGACGGUCUGAGGCUU | ::::: :. ::.::.:::: | UUCCCUCA-AUAGUUCUGGUUC | Translation | | 1 |
| Ghi-miRN1406a | GhRAD4 | 5 | -1 | 1 | 22 | 3387 | 3407 | GAACUAGGACGGUCUGAGGCUU | ::::: :. ::.::.:::: | UUCCCUCA-AUAGUUCUGGUUC | Translation | | 1 |
| Ghi-miRN1406b | GhRAD9 | 5 | -1 | 1 | 22 | 908 | 929 | GAACUAGGACGGUCUGAGGCUU | ::::.. ..:::..::::: | CUGCCUUGCGUCGUUUUAGUUG | Cleavage | | 1 |
| Ghi-miRN1406b | GhRAD13 | 5 | -1 | 1 | 22 | 3399 | 3419 | GAACUAGGACGGUCUGAGGCUU | ::::: :. ::.::.:::: | UUCCCUCA-AUAGUUCUGGUUC | Translation | | 1 |
| Ghi-miRN1406b | GhRAD4 | 5 | -1 | 1 | 22 | 3387 | 3407 | GAACUAGGACGGUCUGAGGCUU | ::::: :. ::.::.:::: | UUCCCUCA-AUAGUUCUGGUUC | Translation | | 1 |
| Ghi-miRN1406c | GhRAD9 | 5 | -1 | 1 | 22 | 908 | 929 | GAACUAGGACGGUCUGAGGCUU | ::::.. ..:::..::::: | CUGCCUUGCGUCGUUUUAGUUG | Cleavage | | 1 |
| Ghi-miRN1406c | GhRAD13 | 5 | -1 | 1 | 22 | 3399 | 3419 | GAACUAGGACGGUCUGAGGCUU | ::::: :. ::.::.:::: | UUCCCUCA-AUAGUUCUGGUUC | Translation | | 1 |
| Ghi-miRN1406c | GhRAD4 | 5 | -1 | 1 | 22 | 3387 | 3407 | GAACUAGGACGGUCUGAGGCUU | ::::: :. ::.::.:::: | UUCCCUCA-AUAGUUCUGGUUC | Translation | | 1 |
| Ghi-miRN1406d | GhRAD9 | 5 | -1 | 1 | 22 | 908 | 929 | GAACUAGGACGGUCUGAGGCUU | ::::.. ..:::..::::: | CUGCCUUGCGUCGUUUUAGUUG | Cleavage | | 1 |
| Ghi-miRN1406d | GhRAD13 | 5 | -1 | 1 | 22 | 3399 | 3419 | GAACUAGGACGGUCUGAGGCUU | ::::: :. ::.::.:::: | UUCCCUCA-AUAGUUCUGGUUC | Translation | | 1 |
| Ghi-miRN1406d | GhRAD4 | 5 | -1 | 1 | 22 | 3387 | 3407 | GAACUAGGACGGUCUGAGGCUU | ::::: :. ::.::.:::: | UUCCCUCA-AUAGUUCUGGUUC | Translation | | 1 |
| Ghi-miRN1412a | GhRAD17 | 5 | -1 | 1 | 22 | 131 | 152 | CUUCGGGCUGAGUUAUAUAGAU | : : :.:.::: :.::::: | UAAUUUUUGAUUCACCUCGAAG | Cleavage | | 1 |
| Ghi-miRN1412b | GhRAD17 | 5 | -1 | 1 | 22 | 131 | 152 | CUUCGGGCUGAGUUAUAUAGAU | : : :.:.::: :.::::: | UAAUUUUUGAUUCACCUCGAAG | Cleavage | | 1 |
| Ghi-miRN1413 | GhRAD5 | 5 | -1 | 1 | 21 | 2426 | 2446 | AAACGGCUUAGAACCAUCUCC | .:::::::. .::..::: | UUGGAUGGUUUAGAGUUGUUC | Translation | | 1 |

| | | | | | | | | | | | | | |
|---|---|---|---|---|---|---|---|---|---|---|---|---|---|
| Ghi-miRN1415 | GhRAD 10 | 5 | -1 | 1 | 22 | 388 | 409 | UAGAGUUCACCUGGCUAUCAUC | :::: :::::: : .:::.:: | AAUGAAAGCCAGUUUGACUUUA | Translation | | 1 |
| Ghi-miRN1419b | GhRAD 15 | 5 | -1 | 1 | 22 | 3115 | 3136 | CUGAAUCUAGUUUCUCUCUUGC | :.:::::::::::. : :.: | ACGAGAGAGAAACUGGUAUUAU | Cleavage | | 1 |
| Ghi-miRN1419b | GhRAD 10 | 5 | -1 | 1 | 22 | 2282 | 2303 | CUGAAUCUAGUUUCUCUCUUGC | :.:. :::::. ::::::: | UGAGGGAAGAAAUCAGAUUCAU | Cleavage | | 1 |
| Ghi-miRN1419b | GhRAD 1 | 5 | -1 | 1 | 22 | 2159 | 2180 | CUGAAUCUAGUUUCUCUCUUGC | :.:. :::::. ::::::: | UGAGGGAAGAAAUCAGAUUCAU | Cleavage | | 1 |
| Ghi-miRN1419b | GhRAD 9 | 5 | -1 | 1 | 22 | 2209 | 2229 | CUGAAUCUAGUUUCUCUCUUGC | :.::: :: :::::.:::: :: | GUAAGUGA-AAACUGGAUUGAG | Cleavage | | 1 |
| Ghi-miRN1422 | GhRAD 10 | 5 | -1 | 1 | 21 | 616 | 636 | GGUCAAUCAUUGUUUCUGCUC | : :: ::. ::::.:::.:: | UACCAAAAGGAAUGGUUGGCC | Cleavage | | 1 |
| Ghi-miRN1422 | GhRAD 1 | 5 | -1 | 1 | 21 | 493 | 513 | GGUCAAUCAUUGUUUCUGCUC | : :: ::. ::::.:::.:: | UACCAAAAGGAAUGGUUGGCC | Cleavage | | 1 |
| Ghi-miRN1425 | GhRAD 10 | 5 | -1 | 1 | 21 | 2027 | 2047 | CUUUUUAGGGAUCAUGGCACC | ::: :::::::. :.::: | UUUGCAAUGAUCCUGCAGAAG | Cleavage | | 1 |
| Ghi-miRN1425 | GhRAD 1 | 5 | -1 | 1 | 21 | 1904 | 1924 | CUUUUUAGGGAUCAUGGCACC | ::: :::::::. :.::: | UUUGCAAUGAUCCUGCAGAAG | Cleavage | | 1 |
| Ghi-miRN1426a | GhRAD 15 | 5 | -1 | 1 | 21 | 2974 | 2993 | UGACGUGGACAAAAUGCUCCC | ::::::: :::.:: :::: | AAGAGCAUUGUGUUCA-GUCA | Cleavage | | 1 |
| Ghi-miRN1426a | GhRAD 6 | 5 | -1 | 1 | 21 | 2935 | 2954 | UGACGUGGACAAAAUGCUCCC | ::::::: :::.:: :::: | AAGAGCAUUGUGUUCA-GUCA | Cleavage | | 1 |
| Ghi-miRN1426b | GhRAD 15 | 5 | -1 | 1 | 21 | 2974 | 2993 | UGACGUGGACAAAAUGCUCCC | ::::::: :::.:: :::: | AAGAGCAUUGUGUUCA-GUCA | Cleavage | | 1 |
| Ghi-miRN1426b | GhRAD 6 | 5 | -1 | 1 | 21 | 2935 | 2954 | UGACGUGGACAAAAUGCUCCC | ::::::: :::.:: :::: | AAGAGCAUUGUGUUCA-GUCA | Cleavage | | 1 |
| Ghi-miRN1426c | GhRAD 15 | 5 | -1 | 1 | 21 | 2974 | 2993 | UGACGUGGACAAAAUGCUCCC | ::::::: :::.:: :::: | AAGAGCAUUGUGUUCA-GUCA | Cleavage | | 1 |
| Ghi-miRN1426c | GhRAD 6 | 5 | -1 | 1 | 21 | 2935 | 2954 | UGACGUGGACAAAAUGCUCCC | ::::::: :::.:: :::: | AAGAGCAUUGUGUUCA-GUCA | Cleavage | | 1 |

| | | | | | | | | | | | | | |
|---|---|---|---|---|---|---|---|---|---|---|---|---|---|
| Ghi-miRN1426d | GhRAD15 | 5 | -1 | 1 | 21 | 2974 | 2993 | UGACGUGGACAAAAUGCUCCC | ::::::: :::.:: :::: | AAGAGCAUUGUGUUCA-GUCA | Cleavage | | 1 |
| Ghi-miRN1426d | GhRAD6 | 5 | -1 | 1 | 21 | 2935 | 2954 | UGACGUGGACAAAAUGCUCCC | ::::::: :::.:: :::: | AAGAGCAUUGUGUUCA-GUCA | Cleavage | | 1 |
| Ghi-miRN1426e | GhRAD15 | 5 | -1 | 1 | 21 | 2974 | 2993 | UGACGUGGACAAAAUGCUCCC | ::::::: :::.:: :::: | AAGAGCAUUGUGUUCA-GUCA | Cleavage | | 1 |
| Ghi-miRN1426e | GhRAD6 | 5 | -1 | 1 | 21 | 2935 | 2954 | UGACGUGGACAAAAUGCUCCC | ::::::: :::.:: :::: | AAGAGCAUUGUGUUCA-GUCA | Cleavage | | 1 |
| Ghi-miRN1426f | GhRAD15 | 5 | -1 | 1 | 21 | 2974 | 2993 | UGACGUGGACAAAAUGCUCCC | ::::::: :::.:: :::: | AAGAGCAUUGUGUUCA-GUCA | Cleavage | | 1 |
| Ghi-miRN1426f | GhRAD6 | 5 | -1 | 1 | 21 | 2935 | 2954 | UGACGUGGACAAAAUGCUCCC | ::::::: :::.:: :::: | AAGAGCAUUGUGUUCA-GUCA | Cleavage | | 1 |
| Ghi-miRN1435 | GhRAD15 | 5 | -1 | 1 | 21 | 361 | 381 | UUUAGAAAUCAUUCCUUCCUU | ::::.:: ::::. ::.::: | AAGGGAGAAAUGGGUUUUAAU | Cleavage | | 1 |
| Ghi-miRN1435 | GhRAD7 | 5 | -1 | 1 | 21 | 302 | 322 | UUUAGAAAUCAUUCCUUCCUU | :: .::::::::: ::. | GUGGUGGGAAUGAUUUGGAAG | Cleavage | | 1 |
| Ghi-miRN1438 | GhRAD15 | 5 | -1 | 1 | 21 | 359 | 379 | UGAAUCUAGUUUCUCUCUUAC | :::.::::::. .: ::.: | UGAAGGGAGAAAUGGGUUUUA | Cleavage | | 3 |
| Ghi-miRN1443a | GhRAD8 | 5 | -1 | 1 | 21 | 2452 | 2471 | UCUUUGAUGAUGUUACUGACC | : ::.: :::: :::...:::: | GCUCGG-AACAGCAUUGAAGA | Translation | | 2 |
| Ghi-miRN1443b | GhRAD8 | 5 | -1 | 1 | 21 | 2452 | 2471 | UCUUUGAUGAUGUUACUGACC | : ::.: :::: :::...:::: | GCUCGG-AACAGCAUUGAAGA | Translation | | 2 |
| Ghi-miRN1448 | GhRAD13 | 5 | -1 | 1 | 20 | 212 | 231 | CAGCAAUUCGCGCUGACGUG | :. :::: :.::.::::: | AAUUUCAGAGUGAGUUGCUU | Cleavage | | 1 |
| Ghi-miRN1448 | GhRAD4 | 5 | -1 | 1 | 20 | 206 | 225 | CAGCAAUUCGCGCUGACGUG | :. :::: :.::.::::: | AAUUUCAGAGUGAGUUGCUU | Cleavage | | 1 |
| Ghi-miRN1449a | GhRAD2 | 5 | -1 | 1 | 21 | 1507 | 1527 | GGAAGGUUUGGAGGAGAGUGA | :.: ::.::..::..::: | GGAUUAUCUUCUGAAUUUUCA | Cleavage | | 1 |
| Ghi-miRN1449a | GhRAD11 | 5 | -1 | 1 | 21 | 361 | 381 | GGAAGGUUUGGAGGAGAGUGA | :.: ::.::..::..::: | GGAUUAUCUUCUGAAUUUUCA | Cleavage | | 1 |

| | | | | | | | | | | | | | |
|---|---|---|---|---|---|---|---|---|---|---|---|---|---|
| Ghi-miRN1449a | GhRAD15 | 5 | -1 | 1 | 21 | 102 | 122 | GGAAGGUUUGGAGGAGAGUGA | :: :: :.:::::::: | CGACCCUGAUUCAAACCUUCA | Cleavage | | 1 |
| Ghi-miRN1449b | GhRAD9 | 5 | -1 | 1 | 21 | 1065 | 1085 | GGAAGGUUUGGAGGAGAUUGA | :...::::.::. :::.::.. | UUGGUCUCUUCUUAACUUUUU | Cleavage | | 1 |
| Ghi-miRN1449b | GhRAD14 | 5 | -1 | 1 | 21 | 2379 | 2399 | GGAAGGUUUGGAGGAGAUUGA | :::.:::...:...:: :: | UGCUUCUUCUCUGGAUUUUCC | Cleavage | | 1 |
| Ghi-miRN1449b | GhRAD5 | 5 | -1 | 1 | 21 | 2298 | 2318 | GGAAGGUUUGGAGGAGAUUGA | :::.:::...:...:: :: | UGCUUCUUCUCUGGAUUUUCC | Cleavage | | 1 |
| Ghi-miRN1449b | GhRAD8 | 5 | -1 | 1 | 21 | 45 | 66 | GGAAGGUUUGGAGGA-GAUUGA | :::: ::.::: :...::::: | AGAAUCCUCUUCCCAGUCUUCC | Cleavage | | 1 |
| Ghi-miRN1451 | GhRAD8 | 5 | -1 | 1 | 21 | 250 | 270 | CAACGGUGGAGGUAUUGUGCU | :.: .:::.:.:.:: ::: | GCCGCCGUACUUUCGCCUUUG | Cleavage | | 1 |
| Ghi-miRN1451 | GhRAD16 | 5 | -1 | 1 | 21 | 250 | 270 | CAACGGUGGAGGUAUUGUGCU | :.: .:::.:.:.:: ::: | GCCGCCGUACUUUCGCCUUUG | Cleavage | | 1 |
| Ghi-miRN1453 | GhRAD17 | 5 | -1 | 1 | 21 | 644 | 664 | CACCCACCACAGCAGCAGCUC | :::: : : :::: :::::: | CAGCUCCGGAUGUGUUGGGUG | Cleavage | | 1 |
| Ghi-miRN1454a | GhRAD8 | 5 | -1 | 1 | 21 | 276 | 296 | UCGAUGAUAACUGGGUGAACU | ::::... ::.::.:::: | CUCUCACUUGAUUGUCGUCGA | Translation | | 1 |
| Ghi-miRN1454a | GhRAD16 | 5 | -1 | 1 | 21 | 276 | 296 | UCGAUGAUAACUGGGUGAACU | ::::... ::.::.:::: | CUCUCACUUGAUUGUCGUCGA | Translation | | 1 |
| Ghi-miRN1454b | GhRAD8 | 5 | -1 | 1 | 21 | 276 | 296 | UCGAUGAUAACUGGGUGAACU | ::::... ::.::.:::: | CUCUCACUUGAUUGUCGUCGA | Translation | | 1 |
| Ghi-miRN1454b | GhRAD16 | 5 | -1 | 1 | 21 | 276 | 296 | UCGAUGAUAACUGGGUGAACU | ::::... ::.::.:::: | CUCUCACUUGAUUGUCGUCGA | Translation | | 1 |
| Ghi-miRN1455 | GhRAD13 | 5 | -1 | 1 | 21 | 2554 | 2574 | UCUUCGAACUUUUGAGAGACU | :::. ::::::: :: :. | CCUCUUGCAAAAGUUGGAUGG | Cleavage | | 1 |
| Ghi-miRN1455 | GhRAD4 | 5 | -1 | 1 | 21 | 2542 | 2562 | UCUUCGAACUUUUGAGAGACU | :::. ::::::: :: :. | CCUCUUGCAAAAGUUGGAUGG | Cleavage | | 1 |
| Ghi-miRN1455 | GhRAD14 | 5 | -1 | 1 | 21 | 2494 | 2514 | UCUUCGAACUUUUGAGAGACU | :::. ::::::: :: :. | CCUCUUGCAAAAGUUGGAUGG | Cleavage | | 1 |

| | | | | | | | | | | | | | |
|---|---|---|---|---|---|---|---|---|---|---|---|---|---|
| Ghi-miRN1455 | GhRAD5 | 5 | -1 | 1 | 21 | 2413 | 2433 | UCUUCGAACUUUUGAGAGACU | :::. :::::::: :: :. | CCUCUUGCAAAAGUUGGAUGG | Cleavage | | 1 |
| Ghi-miRN1456 | GhRAD2 | 5 | -1 | 1 | 21 | 2957 | 2977 | UUCACCCAGAGUCCGAGAGCU | ::.:.:::: .: ::::.: | UCCUUUUGGACCUUUGGUGGA | Translation | | 1 |
| Ghi-miRN1461 | GhRAD7 | 5 | -1 | 1 | 21 | 2705 | 2725 | CAGGAAGAGGAAGAUGAAAUA | : :: :: ::::::::.:: | UCUUGCAGAUUCCUCUUUCUC | Cleavage | | 1 |
| Ghi-miRN1461 | GhRAD17 | 5 | -1 | 1 | 21 | 2705 | 2725 | CAGGAAGAGGAAGAUGAAAUA | : :: :: ::::::::.:: | UCUUGCAGAUUCCUCUUUCUC | Cleavage | | 1 |
| Ghi-miRN1462 | GhRAD7 | 5 | -1 | 1 | 21 | 1860 | 1880 | AAAGAUUGGAGGAGUUGGUAA | ::: . :::::::. :.::: | AUACAGUCUCCUCCGCUUUUU | Cleavage | | 1 |
| Ghi-miRN1462 | GhRAD17 | 5 | -1 | 1 | 21 | 1860 | 1880 | AAAGAUUGGAGGAGUUGGUAA | ::: . :::::::. :.::: | AUACAGUCUCCUCCGCUUUUU | Cleavage | | 1 |
| Ghi-miRN1468 | GhRAD10 | 5 | -1 | 1 | 21 | 1467 | 1487 | UGAAUUGGAGAGUGAAGUCAU | ::: .:::::....:::. | AGAACUGUACUCUUUGGUUCG | Cleavage | | 1 |
| Ghi-miRN1468 | GhRAD1 | 5 | -1 | 1 | 21 | 1344 | 1364 | UGAAUUGGAGAGUGAAGUCAU | ::: .:::::....:::. | AGAACUGUACUCUUUGGUUCG | Cleavage | | 1 |
| Ghi-miRN1470 | GhRAD15 | 5 | -1 | 1 | 21 | 278 | 298 | UUGUUGUAGAAGCGUCGCUAA | ::.:::::::: :..: :. | ACAGUGACGCUUCCAUGAAAG | Cleavage | | 1 |
| Ghi-miRN1470 | GhRAD6 | 5 | -1 | 1 | 21 | 278 | 298 | UUGUUGUAGAAGCGUCGCUAA | ::.:::::::: :..: :. | ACAGUGACGCUUCCAUGAAAG | Cleavage | | 1 |
| Ghi-miRN1474c | GhRAD2 | 5 | -1 | 1 | 21 | 903 | 923 | UUGUGAUGUUUGUGAGGAACA | ::.:: ::.:::::: :: | UCCUCUUCUCAGACAUCAGAA | Cleavage | | 1 |
| Ghi-miRN1474c | GhRAD16 | 5 | -1 | 1 | 21 | 1057 | 1077 | UUGUGAUGUUUGUGAGGAACA | : ::: :::....:.: ::::. | UUUUCAUCAUGGAUAACACAG | Cleavage | | 1 |
| Ghi-miRN1480a | GhRAD15 | 5 | -1 | 1 | 21 | 1338 | 1358 | UUGUGAUGUGGUUUGAAUUUC | ::.:: :..::..:::: :: | CAAGUUGAGGCCGUAUCAGAA | Cleavage | | 2 |
| Ghi-miRN1480a | GhRAD6 | 5 | -1 | 1 | 21 | 1338 | 1358 | UUGUGAUGUGGUUUGAAUUUC | ::.:: :..::..:::: :: | CAAGUUGAGGCCGUAUCAGAA | Cleavage | | 2 |
| Ghi-miRN1480b | GhRAD15 | 5 | -1 | 1 | 21 | 1338 | 1358 | UUGUGAUGUGGUUUGAAUUUC | ::.:: :..::..:::: :: | CAAGUUGAGGCCGUAUCAGAA | Cleavage | | 2 |

| | | | | | | | | | | | | | |
|---|---|---|---|---|---|---|---|---|---|---|---|---|---|
| Ghi-miRN1480b | GhRAD6 | 5 | -1 | 1 | 21 | 1338 | 1358 | UUGUGAUGUGGUUUGAAUUUC | ::.:: :..::..:::: :: | CAAGUUGAGGCCGUAUCAGAA | Cleavage | | 2 |
| Ghi-miRN1482 | GhRAD17 | 5 | -1 | 1 | 21 | 671 | 691 | UUGAUGGAAGAAGAAUAGUAG | ::::.:::: .: .:::.:: | AUACUGUUCUCUUAUCAUUAA | Translation | | 1 |
| Ghi-miRN1490 | GhRAD9 | 5 | -1 | 1 | 22 | 2173 | 2194 | CUAACUUGUCUUCGCCCUUCUC | :::: .::. ::::::: : | UUAAAGGAUGAGAACAAGUUUG | Translation | | 1 |
| Ghi-miRN1492a | GhRAD8 | 5 | -1 | 1 | 22 | 1043 | 1064 | CACGAAGACAUAGGUAGGGAUG | :::: .::.:::.::: : | GCCCCCUCUCUGUGUUUUCAUC | Cleavage | | 1 |
| Ghi-miRN1492b | GhRAD8 | 5 | -1 | 1 | 22 | 1043 | 1064 | CACGAAGACAUAGGUAGGGAUG | :::: .::.:::.::: : | GCCCCCUCUCUGUGUUUUCAUC | Cleavage | | 1 |
