# Supplementary material for: RAD gene family analysis in cotton provides some key genes for flowering and stress tolerance in upland cotton G. hirsutum
Source: BMC Genomics. 2022 Jan 10;23:40. doi: 10.1186/s12864-021-08248-z (PMC8744286; doi:10.1186/s12864-021-08248-z)
Supplement: Supplementary file 12 — Additional file 12 : Table S6. GhRAD genes primers for qRT-PCR. [file 12864_2021_8248_MOESM12_ESM.pdf]

Additional file 12: Table S6. *GhRAD* genes primers for qRT-PCR.

	Forward Primer	Reverse Primer
1	GhRAD1	GCAGCTTCGATCGCGTAAAAGG
2	GhRAD2	CAACTCACTAGCTGACCGACCG
3	GhRAD3	TTGCCATCTGTCTTGTCTGCCA
4	GhRAD4	TTGATGCTTTTGCGTCTTCGCC
5	GhRAD9	GTGTTCCGCTTTTGCACAGAGT
6	GhRAD10	ACGTGGCTACGCCAATACAACA
7	GhRAD11	CAACTCACTAGCTGACCGACCG
8	GhRAD12	GTGCTGGTGCCCTTGCTAGAGT
