# Supplementary material for: RAD gene family analysis in cotton provides some key genes for flowering and stress tolerance in upland cotton G. hirsutum
Source: BMC Genomics. 2022 Jan 10;23:40. doi: 10.1186/s12864-021-08248-z (PMC8744286; doi:10.1186/s12864-021-08248-z)
Supplement: Supplementary file 13 — Additional file 13 : Table S7. Proposed name of RAD gene family members. [file 12864_2021_8248_MOESM13_ESM.pdf]

Additional file 13: Table S7. Proposed name of *RAD* gene family members.

Arabidopsis	Proposed name	G. hirsutum (AD1)	proposed name	G. barbadense (AD2)	proposed name	G. herbaceum (A1)	proposed name	G. arboreum (A2)	proposed name	G. raimondii (D5)	proposed name
AT1G02670.1	AtRAD1	Gh_A03G0848.1	GhRAD1	Gbar_A03G011030.2	GbRAD1	Ghe03G05870	GheRAD1	evm.model.Ga03G1347	GaRAD1	Gorai.005G058400.2.v2.1	GrRAD1
AT1G05120.1	AtRAD2	Gh_A03G1475.1	GhRAD2	Gbar_A03G018540.1	GbRAD2	Ghe03G15300	GheRAD2	evm.model.Ga03G2221	GaRAD2	Gorai.005G137100.1.v2.1	GrRAD2
AT1G11100.1	AtRAD3	Gh_A04G1416.1	GhRAD3	Gbar_A04G011680.1	GbRAD3	Ghe04G19770	GheRAD3	evm.model.Ga04G0395	GaRAD3	Gorai.005G213400.1.v2.1	GrRAD3
AT1G50410.1	AtRAD4	Gh_A05G1384.1	GhRAD4	Gbar_A05G016300.2	GbRAD4	Ghe05G17650	GheRAD4	evm.model.Ga05G1742	GaRAD4	Gorai.006G248800.1.v2.1	GrRAD4
AT1G61140.1	AtRAD5	Gh_A06G1100.1	GhRAD5	Gbar_A06G013640.2	GbRAD5	Ghe06G17300	GheRAD5	evm.model.Ga06G1514	GaRAD5	Gorai.009G171000.1.v2.1	GrRAD5
AT3G16600.1	AtRAD6	Gh_A09G1973.1	GhRAD6	Gbar_A09G024160.1	GbRAD6	Ghe09G28520	GheRAD6	evm.model.Ga09G2568	GaRAD6	Gorai.010G151100.1.v2.1	GrRAD6
AT3G20010.1	AtRAD7	Gh_A13G1937.1	GhRAD7	Gbar_A13G000140.1	GbRAD7	Ghe13G00150	GheRAD7	evm.model.Ga13G0013	GaRAD7	Gorai.012G136000.1.v2.1	GrRAD7
AT5G05130.1	AtRAD8	Gh_A13G2075.1	GhRAD8	Gbar_A13G023790.1	GbRAD8	Ghe13G28890	GheRAD8	evm.model.Ga13G2713	GaRAD8	Gorai.013G001200.1.v2.1	GrRAD8
AT5G22750.1	AtRAD9	Gh_D02G0515.1	GhRAD9	Gbar_D02G012520.3	GbRAD9					Gorai.013G258600.1.v2.1	GrRAD9
AT5G43530.1	AtRAD10	Gh_D02G1162.1	GhRAD10	Gbar_D02G020440.4	GbRAD10						
		Gh_D02G1943.1	GhRAD11	Gbar_D04G016350.3	GbRAD11						
		Gh_D04G1466.1	GhRAD12	Gbar_D05G016700.2	GbRAD12						
		Gh_D05G1552.1	GhRAD13	Gbar_D06G013890.1	GbRAD13						
		Gh_D06G1350.1	GhRAD14	Gbar_D09G023820.1	GbRAD14						
		Gh_D09G2176.1	GhRAD15	Gbar_D13G024370.1	GbRAD15						
		Gh_D13G0012.1	GhRAD16	Gbar_D13G025800.1	GbRAD16						
		Gh_D13G2334.1	GhRAD17								
