# Supplementary material for: RAD gene family analysis in cotton provides some key genes for flowering and stress tolerance in upland cotton G. hirsutum
Source: BMC Genomics. 2022 Jan 10;23:40. doi: 10.1186/s12864-021-08248-z (PMC8744286; doi:10.1186/s12864-021-08248-z)
Supplement: Supplementary file 1 — Additional file 1 : Figure S1. Phylogenetic relationship between G. hirsutum and Arabidopsis. Phylogenetic tree was constructed using RAD protein sequences by iTOL software. [file 12864_2021_8248_MOESM1_ESM.pdf]

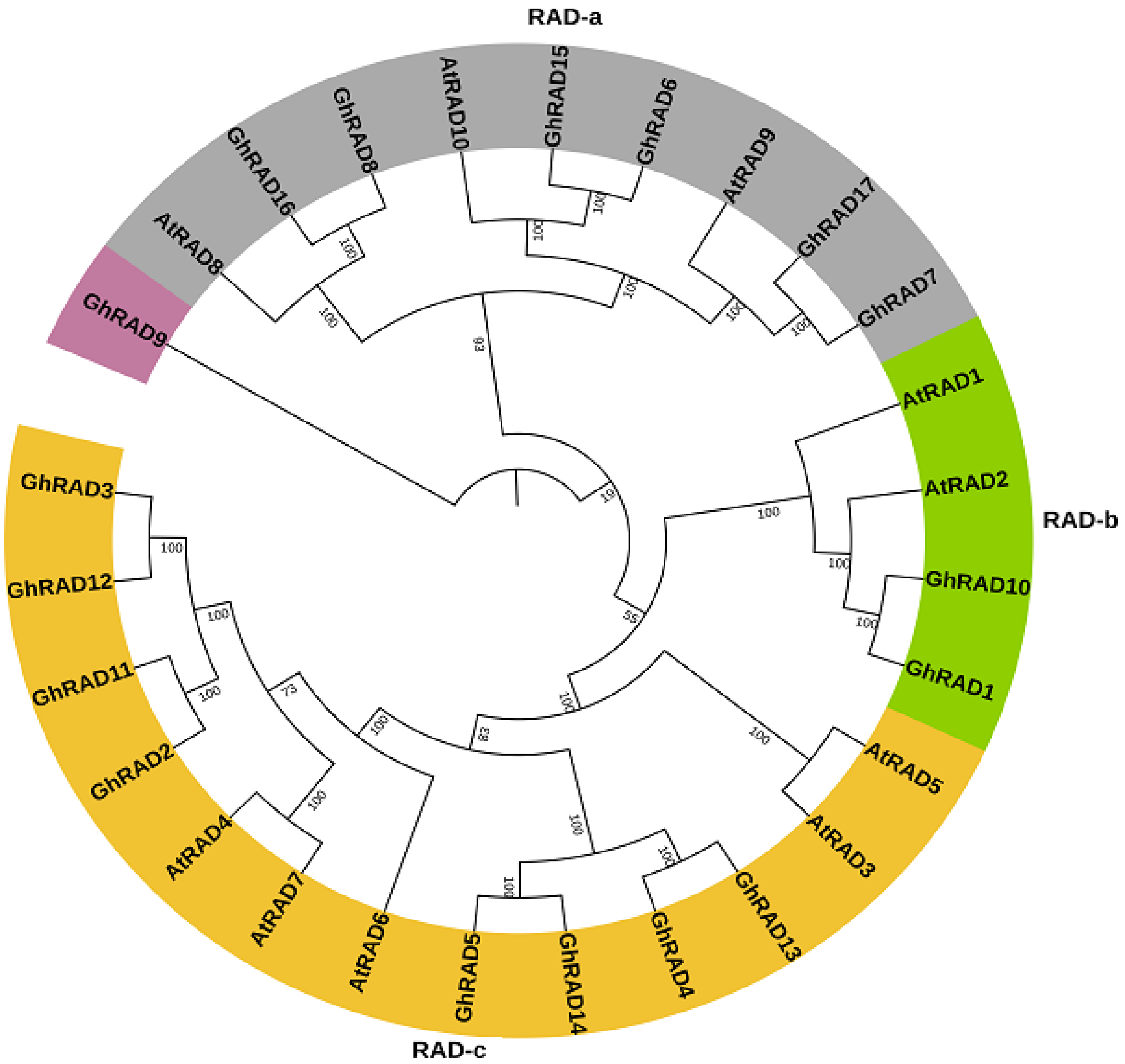

Additional file 1: Figure S1. Phylogenetic relationship between *G. hirsutum* and *Arabidopsis.* Phylogenetic tree was constructed using RAD protein sequences by iTOL software.
